# Global, regional, and national burden of ischemic heart disease attributable to lead exposure, 1990–2021: decomposition, frontier, and projection analysis

**DOI:** 10.3389/fpubh.2025.1567747

**Published:** 2025-08-18

**Authors:** Xinyue Wen, Lichun Qiao, Feidan Deng, Jingxuan Zhou, Miaoqian Li, Lin Wang, Huan Deng, Abebe Feyissa Amhare, Jing Han, Yijie Guo

**Affiliations:** ^1^Department of Psychiatry, The First Affiliated Hospital of Xi'an Jiaotong University, Xi An, Shaanxi, China; ^2^School of Public Health, Xi'an Jiaotong University Health Science Center, Xi An, Shaanxi, China; ^3^School of Urban Planning and Design, Peking University Shenzhen Graduate School, Shenzhen, Guangdong, China; ^4^The Fifth Clinical Medical College, Chongqing Medical University, Chongqing, China; ^5^The Second Affiliated Hospital, Xi'an Jiaotong University Health Science Center, Xi An, Shaanxi, China

**Keywords:** ischemic heart disease, lead exposure, global burden, death, disability-adjusted life years

## Abstract

**Introduction:**

Ischemic heart disease (IHD) is a leading global health burden, with lead exposure identified as a significant environment risk factor contributing to its prevalence.

**Methods:**

Data from the Global Burden of Disease Study (GBD) 2021 were used to analyze deaths and disability-adjusted life years (DALYs) of IHD due to lead exposure at global, regional, and national levels. Decomposition analysis, frontier analysis, and Bayesian age-period cohort (BAPC) models were applied to assess trends from 1990 to 2021.

**Results:**

In 2021, deaths and DALYs attributable to lead exposure reached 590,370.03 and 11,854,611.43, respectively, though age-standardized rates (ASRs) declined. Males and the older adult exhibited higher ASRs. At regional level, South Asia had the highest number of deaths and DALYs, while North Africa and the Middle East had the highest ASRs. Certain countries showed increasing ASRs over time, with a negative correlation between socio-demographic index (SDI) and ASRs. Decomposition analysis identified that population growth as the primary driver of increasing deaths and DALYs, particularly in middle-SDI regions. Frontier analysis suggested that middle and low-SDI regions have the greater potential to reduce the IHD burden. BAPC projections indicated a global decrease in IHD burden due to lead exposure by 2050.

**Conclusions:**

The burden remains disproportionately high in males, the older adult and low- and middle-SDI regions, highlighting the need for targeted prevention and lead exposure control efforts in these populations.

## 1 Introduction

Ischemic heart disease (IHD), characterized by atherosclerosis as the primary pathological process, remains a major non-communicable disease worldwide (1, 2). Ischemic symptoms such as unstable angina can present in IHD, and severe cases may lead to life-threatening myocardial infarction (3). In 2021, IHD accounted for 8.99 million deaths globally, making it the leading cause of standardized deaths and imposing a substantial burden on healthcare systems (4, 5). High systolic blood pressure, high fasting plasma glucose, high low-density lipoprotein cholesterol, and high body mass index were traditional risk factors for IHD (6). In recent years, non-traditional risk factors leading to IHD have received increasing attention, such as environmental pollution (7).

Lead is a prevalent environmental contaminant, with exposure occurring through food, water, and inhalation (8). Its widespread use in industries such as manufacturing, construction, plastics, and chemicals has resulted in lead contamination in air, soil, dust, and water (9). Chronic lead exposure can lead to its accumulation in various tissues, including hair, nails, bones, blood, and teeth, with the majority stored in bones and teeth (10). Lead is recognized by World Health Organization as one of the chemicals that present a significant public health concern and can impact various body systems, including the neurological, hematological, gastrointestinal, cardiovascular, and renal systems (11).

Lead is a cardiotoxic metal and its effects on the heart involve multiple mechanisms (12). Previous research has demonstrated that developmental Pb^2^^+^ exposure occurring early in life leads to Pb^2^^+^-induced cardiopathology in later life via mechanisms characterized by induced hypertension and reduced elasticity of the aortic media (13). Lead can affect cardiac function through humoral and neuronal dysregulation, and can also cause cardiac damage by entering cardiomyocytes and interfering with redox mechanisms and calcium signaling and homeostasis (14). Epidemiologic findings indicated that in 2019, lead exposure caused 901,720 deaths and 21,680,000 disability-adjusted life years (DALYs) globally, with IHD accounting for the largest proportion of the 13 diseases caused by lead exposure (15). A study from China revealed that the burden of IHD attributable to lead exposure is substantial, with 105,857 deaths and 1,899,139 DALYs recorded (16). In addition, the burden of lead attributable cardiovascular disease was also been quantified in the United States, and the results showed that the major contributor to the burden of cardiovascular disease deaths associated with lead exposure was IHD (17). Wang et al. (18) found a non-linear correlation between blood lead and subclinical myocardial injury, suggesting that lead may play a detrimental role earlier in the cardiovascular disease continuum. The Third National Health and Nutrition Examination Survey found that blood lead concentrations below the action level for U.S. adults (5 μg/dl or 0.24 μmol/L) were associated with an increased risk of death from IHD (19).

Despite these findings, most of the current research focuses on specific regions or countries and lacks a comprehensive and accurate description of the global burden and trends of IHD due to lead exposure. This study uses the latest GBD 2021 database and multiple statistical methods (e.g., decomposition analysis, frontier analysis, and Bayesian age-period-cohort modeling) to comprehensively assess the global, regional, and national burden of IHD attributable to lead exposure from 1990 to 2021 and future trend projections. This analysis will provide critical data to inform lead exposure management and IHD prevention strategies.

## 2 Methods

### 2.1 Study data

Data for this study were obtained from the Global Burden of Disease Study (GBD) 2021, which provides the most comprehensive assessment of 371 diseases and injuries across 204 countries and territories (20). We accessed the Global Health Data Exchange Tool (GHDx, http://ghdx.healthdata.org/gbd-results-tool) to extract information on the burden of IHD attributable to lead exposure from 1990 to 2021. The data included the number and rate of deaths and DALYs, age-standardized mortality rate (ASMR), and age-standardized DALY rate (ASDR) per 100,000 population, along with 95% uncertainty intervals (UI) at global, regional, national, and socio-demographic index (SDI) levels. UI were derived from 1,000 Monte Carlo simulations accounting for input data variability and model uncertainty. The wide UI indicate potential limitations in exposure assessment, particularly in low-data regions. These metrics were stratified by sex and age to assess disparities. SDI quintiles (low, low-middle, middle, high-middle, high) were defined based on tertiles of income, education, and fertility metrics (20).

### 2.2 Evaluation of IHD and lead exposure

GBD 2021 estimated IHD as the aggregate of discrete sequelae, consisting of myocardial infarction (heart attacks), angina (stable IHD manifesting as chest pain), or ischemic cardiomyopathy (heart failure due to IHD) (20). Chronic lead exposure is associated with cardiovascular disease, measured as micrograms of lead per gram of bone (μg/g), and the lead exposures in this study represent chronic lead exposure. In the GBD 2021 study, data on lead exposure were mainly derived from literature reports on blood lead levels, as well as some blood lead surveys. To estimate lead exposure in bone, the researchers calculated a cumulative blood lead index for each cohort based on their estimated lead exposure over their lifetime. The cumulative blood lead index was then used in conjunction with scalar values from the literature to estimate the amount of lead in bone (21).

### 2.3 Statistical analysis

The burden of IHD due to lead exposure was quantified using the number and rate of deaths and DALYs, ASMR, ASDR, and their respective 95% UI. The estimated annual percentage change (EAPC) was calculated to evaluate trends in age-standardized rates (ASRs) over the 32-year period. If both the EAPC and the upper limit of its 95% confidence interval (CI) were < 0, the ASRs were trending downward; conversely, if both the EAPC and the lower limit of its 95% CI were >0, the ASRs were trending upward, and if the 95% CI for the EAPC included 0, it was considered a constant trend (22). Pearson correlation coefficients (*r*) with two-tailed *t*-test for significance were calculated at global, regional, and country levels to assess the SDI-burden relationship. The Pearson correlation coefficient ranges from −1 to 1, where a larger absolute value indicates a stronger association. Significance threshold: *P* < 0.05.

#### 2.3.1 Decomposition analysis

Decomposition analysis refers to the breakdown of a composite indicator (e.g., incidence, prevalence, etc.) into multiple components in order to gain a clearer understanding of the contribution of each factor to the overall outcome (23). We used a decomposition method developed by Das Gupta to examine the impact of population growth, aging, and epidemiological changes on the burden of IHD from 1990 to 2021 (24). Decomposition analysis was the application of mathematical methods to isolate the standardized effects of each multiplicative factor, which allowed us to quantify the individual contributions of each factor (population growth, aging, and epidemiological changes) to changes in IHD burden (25, 26).

#### 2.3.2 Frontier analysis

Frontier analysis was a quantitative method applied to assess the relationship between IHD burden and socio-demographic development (quantified by the SDI), determining the minimum achievable ASMR and ASDR relative to development levels measured by SDI. The Free Disposal Hull method combined with Data Envelopment Analysis was employed to draw a non-linear frontier, and Local polynomial regression was used to smooth the boundaries with a polynomial number of 1 and a span of 0.2 (27). By measuring the absolute distance (i.e., the effective difference) between the ASMR and ASDR and the borderline for each country or region, we assessed the potential for improvement in each country or region.

#### 2.3.3 Projection analysis

Bayesian age-period-cohort (BAPC) was a statistical model designed to analyze and predict the impacts of age, time period, and birth cohort on demographic outcomes such as mortality or disease incidence rates (28). The burden of IHD due to lead exposure from 2022 to 2050 was projected by integrated nested Laplace approximations using a BAPC model (29).

All analyses were performed using R 4.4.1. Statistical significance for all inferential tests was defined as two-tailed *P* < 0.05.

## 3 Results

### 3.1 Global level trends

In 2021, the global number of deaths and DALYs attributable to IHD due to lead exposure were estimated at 590,370 (95% UI: −83,778 to 12,33,628) and 11,854,611 (95% UI: −16,68,553 to 24,791,275), respectively. The ASMR and ASDR were 7.11 (95% UI: −1.01 to 14.88) and 138.57 (95% UI: −19.52 to 289.73) per 100,000 population (Table 1). Compared with 1990, the number of deaths and DALYs increased and the ASRs decreased in 2021 (Table 1).

**Table 1:** The death and DALYs cases, ASRs, and EAPC of ischemic heart disease due to lead exposure from 1990 to 2021.

**Characteristics**	**Death case**		**ASMR (per 100,000 population)**			**DALYs case**		**ASDR (per 100,000 population)**		
	**1990**	**2021**	**1990**	**2021**	**1990–2021**	**1990**	**2021**	**1990**	**2021**	**1990–2021**
	**Both (95% UI)**	**Both (95% UI)**	**Both (95% UI)**	**Both (95% UI)**	**EAPC (95% CI)**	**Both (95% UI)**	**Both (95% UI)**	**Both (95% UI)**	**Both (95% UI)**	**EAPC (95% CI)**
Global	277,681.94 (−40,555.95 to 595,671.21)	590,370.03 (−83,778.27 to 12,33,628.18)	7.91 (−1.15 to 16.94)	7.11 (−1.01 to 14.88)	−0.30 (−0.40 to −0.20)	6,484,403.92 (−947,861.76 to 14,078,198.14)	11,854,611.43 (−1,668,553.12 to 24,791,275.33)	166.11 (−24.29 to 359.91)	138.57 (−19.52 to 289.73)	−0.58 (−0.69 to −0.46)
**Sex**										
Male	166,565.57 (−24,421.42 to 357,772.10)	360,286.49 (−51,331.77 to 750,585.08)	10.80 (−1.58 to 22.98)	9.93 (−1.42 to 20.76)	−0.23 (−0.34 to −0.10)	4,187,330.29 (−613,966.41 to 9,090,864.51)	7,698,637.79 (−1,087,983.99 to 16,151,343.70)	230.62 (−33.86 to 498.48)	195.17 (−27.66 to 407.75)	−0.53 (−0.65 to −0.41)
Female	111,116.37 (−16,134.53 to 235,749.47)	230,083.54 (−32,446.50 to 491,866.67)	5.70 (−0.83 to 12.15)	4.91 (−0.69 to 10.50)	−0.44 (−0.53 to −0.34)	2,297,073.63 (−333,895.34 to 4,867,447.87)	4,155,973.64 (−580,569.13 to 8,783,636.63)	110.66 (−16.08 to 234.59)	89.67 (−12.52 to 189.54)	−0.66 (−0.76 to −0.56)
**SDI**										
High	60,041.63 (−8,490.88 to 131,072.96)	49,288.79 (−6,957.54 to 107,582.47)	5.46 (−0.77 to 11.94)	2.02 (−0.28 to 4.39)	−3.43 (−3.53 to −3.34)	1,123,443.68 (−158,080.91 to 2,423,686.82)	787,212.82 (−111,112.10 to 1,688,157.86)	102.41 (−14.39 to 221.26)	36.18 (−5.10 to 77.11)	−3.56 (−3.64 to −3.48)
High-middle	58,196.03 (−8,194.36 to 126,157.65)	118,371.91 (−16,281.86 to 251,977.06)	6.87 (−0.97 to 14.86)	6.15 (−0.85 to 13.10)	−0.44 (−0.72 to −0.16)	1,260,683.08 (−177,011.77 to 2,741,404.49)	2,027,584.60 (−275,336.78 to 4,329,965.77)	132.97 (−18.71 to 289.85)	103.45 (−14.06 to 220.96)	−0.97 (−1.29 to −0.65)
Middle	69,708.74 (−9,955.84 to 149,892.09)	204,073.43 (−28,950.46 to 424,987.74)	8.42 (−1.21 to 17.89)	8.80 (−1.25 to 18.38)	0.29 (0.14–0.44)	1,735,489.34 (−247,537.18 to 3,755,519.49)	4,012,492.20 (−563,061.67 to 8,398,080.45)	172.53 (−24.69 to 372.09)	157.02 (−22.14 to 328.22)	−0.23 (−0.38 to −0.08)
Low-middle	67,430.68 (−10,272.50 to 143,820.57)	169,233.02 (−24,369.13 to 361,478.97)	12.64 (−1.93 to 26.76)	13.54 (−1.96 to 28.81)	0.38 (0.27–0.49)	1,785,423.50 (−270,991.78 to 3,878,566.49)	3,874,157.61 (−552,190.38 to 8,296,861.59)	285.91 (−43.55 to 614.67)	275.71 (−39.54 to 589.56)	0.02 (−0.09 to 0.13)
Low	21,905.09 (−3,345.52 to 45,754.32)	48,882.29 (−7,143.94 to 102,006.90)	11.55 (−1.79 to 23.92)	12.23 (−1.80 to 25.30)	0.33 (0.19–0.47)	570,732.79 (−85,914.93 to 1,206,635.03)	1,143,287.10 (−165,426.56 to 2,416,512.17)	253.44 (−38.60 to 532.62)	239.69 (−35.02 to 502.18)	−0.14 (−0.25 to −0.02)
**Regions**										
Andean Latin America	877.32 (−118.59 to 1,973.47)	1,939.80 (−262.69 to 4,305.54)	4.83 (−0.65 to 10.81)	3.46 (−0.47 to 7.67)	−1.28 (−1.58 to −0.98)	18,954.46 (−2,498.88 to 42,348.02)	36,478.68 (−4,908.44 to 81,565.85)	94.79 (−12.62 to 212.47)	62.94 (−8.50 to 140.41)	−1.51 (−1.80 to −1.22)
Australasia	2,309.06 (−324.58 to 5,046.00)	1,636.42 (−234.29 to 3,537.21)	10.15 (−1.43 to 22.15)	2.60 (−0.37 to 5.64)	−4.61 (−4.73 to −4.49)	43,637.49 (−6,122.71 to 95,018.01)	23,768.66 (−3,387.75 to 51,395.65)	187.73 (−26.31 to 408.89)	41.29 (−5.85 to 89.29)	−5.09 (−5.19 to −5.00)
Caribbean	3,486.88 (−518.96 to 7,506.10)	5,397.47 (−769.39 to 11,485.44)	14.62 (−2.17 to 31.49)	9.88 (−1.41 to 21.02)	−1.26 (−1.36 to −1.17)	74,607.35 (−11,139.70 to 160,139.68)	106,836.78 (−15,056.87 to 232,685.48)	293.34 (−43.78 to 629.73)	197.46 (−27.83 to 430.53)	−1.25 (−1.35 to −1.15)
Central Asia	4,513.09 (−623.69 to 9,788.19)	6,711.50 (−945.21 to 14,489.79)	11.02 (−1.52 to 23.98)	10.51 (−1.49 to 22.80)	−0.50 (−0.86 to −0.12)	94,131.77 (−13,028.77 to 203,624.41)	129,488.79 (−18,083.13 to 278,052.24)	210.33 (−29.11 to 455.51)	179.01 (−25.16 to 385.57)	−0.97 (−1.40 to −0.54)
Central Europe	13,041.62 (−1,818.92 to 27,759.61)	13,136.86 (−1,868.44 to 28,689.74)	9.63 (−1.34 to 20.62)	5.49 (−0.78 to 11.99)	−2.14 (−2.29 to −1.99)	275,310.78 (−38,498.58 to 586,644.66)	212,874.79 (−30,224.35 to 459,749.30)	190.21 (−26.57 to 404.72)	92.98 (−13.17 to 200.62)	−2.68 (−2.84 to −2.52)
Central Latin America	7,423.91 (−1,049.02 to 15,601.93)	20,488.73 (−2,979.88 to 44,940.91)	10.64 (−1.51 to 22.48)	8.71 (−1.27 to 19.11)	−0.80 (−0.96 to −0.65)	160,321.87 (−22,449.31 to 337,278.05)	369,802.33 (−53,224.75 to 804,469.10)	202.14 (−28.51 to 423.79)	151.51 (−21.87 to 329.58)	−1.14 (−1.29 to −1.00)
Central Sub-Saharan Africa	1,362.20 (−191.15 to 2,868.14)	3,185.53 (−469.13 to 6,815.66)	7.71 (−1.10 to 16.21)	7.98 (−1.16 to 17.16)	−0.03 (−0.09 to 0.03)	35,613.84 (−4,986.25 to 74,965.29)	77,868.81 (−11,612.26 to 165,336.52)	163.64 (−22.97 to 342.78)	156.05 (−22.92 to 334.88)	−0.29 (−0.36 to −0.23)
East Asia	39,209.02 (−5,554.94 to 84,029.33)	154,244.52 (−21,706.36 to 332,276.80)	6.22 (−0.90 to 13.52)	8.36 (−1.18 to 17.97)	1.39 (0.99–1.79)	955,503.88 (−132,517.00 to 2,058,801.92)	2,653,373.10 (−369,088.74 to 5,784,456.12)	119.42 (−16.93 to 255.28)	131.24 (−18.33 to 286.28)	0.67 (0.32–1.01)
Eastern Europe	17,160.99 (−2,305.99 to 37,247.49)	23,972.18 (−3,050.28 to 52,146.53)	6.97 (−0.94 to 15.18)	6.66 (−0.85 to 14.50)	−0.59 (−1.18 to −0.01)	352,876.43 (−47,231.18 to 760,937.20)	425,215.95 (−53,263.68 to 938,292.42)	132.82 (−17.78 to 286.75)	120.10 (−15.03 to 265.20)	−0.90 (−1.56 to −0.23)
Eastern Sub-Saharan Africa	3,501.75 (−520.38 to 7,336.42)	7,087.23 (−955.51 to 15,096.33)	5.75 (−0.87 to 11.91)	5.55 (−0.75 to 11.71)	−0.31 (−0.49 to −0.13)	88,864.91 (−12,938.94 to 189,685.88)	165,384.85 (−22,320.92 to 351,561.31)	122.88 (−18.21 to 258.82)	106.96 (−14.53 to 227.42)	−0.66 (−0.85 to −0.48)
High-income Asia Pacific	3,872.15 (−536.89 to 8,463.65)	5,140.06 (−725.95 to 11,489.23)	2.18 (−0.30 to 4.78)	0.81 (−0.11 to 1.79)	−3.16 (−3.24 to −3.07)	73,845.60 (−10,155.35 to 160,384.36)	72,226.42 (−10,189.84 to 159,243.26)	38.40 (−5.29 to 83.71)	13.88 (−1.95 to 30.60)	−3.31 (−3.37 to −3.25)
High-income North America	23,759.74 (−3,412.46 to 51,693.89)	17,021.62 (−2,389.80 to 37,150.48)	6.53 (−0.94 to 14.20)	2.35 (−0.33 to 5.12)	−3.60 (−3.75 to −3.46)	425,209.74 (−60,567.55 to 919,724.93)	273,647.41 (−38,082.09 to 589,744.64)	120.90 (−17.16 to 261.61)	40.19 (−5.57 to 86.36)	−3.84 (−3.99 to −3.70)
North Africa and Middle East	34,784.53 (−5,184.76 to 74,837.30)	66,487.56 (−9,522.05 to 143,314.38)	24.69 (−3.69 to 53.31)	18.16 (−2.63 to 39.14)	−1.05 (−1.13 to −0.96)	858,565.32 (−127,181.20 to 1,840,439.73)	1,434,262.49 (−202,240.29 to 3,105,384.99)	515.95 (−76.83 to 1,109.53)	335.84 (−47.91 to 725.26)	−1.46 (−1.57 to −1.35)
Oceania	114.25 (−16.32 to 254.26)	279.51 (−37.06 to 618.28)	5.16 (−0.73 to 11.34)	4.95 (−0.67 to 10.92)	−0.13 (−0.25 to −0.00)	3,077.71 (−428.97 to 6,808.63)	7,062.43 (−921.70 to 15,821.35)	107.75 (−15.44 to 239.73)	98.86 (−13.12 to 218.90)	−0.26 (−0.40 to −0.11)
South Asia	64,718.08 (−9,936.13 to 138,448.20)	184,303.85 (−26,476.60 to 388,248.01)	12.64 (−1.95 to 26.67)	14.30 (−2.06 to 29.94)	0.58 (0.43 to 0.74)	1,783,337.36 (−271,122.82 to 3,853,627.75)	4,254,075.09 (−605,200.26 to 9,047,992.99)	296.10 (−45.34 to 633.39)	293.64 (−42.00 to 622.01)	0.10 (−0.02 to 0.22)
Southeast Asia	12,590.19 (−1,922.78 to 27,065.59)	33,548.35 (−4,808.29 to 71,767.26)	5.56 (−0.86 to 11.87)	5.90 (−0.85 to 12.55)	0.20 (0.01–0.39)	341,089.25 (−51,040.51 to 737,769.27)	779,601.92 (−110,929.77 to 1,686,169.20)	127.89 (−19.41 to 276.33)	120.62 (−17.25 to 259.56)	−0.18 (−0.35 to −0.00)
Southern Latin America	1,834.00 (−248.22 to 4,098.40)	1,742.71 (−243.89 to 3,878.95)	4.27 (−0.58 to 9.55)	1.92 (−0.27 to 4.27)	−2.29 (−2.38 to −2.20)	38,948.59 (−5,279.22 to 86,268.16)	31,898.38 (−4,453.64 to 70,213.13)	85.84 (−11.63 to 190.41)	36.24 (−5.05 to 79.82)	−2.57 (−2.64 to −2.50)
Southern Sub-Saharan Africa	891.54 (−120.93 to 1,957.72)	2,070.74 (−288.64 to 4,496.92)	3.73 (−0.51 to 8.19)	4.36 (−0.61 to 9.47)	0.49 (−0.03 to 1.01)	22,446.03 (−3,069.02 to 49,405.21)	47,597.56 (−6,644.63 to 102,435.02)	81.87 (−11.19 to 179.54)	86.09 (−12.05 to 185.94)	0.14 (−0.40 to 0.68)
Tropical Latin America	6,800.36 (−948.84 to 14,597.03)	9,898.68 (−1,448.41 to 21,306.64)	8.60 (−1.21 to 18.51)	3.97 (−0.58 to 8.54)	−2.32 (−2.39 to −2.25)	165,880.56 (−23,009.86 to 355,595.52)	209,467.96 (−30,528.60 to 449,535.06)	182.35 (−25.44 to 390.62)	81.53 (−11.90 to 175.13)	−2.49 (−2.58 to −2.41)
Western Europe	30,816.21 (−4,343.98 to 66,303.79)	21,234.21 (−2,972.93 to 46,930.25)	5.20 (−0.73 to 11.20)	1.79 (−0.25 to 3.94)	−3.67 (−3.84 to −3.49)	565,100.69 (−79,350.95 to 1,207,274.34)	304,533.68 (−42,615.94 to 669,076.93)	98.17 (−13.75 to 210.05)	29.37 (−4.08 to 64.63)	−4.12 (−4.28 to −3.96)
Western Sub-Saharan Africa	4,615.06 (−665.71 to 9,875.55)	10,842.50 (−1,545.45 to 23,308.15)	6.39 (−0.92 to 13.57)	7.35 (−1.05 to 15.71)	0.45 (0.23–0.67)	107,080.32 (−15,419.79 to 229,558.99)	239,145.35 (−33,805.28 to 523,082.71)	127.96 (−18.48 to 273.00)	135.55 (−19.37 to 292.71)	0.17 (−0.07 to 0.40)

**Figure 1:**
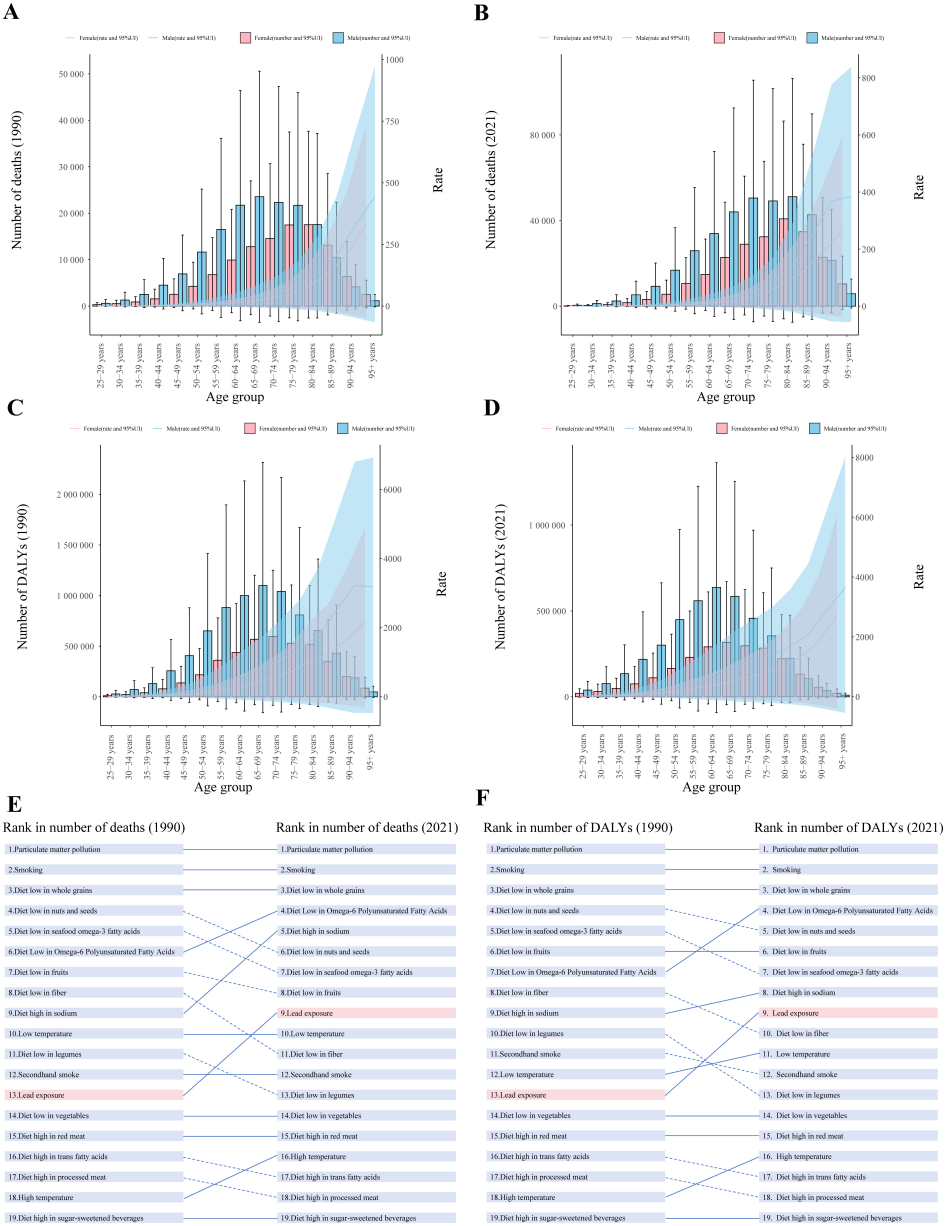
The global burden of IHD due to lead exposure in 1990 and 2021. **(A, B)** Sex-specific death and DALYs number for different age groups, 1990 and 2021. **(C, D)** Sex-specific mortality and DALYs rates for different age groups, 1990 and 2021. **(E, F)** Ranking of the burden of IHD due to different risk factors in 1990 and 2021. Error bars and shadow bands indicate 95% uncertainty interval. IHD: ischemic heart disease; DALYs: disability-adjusted life years. Risk factors are connected by lines between time periods, where solid lines represent an increase or no change in rank and dashed lines represent a decrease in rank.

In both 1990 and 2021, the number of deaths and DALYs and ASRs were higher for males than for females (Table 1). Males consistently exhibited higher death and DALYs number across all age groups compared to females, with the exception of individuals aged 85 years and older (Figures 1A–D). In all age groups, males had higher rates of mortality and DALYs than females (Figures 1A–D). Trends by sex and age group: trends in rates of mortality and DALYs over time were not consistent across age groups for males, but there was an overall decreasing trend; rates for females were decreasing across age groups, consistent with the overall trend (Table 1, Supplementary Figure S1). Males had a higher burden of IHD than females.

From 1990 to 2021, countries with low and low-middle SDI consistently had the highest ASMR and ASDR while high SDI countries had the lowest (Supplementary Figure S2). A significant decline in ASMR and ASDR was observed in high SDI countries, with the most significant decline recorded (EAPC for ASMR: −3.43, 95% CI: −3.53 to −3.34; EAPC for ASDR: −3.56, 95% CI: −3.64 to −3.48). In contrast, ASMR increased in middle, low-middle, and low SDI countries (Table 1, Supplementary Figure S2).

**Figure 2:**
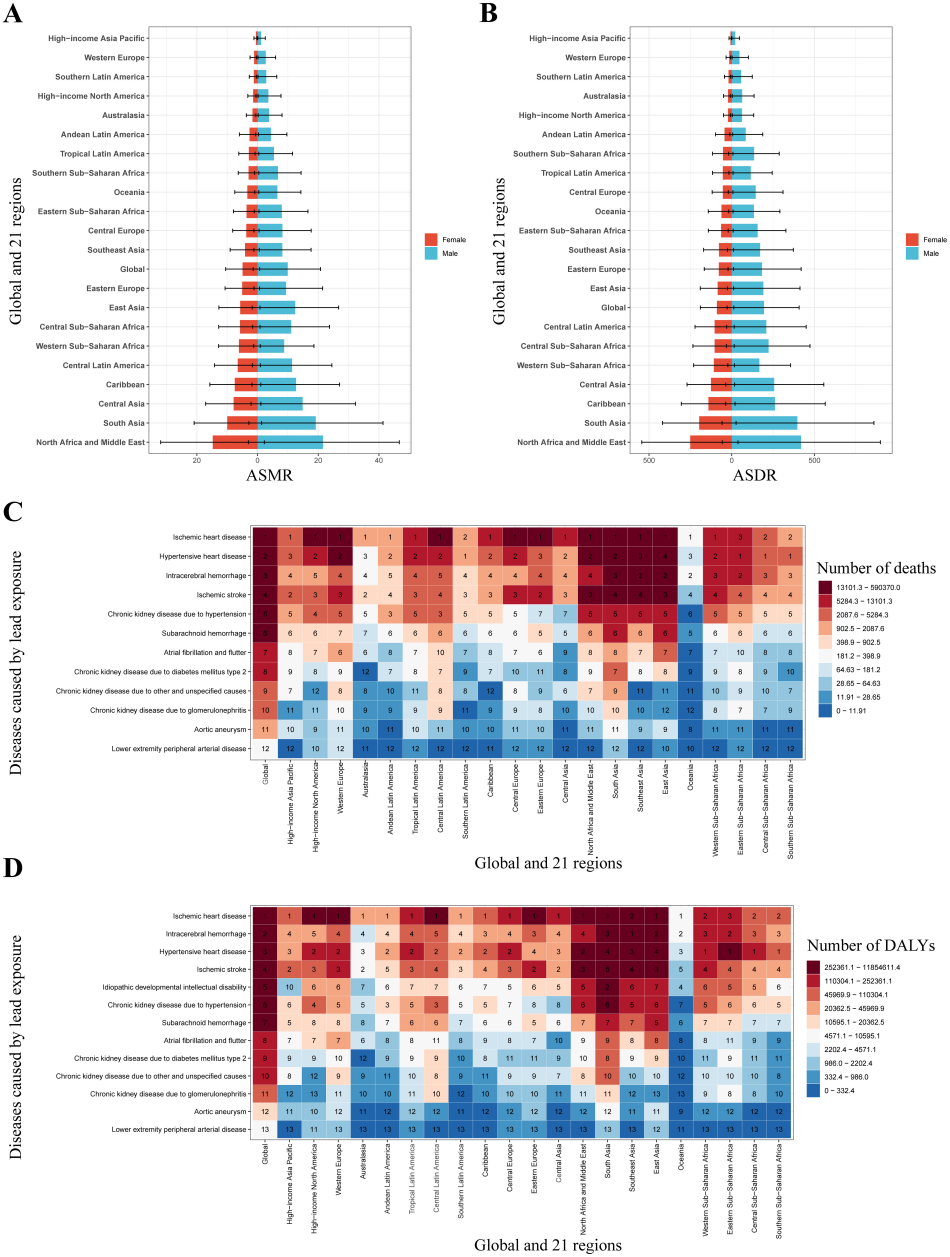
Regional level burden of lead exposure IHD: sex-specific disparities and multiple disease comparative risk assessment (2021). **(A)** The ASMR of IHD due to lead exposure by sex at the regional level in 2021. **(B)** The ASMR of IHD due to lead exposure by sex at the regional level in 2021. **(C)** Ranking of the deaths number from 13 diseases caused by lead exposure. **(D)** Ranking of the DALYs number from 13 diseases caused by lead exposure. Error bars indicate 95% uncertainty intervals. IHD: ischemic heart disease; ASMR: age-standardized mortality rate; ASDR: age-standardized disability-adjusted life years rate.

In addition, among all risk factors for IHD included in GBD 2021, both the number of deaths and DALYs attributable to lead exposure increased from 13th in 1990 to 9th in 2021 (Figures 1E, F). Among the 13 diseases attributable to lead exposure in 2021, IHD accounted for the highest number of deaths and DALYs globally (Figures 2C, D).

### 3.2 Regional level trends

In 2021, South Asia recorded the highest number of deaths and DALYs due to IHD attributable to lead exposure, while the highest ASRs were observed in North Africa and Middle East (Table 1, Figures 2A, B). From 1990 to 2021, ASRs decreased in most regions, with a sharp decrease in Australasia, but a sharp increase in East Asia (Table 1). Across all 21 regions, males consistently exhibited higher ASRs than females (Figures 2A, B). In all regions except Sub-Saharan Africa, IHD accounted for the highest number of deaths and DALYs among the 13 diseases attributable to lead exposure (Figures 2C, D).

**Figure 3:**
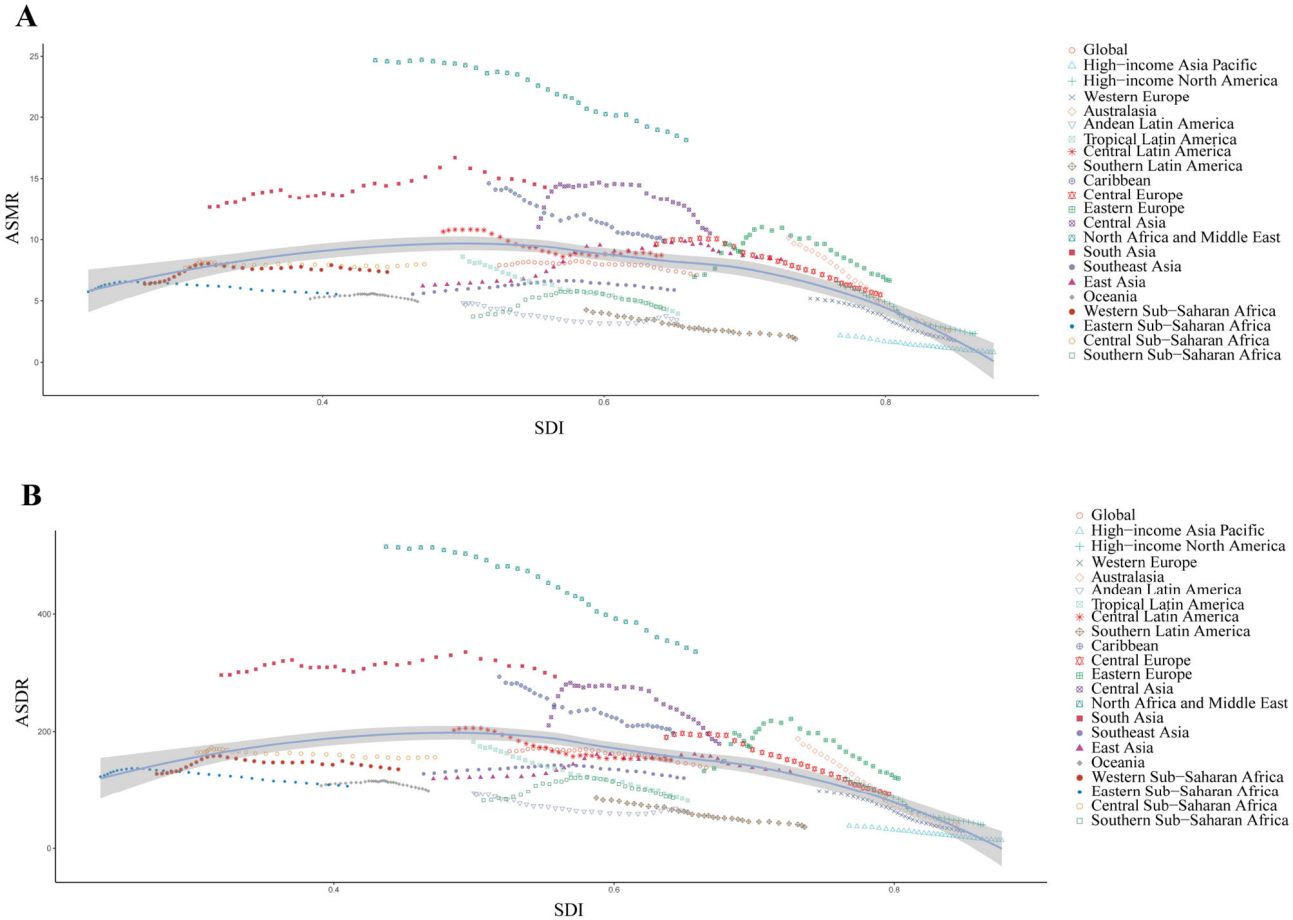
Correlation between SDI and ASMR/ASDR of IHD due to lead exposure at the global and regional level from 1990 to 2021. **(A)** Correlation between SDI and ASMR, *r* = −0.3637, *P* < 0.001, df = 670. **(B)** Correlation between SDI and ASDR, *r* = −0.4389, *P* < 0.001, df = 670. Colored lines show global and regional values for ASRs, and each point in a line represents 1 year. Expected values based on the SDI and ASRs in all locations are shown as the line. SDI: socio-demographic index; IHD: ischemic heart disease; ASMR: age-standardized mortality rate; ASDR: age-standardized disability-adjusted life years rate.

The SDI for IHD due to lead exposure was inversely correlated with the estimated relationship between expected ASMR (*r* = −0.3637, *P* < 0.001, df = 670) and ASDR (*r* = −0.4389, *P* < 0.001, df = 670) from 1990 to 2021 (Figure 3). The negative correlation between SDI and ASRs suggested that socio-economic development was critical for reducing lead exposure-related IHD burden. North Africa and the Middle East, along with South Asia, exhibited significantly higher-than-expected ASMR values, whereas other regions showed ASMR values that were either lower or in line with expectations (Figure 3A). A similar trend was observed for ASDR, with a comparable relationship between SDI and ASDR (Figure 3B).

### 3.3 National level trends

**Figure 4:**
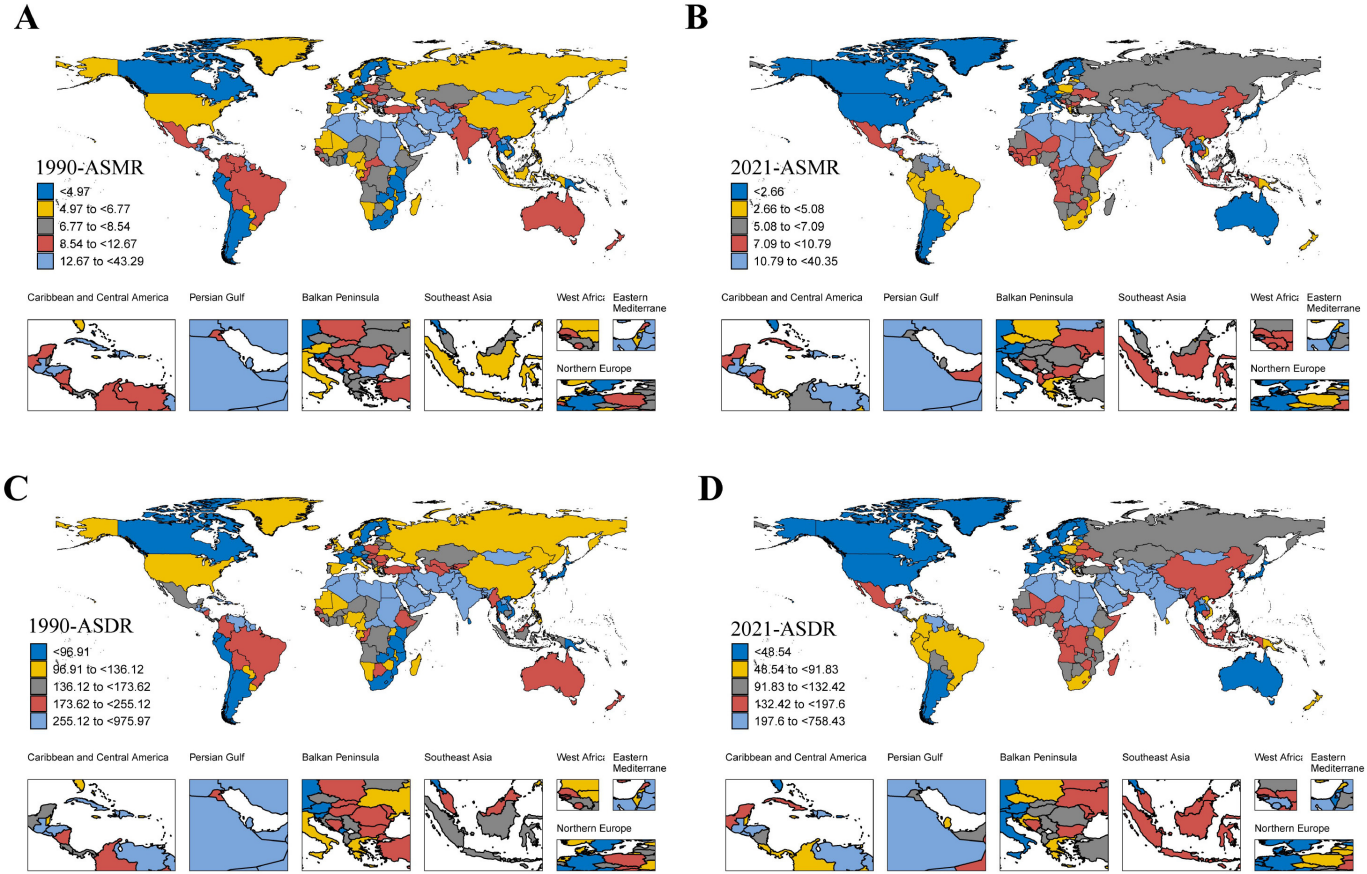
The ASMR/ASDR of IHD due to lead exposure at the national level in 1990 and 2021. **(A, B)** The ASMR in 1990 and 2021. **(C, D)** The ASDR in 1990 and 2021. IHD: ischemic heart disease; ASMR: age-standardized mortality rate; ASDR: age-standardized disability-adjusted life years rate.

At the national level, the ASRs of IHD due to lead exposure declined in most countries in 2021 compared to 1990, with the largest decline occurred in Israel (Supplementary Tables S1, S2, Supplementary Figure S3). In 2021, China, India, and Pakistan had the highest number of deaths and DALYs due to lead-related IHD, while Egypt, Afghanistan, and Yemen recorded the highest ASRs (Supplementary Table S3, Figure 4). From 1990 to 2021, an increasing trend in ASMR was observed in 50 countries, while 36 countries showed an increasing trend in ASDR (Supplementary Tables S1, S2).

**Figure 5:**
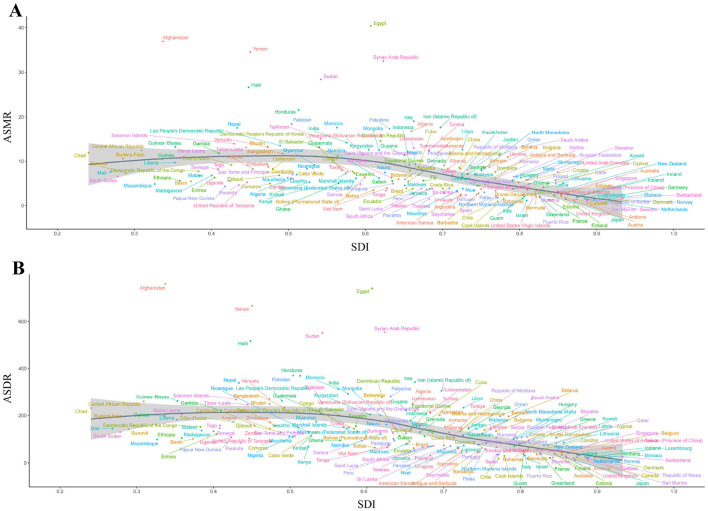
Correlation between SDI and ASMR/ASDR of IHD due to lead exposure at the national level in 2021 **(A)** Correlation between SDI and ASMR, *r* = −0.6443, *P* < 0.001, df = 6,526. **(B)** Correlation between SDI and ASDR, *r* = −0.6910, *P* < 0.001, df = 6,526. Each colored dot above represents a country, and the line represents the average expected value based on the SDI and ASRs in 204 countries and territories. SDI: socio-demographic index; ASRs, age-standardized rates; IHD: ischemic heart disease; ASMR: age-standardized mortality rate; ASDR: age-standardized disability-adjusted life years rate.

**Figure 6:**
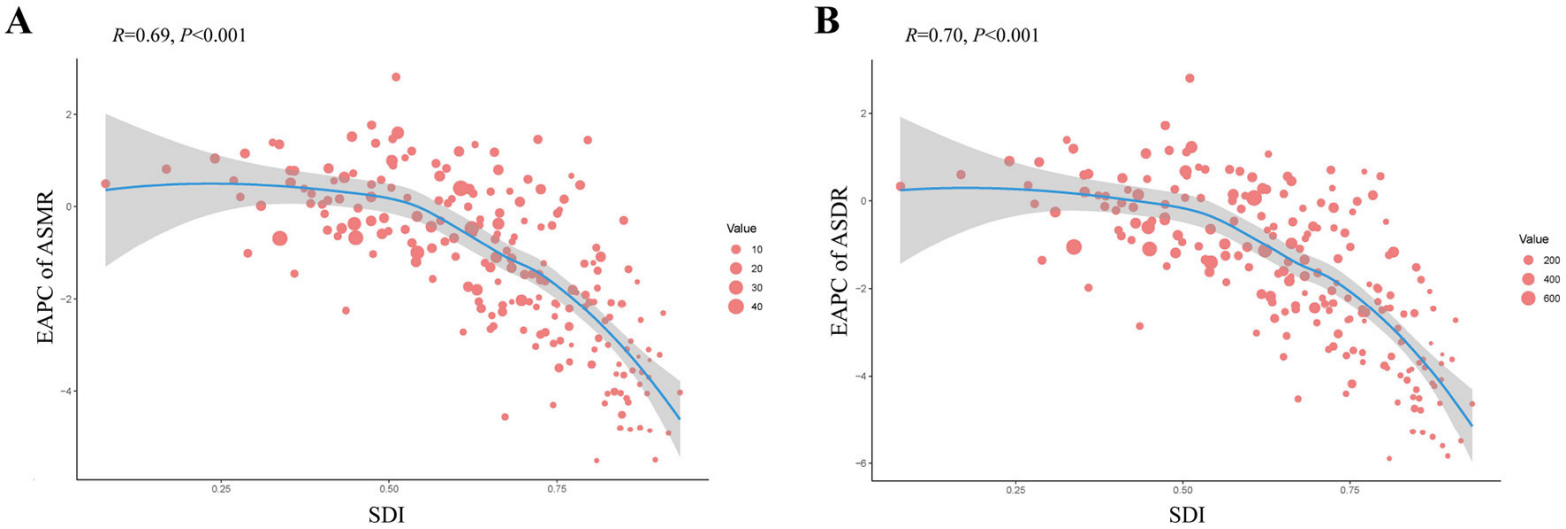
Correlation between the EAPC of ASMR/ASDR and SDI of IHD due to lead exposure at the national level in 2021. **(A)** Correlation between the EAPC of ASMR and SDI. **(B)** Correlation between the EAPC of ASDR and SDI. Each red dot above represents a country, and the line represents the average expected value based on the EAPC and SDI in 204 countries and territories. The size of the red dots represents the ASRs of the IHD. SDI: socio-demographic index; IHD: ischemic heart disease; EAPC: estimated annual percentage change; ASRs, age-standardized rates; ASMR: age-standardized mortality rate; ASDR: age-standardized disability-adjusted life years rate.

A negative correlation was identified between ASRs and the SDI for IHD caused by lead exposure across 204 countries in 2021 (Figure 5). The negative correlation between SDI and ASRs suggested that socio-economic development was critical for reducing lead exposure-related IHD burden at the national level. Based on the SDI, the ASRs in Egypt, Afghanistan, Yemen, Syrian Arab Republic, Sudan, and Haiti were substantially higher than expected, whereas ASRs in the remaining countries were either lower than or comparable to expectations (Figure 5). In addition, the EAPC in ASRs was found to be negatively correlated with SDI (Figure 6).

### 3.4 Decomposition analysis

**Figure 7:**
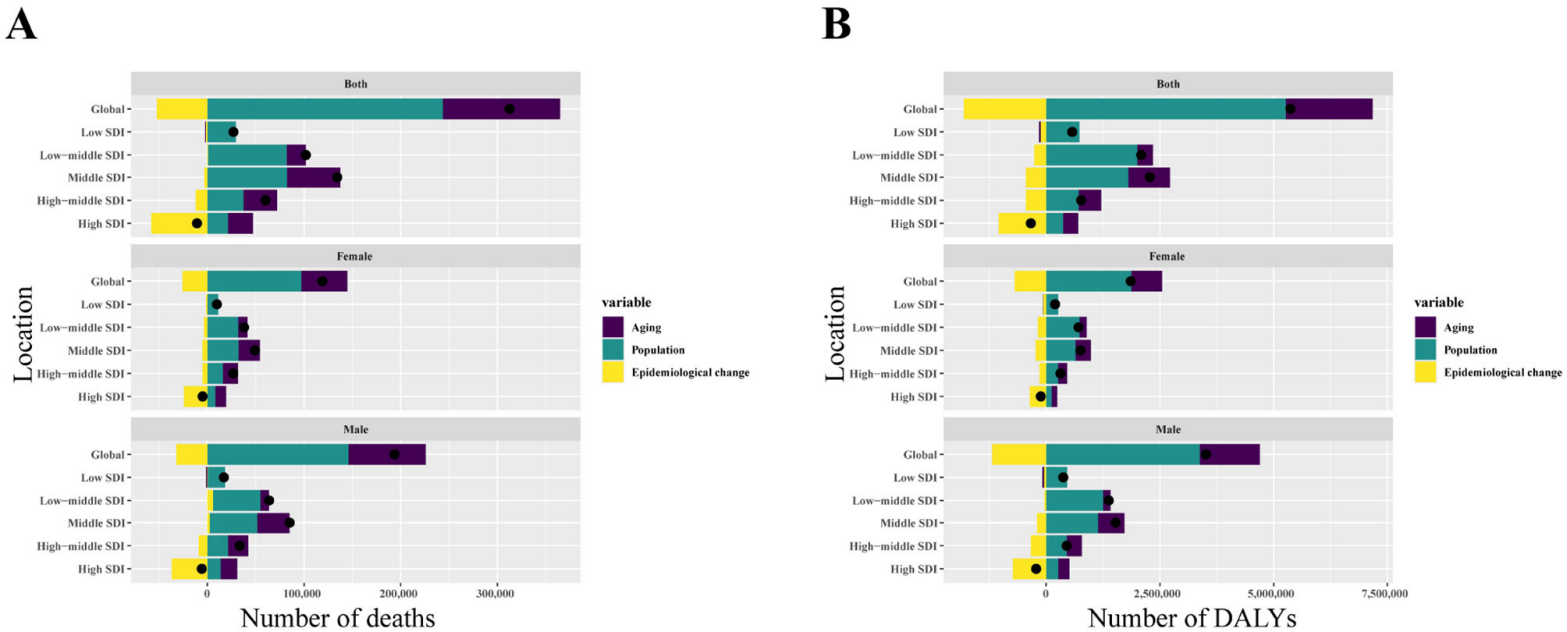
Changes in IHD due to exposure of deaths and DALYs numbers according to population growth, aging, and epidemiological change from 1990 to 2021 at the global level and by SDI quintile. **(A)** The driving factors of deaths number. **(B)** The driving factors of DALYs number. The black dot represents the overall value of change contributed by all three components. For each component, the magnitude of a positive value indicates a corresponding increase in IHD due to lead exposure of death and DALYs attributed to the component; the magnitude of a negative value indicates a corresponding decrease in IHD due to lead exposure of death and DALYs attributed to the related component. SDI: socio-demographic index; IHD: ischemic heart disease; DALYs: disability-adjusted life years.

Over the past 32 years, the global number of deaths and DALYs from IHD due to lead exposure has increased (Figure 7). Population growth emerged as the primary driver of this increase, accounting for 77.94% of the global rise in death numbers, followed by aging, which contributed 38.81%. In contrast, the effect of epidemiologic changes on the global increase in death number was negative (−16.75%; Figure 7A, Supplementary Table S4). A similar pattern was observed in the global increase in DALYs, with population growth playing key roles (Figure 7B, Supplementary Table S5).

Notably, the increase in IHD burden was more pronounced in males than in females, primarily driven by population growth, indicating a significant gender disparity (Figure 7). Of the five SDI regions, the middle SDI region experienced the largest increase in both deaths and DALYs, while the high SDI region saw a decline in the burden (Figure 7).

### 3.5 Potential for burden reduction: frontier analysis

**Figure 8:**
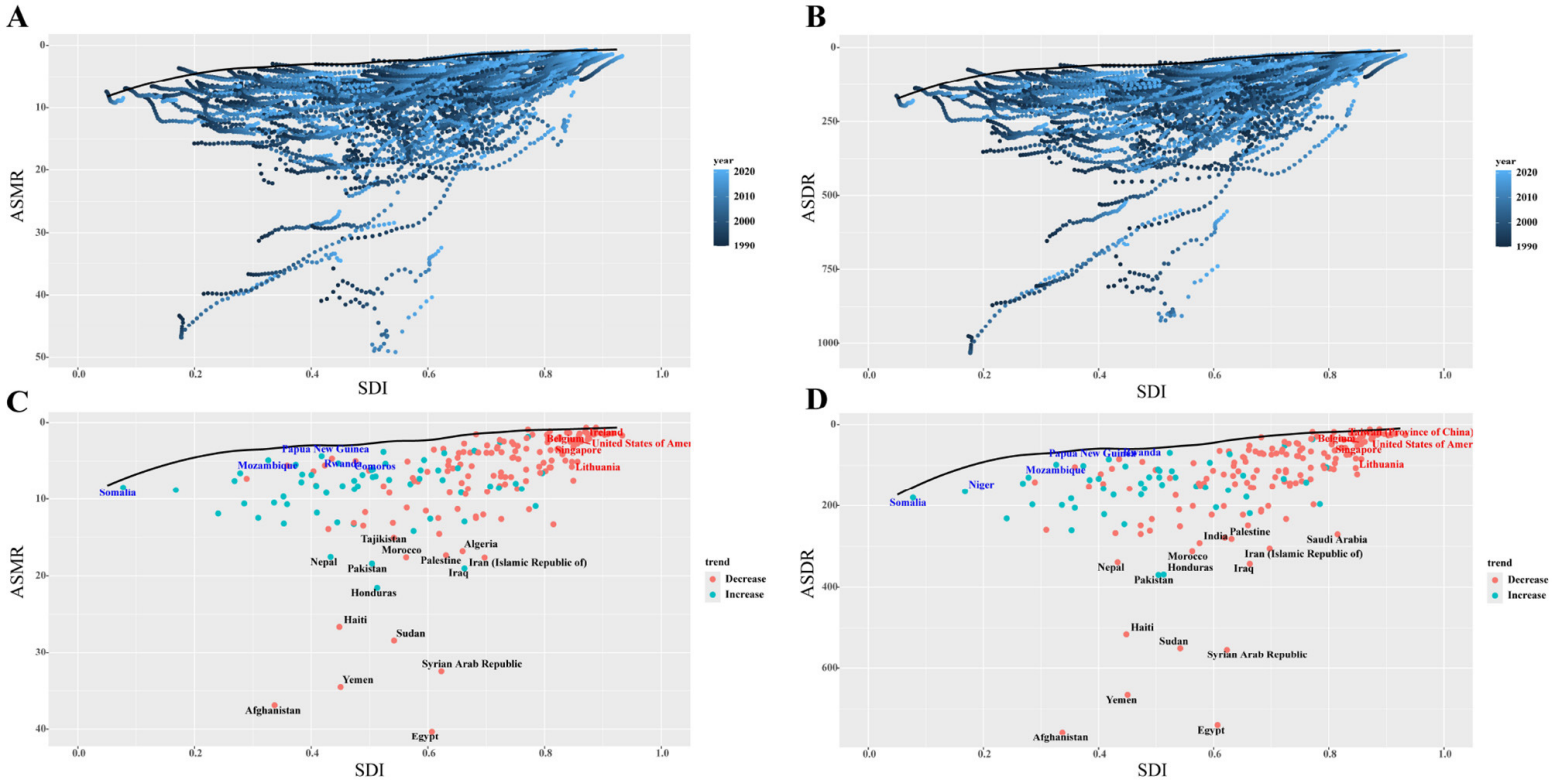
Frontier analysis of SDI-based ASMR and ASDR for IHD in 2021. **(A, B)** The frontier values of ASMR and ASDR from 1990–2021 for all 204 countries with the SDI. **(C, D)** The frontier values in 2021 for all 204 countries. The red dots indicated that the rate in 2021 is higher than 1990, while the blue dots indicate that 2021 is lower than 1990. The dots marked in black are the 10 countries with the largest gap between the frontier and the actual value, the dots marked in red are the five countries with the largest gap between the frontier and the actual value for countries above the high SDI threshold, and the dots marked in blue are the five countries with the smallest gap between the frontier and the actual value for countries below the low SDI threshold. SDI: socio-demographic index; IHD: ischemic heart disease; ASMR: age-standardized mortality rate; ASDR: age-standardized disability-adjusted life years rate.

The frontier analysis of ASMR and ASDR for lead exposure, based on the SDI, is shown in Figure 8, Supplementary Tables S6, S7. Unrealized health benefits shrank with increasing SDI in most countries or areas from 1990 to 2021 (Figures 8A, B). Among the 204 countries, Egypt had the largest disparity between frontier deaths and actual mortality values (Figure 8C, Supplementary Table S6), while Afghanistan had the largest difference between frontier DALYs and actual values (Figure 8D, Supplementary Table S7). The analysis indicated that as SDI improved, the difference between actual and frontier values generally narrowed, suggesting that countries or regions with middle and low SDI rankings have the most potential for reducing the disease burden (Figures 8C, D).

### 3.6 Projection trends of IHD in 2021–2050

**Figure 9:**
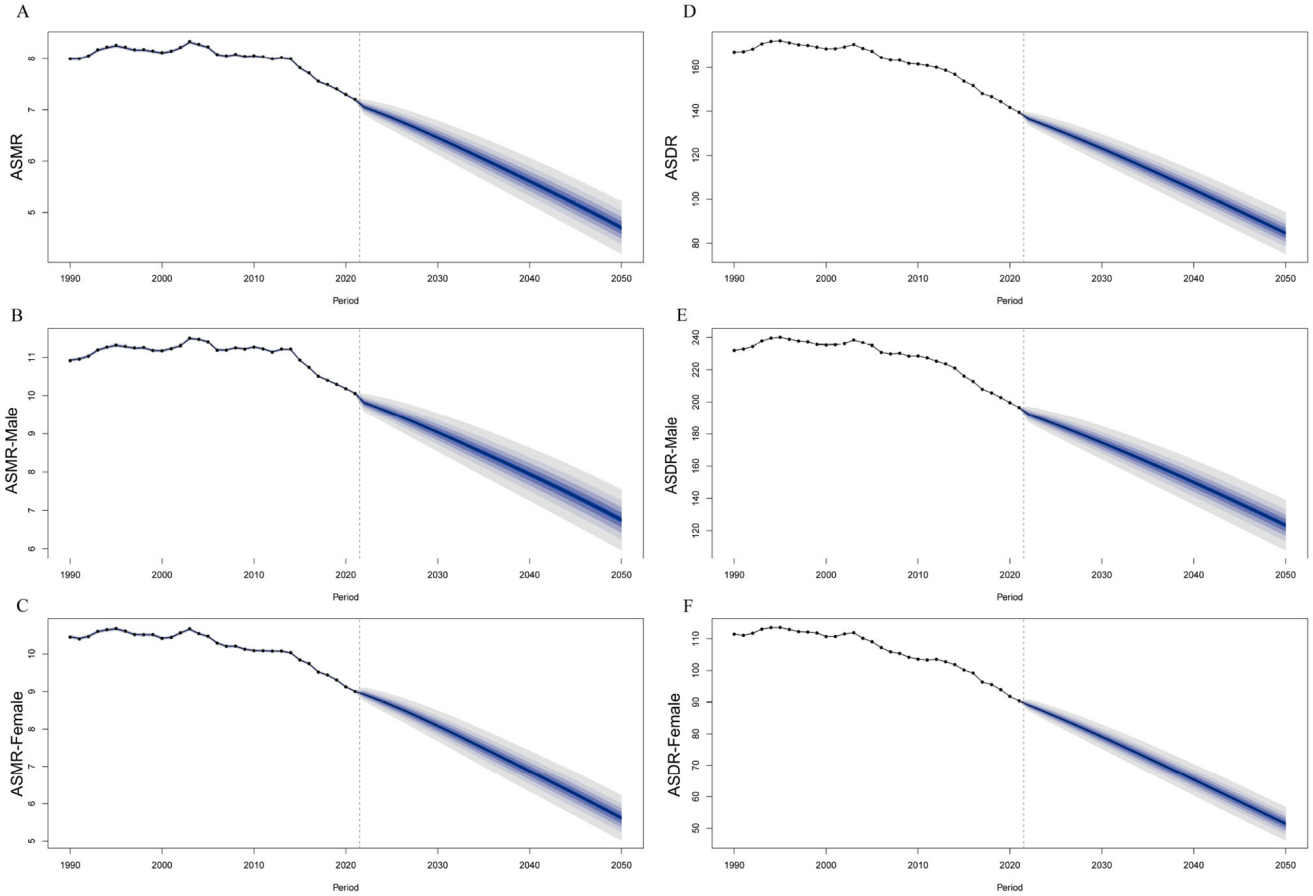
The projection of ASMR/ASDR of IHD due to lead exposure globally from 2021 to 2050. **(A–C)** The projection of ASMR and sex-specific ASMR. **(D–F)** The projection of ASDR and sex-specific ASDR. IHD: ischemic heart disease; ASRs: age-standardized rates; ASMR: age-standardized mortality rate; ASDR: age-standardized disability-adjusted life years rate.

To assess the future burden of IHD attributable to lead exposure, the BAPC model was used to project trends in ASRs for the next 29 years. The model predicts a decline in the ASRs of IHD due to lead exposure across all demographics, including overall populations, males, females, and various age groups (Figure 9, Supplementary Figure S4). By 2050, the ASMR and ASDR are projected to decline to 4.70 (95% CI: 4.10–5.31) and 84.63 (95% CI: 73.45–95.81) per 100,000 population, respectively.

## 4 Discussion

This study provides an updated analysis of the global, regional, and national burden of IHD attributable to lead exposure based on data from the GBD 2021. It also projects future trends in IHD burden from 2021 to 2050. The results of the study showed that, globally, the number of deaths and DALYs in IHD due to lead exposure increased from 1990 to 2021, while the ASRs decreased, which is consistent with the trend in overall IHD reported by GBD 2019 (6). Males, the older adult, and those living in low and low-middle SDI areas bear the highest burden of IHD from lead exposure. Although the burden of IHD due to lead exposure was trending downward in most regions and countries, it was still trending upward in about one-fifth of the countries. Decomposition analysis highlights that population growth is the dominant factor driving the increasing number of deaths and DALYs, while frontier analysis identifies middle and low SDI regions as having significant potential for reducing the disease burden. Projected trends to 2050 suggest that the global burden of IHD due to lead exposure will decline. These findings offer valuable insights into the global burden of IHD related to lead exposure and underscore the need for targeted interventions to reduce exposure in vulnerable populations.

The global decline in IHD burden attributable to lead exposure by 2021 compared to 1990 may be attributed to international regulations aimed at controlling lead exposure. The Model Law and Guidance for Regulating Lead Paint, published by the United Nations Environment Programme in 2017, has been instrumental in prompting countries to implement stricter lead regulations (30). Successful initiatives, such as Flint, Michigan's effort to replace lead service lines, have shown that regulating lead exposure can significantly reduce public health risks (31). Similarly, European countries have made strides in reducing lead exposure through monitoring programs that track lead concentrations and implement measures to mitigate emissions (32). Furthermore, in addition to lead exposure, environmental risk factors for IHD included particulate matter pollution, low temperatures, and high temperatures (33). The burden of IHD due to particulate matter pollution was higher than lead exposure (34). However, the burden of IHD due to lead exposure is higher compared to low and high temperatures (35). This study showed that both the number of deaths and DALYs attributable to lead exposure increased from 13th in 1990 to 9th in 2021. Previous studies showed that lead exposure was associated with the development of 12 diseases in addition to IHD (e.g., stroke, hypertensive heart disease, and diabetes and kidney disease, among others), but the burden was highest for IHD (15). Thus, the burden of IHD due to lead exposure remained a serious situation.

This study also found a pronounced gender disparity, with males experiencing a higher burden of IHD due to lead exposure than females. This gender difference might have been related to occupational factors, lifestyle, and sex hormones. Firstly, males were more likely to work in occupations with a higher risk of lead exposure, such as telecommunications workers, plumbers, and construction workers (36). Secondly, males were more likely to engage in lifestyles that increased the risk of IHD, including smoking and drinking alcohol (37, 38). Thirdly, sex hormones were associated with the development of ischemic heart disease (IHD), as estrogen played a key cardioprotective role; females demonstrated greater protection against cardiac damage compared to males, resulting in a lower incidence of IHD (39). Hence, males had a greater burden of disease due to higher levels of lead exposure, more prevalent smoking and drinking behaviors, and gender differences in the physiological role of sex hormones. Age also plays a critical role, with older adults bearing a higher burden of IHD. As the global population ages, IHD remains the leading cause of disability and death among the older adult (40). Younger individuals' hearts may better manage ischemic injury, as shown in study investigating the response of pyruvate dehydrogenase kinase 4 in cardiomyocytes (41). In addition, a prospective cohort study of lead exposure and IHD showed that the IHD group was older than the non-IHD group (42). Therefore, the prevention and treatment of IHD due to lead exposure should focus on key populations with targeted measures.

Regional disparities in the burden of IHD from lead exposure were observed, with South Asia, North Africa, and the Middle East having the highest burden, and East Asia exhibiting the most significant increase in disease burden. The continued use of lead paint in many countries, despite international efforts to phase it out, likely contributes to this issue (43). Data from GBD 2019 showed that the highest burden of disease due to lead exposure in North Africa and the Middle East was IHD, aligning with our findings (44). In addition, East Asia is aging at a faster rate than the rest of the world, which may be related to the increasing disease burden (45). At the national level, the decline in ASRs was most pronounced in Israel. This phenomenon might have been attributable to Israel's systematic monitoring of quality metrics for basic cardiovascular care, which was rigorously implemented by the Health Maintenance Organization and the Department of Medical Quality under the Ministry of Health, ensuring sustained efficacy in cardiovascular health maintenance (46). China, India, and Pakistan recorded the highest numbers of deaths and DALYs from IHD due to lead exposure, while Egypt, Afghanistan, and Yemen had the highest ASRs. These countries, all classified as developing nations, face unique challenges. China, India, and Pakistan, which rank among the top five most populous countries globally, have large populations and strained healthcare systems, contributing to a higher burden of deaths and DALYs (47, 48). Egypt, the most populous country in the Middle East and North Africa (49), relies heavily on the Nile River as its main water source. However, studies have found lead contamination in both the water and sediments of the Nile, increasing the risk of lead exposure through multiple pathways (50). Afghanistan has been in a state of war for a long time and health services have been severely affected, with inadequate coverage of primary health services (51). A study found that cooking utensils are a source of lead exposure in Afghanistan and that traditional Afghan pressure cookers contain high levels of lead above the Interim Reference Level for adults and children, increasing the risk of lead exposure in the Afghan population (52). Furthermore, as a result of the conflict in Yemen, there is fragmentation of the health system, which is under great pressure and has a high burden of disease, thus hindering the implementation of universal health coverage and health promotion (53). These examples demonstrate that despite global improvements, the burden of IHD due to lead exposure remains disproportionately high in developing countries, highlighting the need for proactive measures and interventions to reduce lead exposure.

The burden of IHD due to lead exposure was found to be closely linked with the SDI, showing a negative correlation. The highest burden was observed in low and low-middle SDI regions, while high SDI regions experienced the lowest burden. These findings are consistent with overall CVD patterns reported in GBD 2019 (54). The differences in the different SDI may be due to the earlier implementation of lead exposure control measures in developed countries. In 1985, the European Union required all member states to use unleaded petrol from October 1989, which reduced lead emissions to some extent (55). In contrast, until 2002, low and middle income countries continued to use leaded gasoline, resulting in persistently high levels of exposure (56). Lead in paint is also a significant source of lead exposure, and the latest report shows that 31 countries in the European region now do not allow the use of lead in paint, while lower-middle-income countries, such as Brazil, Mexico and Oman, still allow higher lead limits (600 mg/kg) (57). It follows that areas with lower SDI often have difficulty enforcing strict regulations and have limited control over lead exposure, resulting in a higher burden of disease. In addition to regulatory differences, there is an unequal distribution of healthcare resources and unbalanced access to healthcare in different socio-economic situations, resulting in different burdens of IHD due to lead exposure (58, 59). Developed countries have better socio-economic, nutritional, and medical resources than developing countries (32). Moreover, the staggering volume of e-waste generated globally drives informal recycling operations in many low- and middle-income countries as a desperately needed income source. However, the prevailing unsafe disposal practices in under-resourced settings heighten susceptibility to lead contamination in these regions (60). The results of the decomposition analysis showed that population growth and aging were driving the increase in the number of deaths and DALYs globally, particularly in the Central SDI region. Global population projections suggest that the population will reach 9.7 billion by 2050, with most of the growth occurring in low- and middle-income countries (61, 62).

Frontier analysis showed that middle and low SDI regions have significant potential to reduce the burden of IHD. To address this, countries in these regions should implement policies to control lead exposure by targeting its sources, enhancing risk assessment, and improving primary healthcare. Additionally, rational allocation of healthcare resources is essential to ensure that individuals with IHD can access appropriate care.

This study has several limitations. First, the bone lead levels were calculated on the basis of blood lead, rather than measured directly, and although lead exposure data were available from multiple sources, including 553 studies in 85 countries, actual blood lead measurements were lacking in certain regions, particularly in low and middle income countries (21). Second, lead exposure was a potential risk factor for a variety of cardiovascular diseases (e.g., stroke, rheumatic heart disease, hypertensive heart disease), and this study's exclusive focus on IHD and exclusion of other cardiovascular conditions might have resulted in an underestimation of lead's total health impact (15). Third, there were multiple risk factors for IHD (e.g., hypertension, particulate matter pollution, smoking, etc.), but only the burden of IHD due to lead exposure was analyzed in this study, which may lead to an underestimation of the burden of IHD.

## 5 Conclusions

In conclusion, the results suggest that the burden of IHD due to lead exposure has decreased in most regions and countries, especially in high SDI. However, the burden remains significant among males, older adults, and in low, middle, and low-middle SDI regions. To further reduce this burden, it is important to continue to improve global primary healthcare systems and to sustainably scale up the implementation of effective lead exposure reduction strategies, particularly in low- and middle-income countries.

## Data Availability

The datasets presented in this study can be found in online repositories. The names of the repository/repositories and accession number(s) can be found below: GHDx, http://ghdx.healthdata.org/gbd-results-tool.
