# Supplementary material for: Global, regional, and national burden of ischemic heart disease attributable to lead exposure, 1990–2021: decomposition, frontier, and projection analysis
Source: Front Public Health. 2025 Aug 18;13:1567747. doi: 10.3389/fpubh.2025.1567747 (PMC12399670; doi:10.3389/fpubh.2025.1567747)
Supplement: Supplementary file 1 [file Data_Sheet_1.docx]

**Supplementary materials**

**
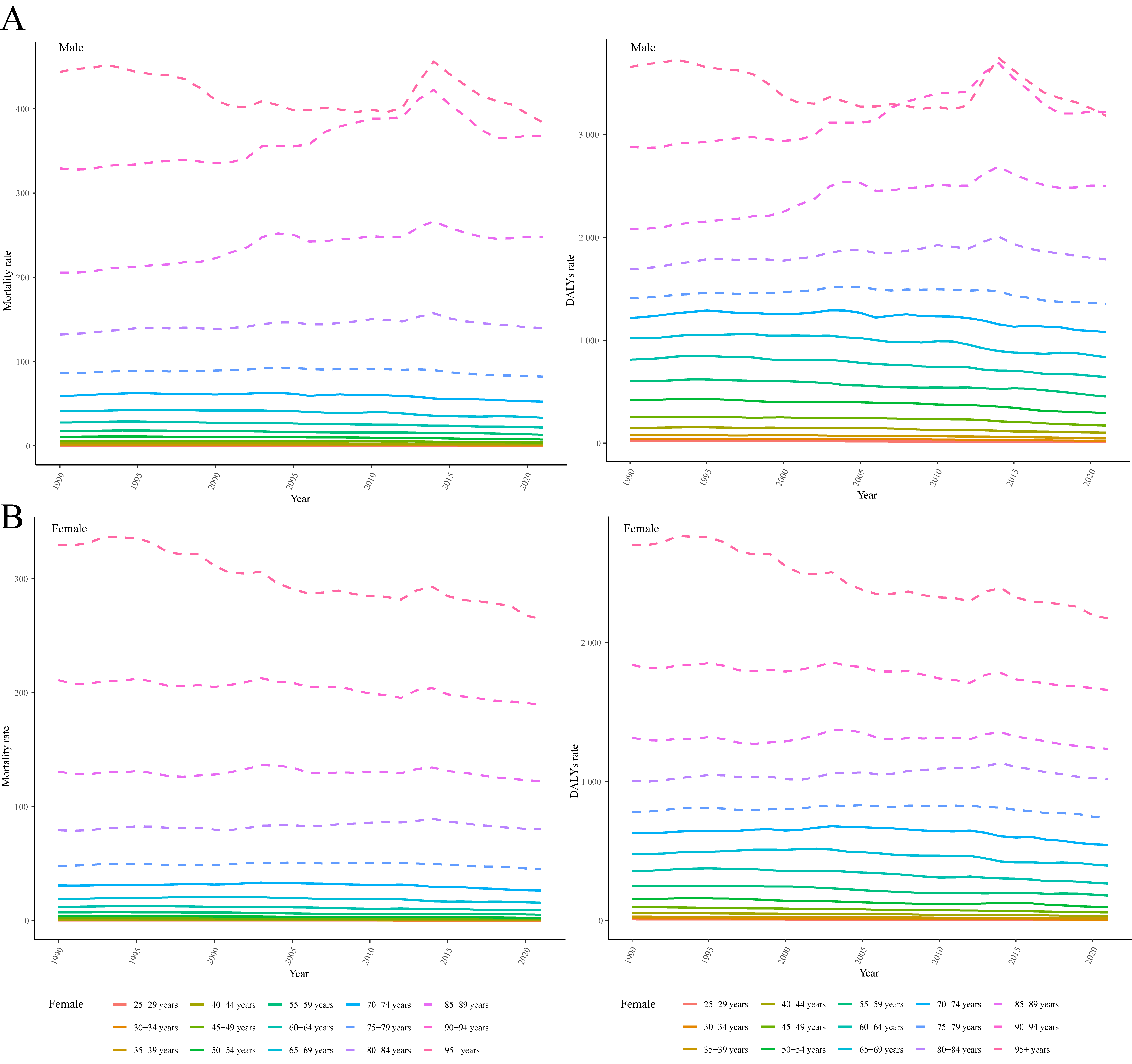
**

**Fig. S1** Trends across sex and age groups from 1990 to 2021. **(A)** Time trends in different age groups for males. **(B)** Time trends in different age groups for females. DALYs: age-standardized disability-adjusted life years.


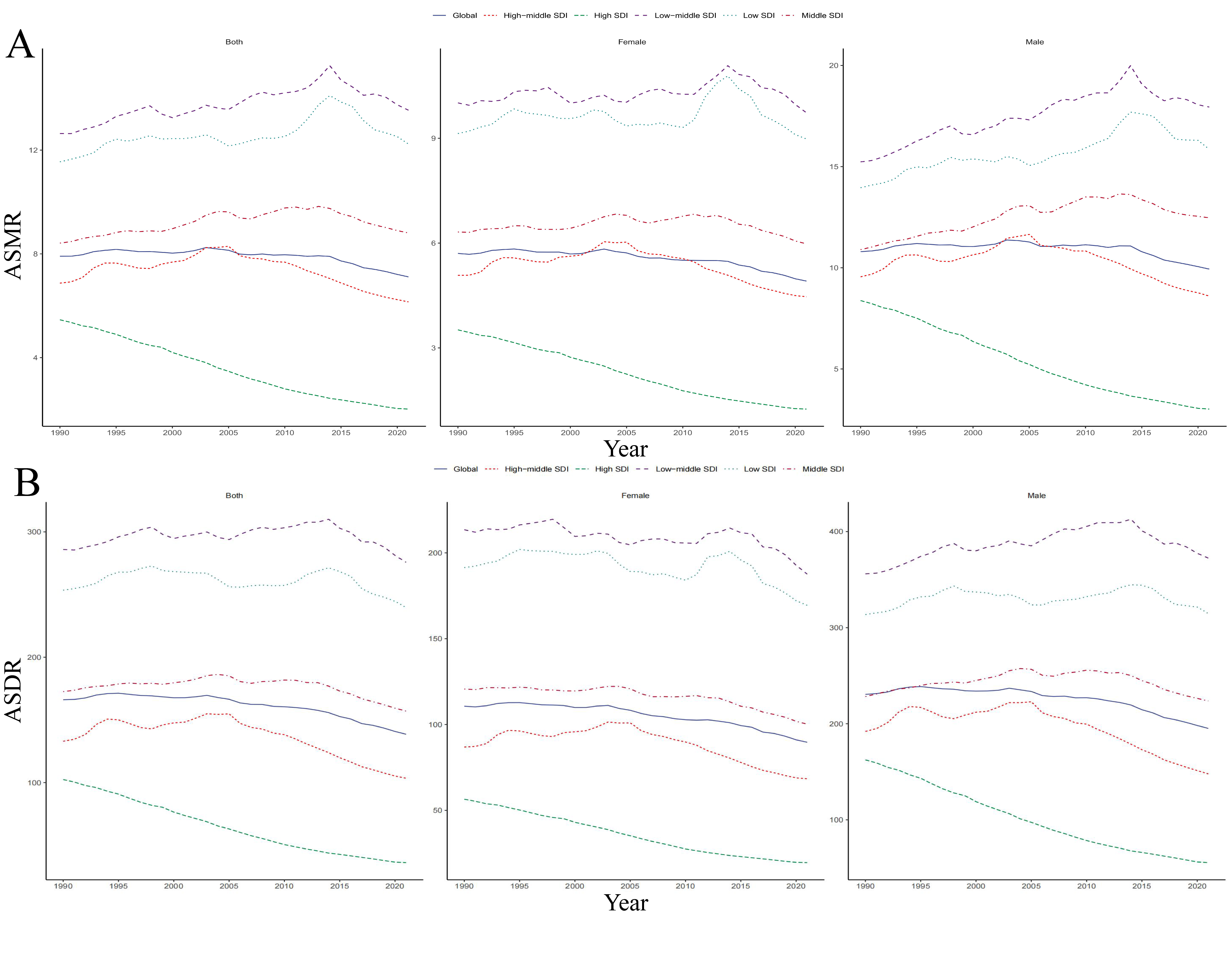


**Fig. S2** The ASRs (per 100,000 population) of IHD due to lead exposure by sex at the global and SDI quintile levels from 1990 to 2021. **(A)** ASMR. **(B)** ASDR. SDI: socio-demographic index; ASRs: age-standardized rates; IHD: Ischemic heart disease; ASMR: age-standardized death rate; ASDR: age-standardized disability-adjusted life years rate.


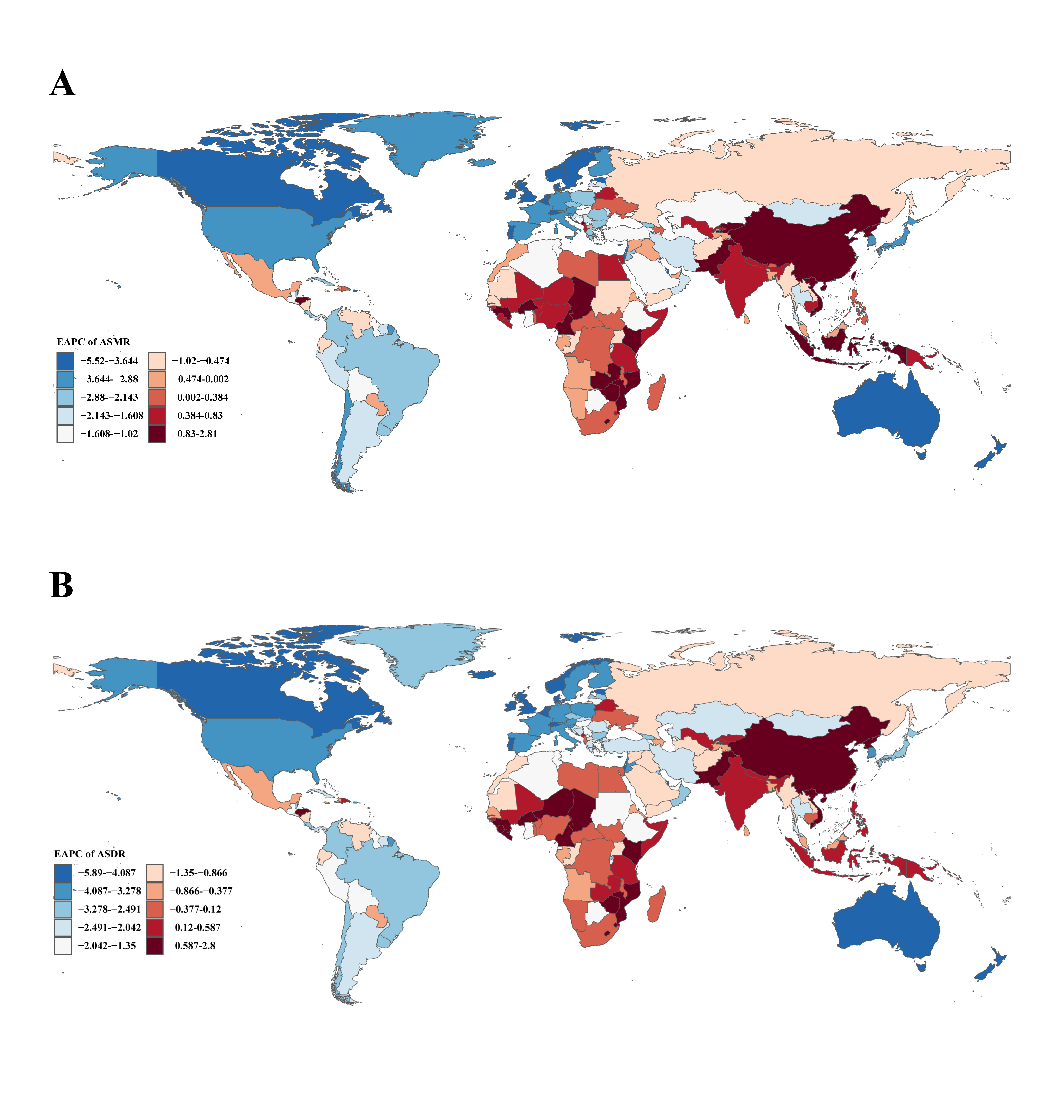


**Fig. S3** The EAPC of ASRs (per 100,000 population) of IHD due to lead exposure at the national level. **(A)** The EAPC of ASMR. **(B)** The EAPC of ASDR: Ischemic heart disease; ASRs: age-standardized rates; EAPC: estimated annual percentage change; ASMR: age-standardized death rate; ASDR: age-standardized disability-adjusted life years rate.


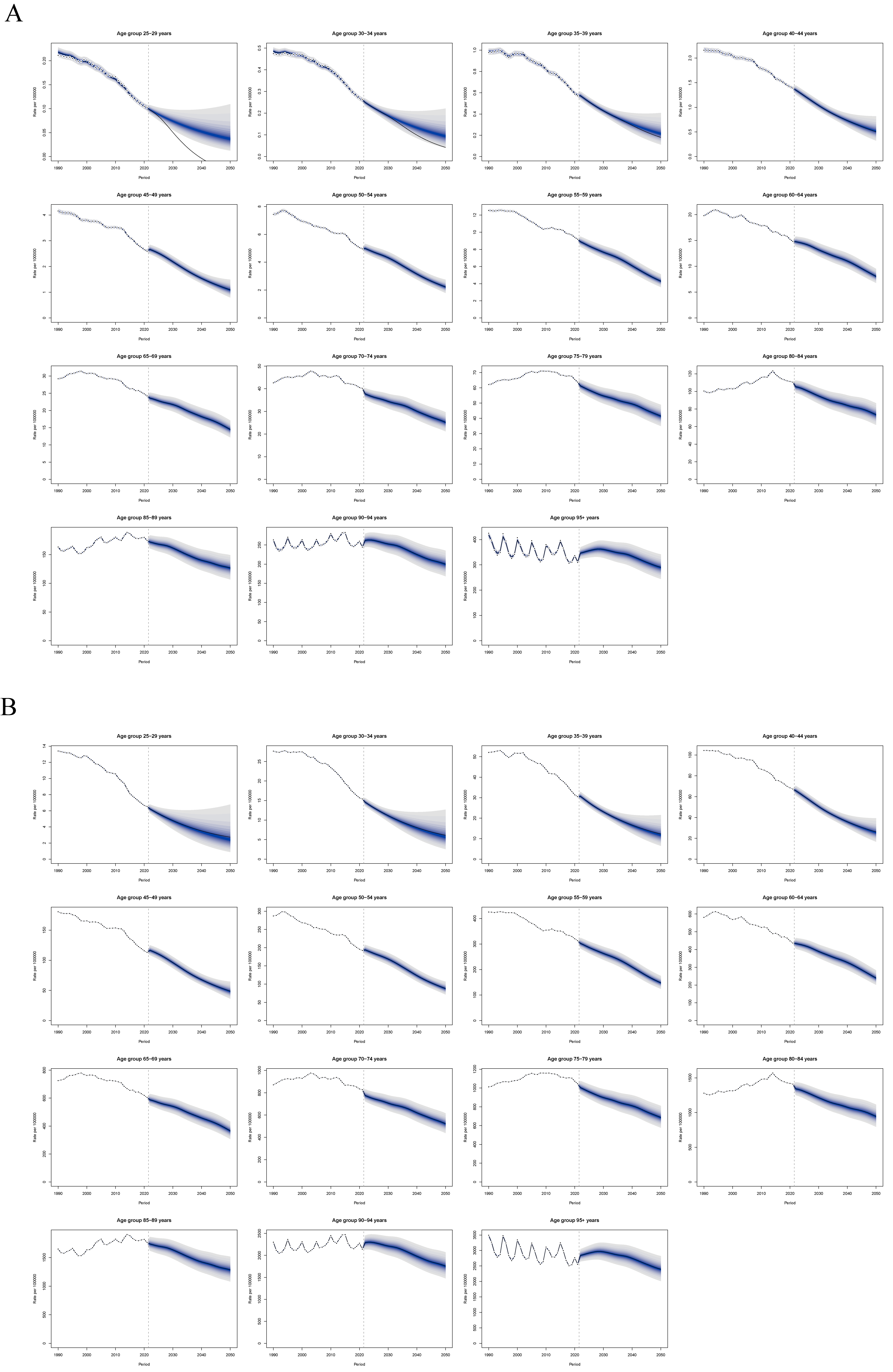


**Fig. S4** The projection of rate (per 100,000 population) in IHD due to lead exposure across different age groups globally from 2021 to 2050. **(A)** Death rate. **(B)** DALYs rate. IHD: Ischemic heart disease; DALYs: disability-adjusted life years.

**Table S1.** The death case, ASMR and EAPC of Ischemic heart disease due to lead exposure among 204 countries and territories from 1990 to 2021

| **Country** | **Death case** | | **ASMR (per 100,000 population)** | | |
| --- | --- | --- | --- | --- | --- |
|  | **1990** | **2021** | **1990** | **2021** | **1990-2021** |
|  | **Both (95% UI)** | **Both (95% UI)** | **Both (95% UI)** | **Both (95% UI)** | **EAPC(95% CI)** |
| Afghanistan | 2675.33  (-391.04-5666.55) | 2861.33  (-406.93-6112.23) | 43.29  (-6.38-90.00) | 36.91  (-5.56-78.22) | -0.69  (-0.83--0.54) |
| Albania | 133.30  (-18.31-287.68) | 339.67  (-46.80-765.43) | 7.74  (-1.07-16.74) | 8.33  (-1.15-18.80) | 0.59  (0.40-0.78) |
| Algeria | 1868.45  (-248.00-4084.25) | 3944.20  (-525.72-8650.64) | 23.71  (-3.24-50.62) | 16.80  (-2.27-36.38) | -1.10  (-1.15--1.05) |
| American Samoa | 0.40  (-0.05-0.89) | 1.02  (-0.13-2.25) | 2.36  (-0.31-5.13) | 2.57  (-0.32-5.70) | 0.38  (0.30-0.46) |
| Andorra | 1.04  (-0.14-2.47) | 2.12  (-0.27-4.76) | 2.11  (-0.28-5.02) | 1.20  (-0.15-2.69) | -1.63  (-1.86--1.40) |
| Angola | 241.72  (-33.15-527.17) | 680.43  (-91.60-1451.06) | 7.84  (-1.13-17.05) | 8.27  (-1.13-17.88) | -0.03  (-0.14-0.07) |
| Antigua and Barbuda | 3.67  (-0.49-8.03) | 3.38  (-0.50-7.31) | 6.49  (-0.87-14.16) | 3.72  (-0.55-8.03) | -2.13  (-2.37--1.88) |
| Argentina | 1390.23  (-187.44-3132.04) | 1305.23  (-182.34-2947.03) | 4.62  (-0.62-10.43) | 2.25  (-0.31-5.07) | -1.97  (-2.08--1.85) |
| Armenia | 258.03  (-34.11-568.22) | 390.61  (-54.10-888.48) | 11.33  (-1.50-25.06) | 9.05  (-1.25-20.62) | -1.47  (-1.78--1.16) |
| Australia | 1958.15  (-275.34-4260.34) | 1344.55  (-191.91-2920.30) | 10.37  (-1.46-22.53) | 2.50  (-0.36-5.45) | -4.81  (-4.93--4.68) |
| Austria | 628.61  (-90.01-1359.93) | 511.63  (-72.72-1118.45) | 5.12  (-0.73-11.07) | 2.21  (-0.31-4.83) | -3.09  (-3.36--2.83) |
| Azerbaijan | 523.18  (-71.75-1119.05) | 901.70  (-122.11-1947.39) | 12.58  (-1.72-27.14) | 12.03  (-1.66-26.00) | 0.10  (-0.17-0.37) |
| Bahamas | 6.48  (-0.90-14.17) | 9.04  (-1.21-20.33) | 4.57  (-0.63-9.96) | 2.56  (-0.34-5.75) | -2.07  (-2.25--1.89) |
| Bahrain | 24.84  (-3.38-54.85) | 39.11  (-5.32-85.77) | 21.25  (-2.89-47.70) | 8.67  (-1.21-18.56) | -3.50  (-3.98--3.02) |
| Bangladesh | 5748.03  (-940.18-12350.88) | 14156.40  (-2116.90-30367.18) | 13.02  (-2.16-27.75) | 11.72  (-1.74-25.21) | -0.25  (-0.46--0.03) |
| Barbados | 16.43  (-2.27-35.69) | 14.72  (-2.02-33.57) | 5.45  (-0.75-11.86) | 2.82  (-0.39-6.42) | -2.38  (-2.66--2.11) |
| Belarus | 1026.04  (-137.31-2220.47) | 1799.89  (-240.57-3869.70) | 8.41  (-1.13-18.20) | 10.94  (-1.46-23.55) | 0.47  (0.02-0.91) |
| Belgium | 1437.00  (-193.35-3135.50) | 748.68  (-107.12-1631.64) | 9.15  (-1.23-20.02) | 2.56  (-0.36-5.58) | -4.15  (-4.41--3.90) |
| Belize | 6.19  (-0.87-13.54) | 9.02  (-1.21-19.94) | 6.79  (-0.96-14.84) | 3.52  (-0.47-7.76) | -2.72  (-3.17--2.27) |
| Benin | 85.98  (-13.36-188.24) | 219.11  (-31.91-460.06) | 4.91  (-0.76-10.83) | 5.49  (-0.82-11.51) | 0.39  (0.25-0.53) |
| Bermuda | 4.54  (-0.64-10.04) | 3.31  (-0.41-7.40) | 7.95  (-1.12-17.56) | 2.11  (-0.26-4.75) | -4.27  (-4.50--4.04) |
| Bhutan | 26.13  (-4.45-58.03) | 72.57  (-10.97-149.79) | 12.79  (-2.18-27.98) | 13.28  (-2.03-27.40) | 0.21  (0.13-0.29) |
| Bolivia (Plurinational State of) | 242.33  (-32.12-557.55) | 474.74  (-62.43-1061.66) | 9.17  (-1.22-20.60) | 6.40  (-0.85-14.37) | -1.09  (-1.25--0.92) |
| Bosnia and Herzegovina | 391.84  (-54.23-841.49) | 528.34  (-75.53-1173.20) | 11.29  (-1.56-24.43) | 8.27  (-1.18-18.40) | -1.47  (-1.72--1.21) |
| Botswana | 36.72  (-5.23-80.73) | 66.55  (-8.06-145.45) | 8.20  (-1.16-17.88) | 5.95  (-0.75-12.81) | -1.22  (-1.57--0.87) |
| Brazil | 6678.53  (-932.58-14329.76) | 9630.66  (-1413.31-20726.88) | 8.67  (-1.22-18.68) | 3.95  (-0.58-8.50) | -2.36  (-2.43--2.30) |
| Brunei Darussalam | 6.27  (-0.88-13.61) | 8.43  (-1.22-18.75) | 7.21  (-1.02-15.56) | 3.59  (-0.53-7.82) | -1.77  (-2.02--1.51) |
| Bulgaria | 1193.77  (-167.38-2625.24) | 1081.37  (-146.87-2366.86) | 13.82  (-1.93-30.08) | 7.61  (-1.03-16.60) | -2.60  (-2.98--2.22) |
| Burkina Faso | 281.27  (-45.89-621.94) | 772.27  (-107.86-1607.66) | 7.99  (-1.30-17.92) | 10.61  (-1.53-22.11) | 1.15  (0.94-1.37) |
| Burundi | 170.59  (-22.39-365.32) | 262.42  (-32.38-556.04) | 8.55  (-1.12-18.45) | 7.31  (-0.91-15.41) | -1.01  (-1.21--0.81) |
| Cabo Verde | 10.09  (-1.39-21.80) | 30.51  (-4.36-66.92) | 4.24  (-0.58-9.14) | 7.47  (-1.07-16.35) | 1.20  (0.77-1.63) |
| Cambodia | 243.92  (-35.15-518.80) | 726.17  (-102.15-1624.92) | 6.40  (-0.93-13.91) | 7.58  (-1.06-17.08) | 0.48  (0.42-0.53) |
| Cameroon | 197.98  (-28.66-445.78) | 780.81  (-109.90-1785.23) | 5.55  (-0.82-12.24) | 8.30  (-1.21-18.62) | 1.37  (0.73-2.02) |
| Canada | 1384.03  (-188.41-3086.00) | 1177.52  (-163.80-2656.51) | 4.32  (-0.59-9.62) | 1.42  (-0.20-3.20) | -3.86  (-4.01--3.70) |
| Central African Republic | 111.82  (-17.57-242.92) | 200.33  (-31.20-419.12) | 11.98  (-1.86-26.37) | 12.47  (-1.98-26.64) | 0.02  (-0.14-0.17) |
| Chad | 207.27  (-29.01-441.28) | 531.17  (-74.81-1116.25) | 8.34  (-1.16-17.66) | 11.91  (-1.70-24.59) | 1.04  (0.73-1.36) |
| Chile | 210.82  (-28.30-453.70) | 237.44  (-32.72-511.14) | 2.40  (-0.32-5.16) | 0.90  (-0.12-1.95) | -3.00  (-3.07--2.94) |
| China | 37934.6  5(-5361.85-81521.92) | 151067.57  (-21262.11-325828.03) | 6.28  (-0.91-13.67) | 8.55  (-1.21-18.41) | 1.45  (1.04-1.86) |
| Colombia | 1697.59  (-232.98-3633.55) | 3119.72  (-441.35-6633.58) | 11.33  (-1.56-24.25) | 5.44  (-0.77-11.66) | -2.59  (-2.72--2.47) |
| Comoros | 9.91  (-1.57-21.54) | 19.18  (-2.54-40.34) | 6.33  (-1.00-13.56) | 5.01  (-0.68-10.56) | -1.03  (-1.14--0.92) |
| Congo | 77.36  (-11.49-163.13) | 145.22  (-19.28-325.83) | 8.89  (-1.32-18.95) | 7.67  (-1.03-17.02) | -0.76  (-0.91--0.61) |
| Cook Islands | 0.28  (-0.04-0.63) | 0.39  (-0.05-0.87) | 2.77  (-0.36-6.19) | 1.56  (-0.21-3.48) | -1.86  (-1.95--1.77) |
| Costa Rica | 145.07  (-20.75-308.43) | 222.44  (-31.07-487.92) | 8.96  (-1.28-19.05) | 3.92  (-0.55-8.56) | -2.68  (-2.89--2.47) |
| Croatia | 502.35  (-70.46-1112.43) | 518.76  (-72.28-1162.40) | 9.68  (-1.36-21.48) | 5.23  (-0.73-11.66) | -2.07  (-2.17--1.97) |
| Cuba | 1553.93  (-223.96-3376.40) | 1828.61  (-266.17-3838.25) | 16.10  (-2.32-34.92) | 8.65  (-1.25-18.10) | -2.28  (-2.51--2.06) |
| Cyprus | 81.36  (-12.46-175.61) | 84.27  (-12.34-182.84) | 15.14  (-2.29-33.10) | 4.94  (-0.73-10.68) | -4.01  (-4.19--3.83) |
| Czechia | 1059.62  (-148.15-2290.82) | 848.56  (-113.76-1882.63) | 7.89  (-1.10-17.06) | 3.60  (-0.48-8.00) | -2.40  (-2.52--2.29) |
| Côte d'Ivoire | 244.69  (-32.86-526.33) | 738.44  (-105.81-1618.73) | 8.27  (-1.15-18.17) | 9.18  (-1.33-20.10) | 0.16  (-0.16-0.49) |
| Democratic People's Republic of Korea | 759.73  (-106.54-1627.51) | 2309.23  (-304.81-5006.81) | 6.09  (-0.87-13.20) | 8.03  (-1.07-17.61) | 0.93  (0.66-1.21) |
| Democratic Republic of the Congo | 886.07  (-123.62-1878.80) | 2088.33  (-309.58-4535.45) | 7.36  (-1.03-15.87) | 7.77  (-1.14-16.94) | 0.07  (0.02-0.11) |
| Denmark | 567.13  (-82.21-1238.00) | 190.59  (-27.64-426.33) | 6.62  (-0.96-14.44) | 1.39  (-0.20-3.10) | -5.50  (-5.68--5.33) |
| Djibouti | 5.44  (-0.69-12.25) | 29.27  (-3.41-67.72) | 5.50  (-0.73-12.13) | 6.82  (-0.82-15.11) | 0.58  (0.34-0.82) |
| Dominica | 4.58  (-0.62-10.00) | 3.43  (-0.48-7.37) | 8.00  (-1.08-17.51) | 4.51  (-0.64-9.70) | -2.09  (-2.27--1.91) |
| Dominican Republic | 473.62  (-71.76-996.96) | 1397.69  (-181.79-2988.00) | 15.67  (-2.36-33.17) | 14.55  (-1.89-31.11) | 0.38  (0.12-0.64) |
| Ecuador | 201.63  (-27.04-436.12) | 552.32  (-76.10-1227.16) | 4.51  (-0.61-9.74) | 3.82  (-0.53-8.43) | -0.60  (-1.11--0.08) |
| Egypt | 8155.24  (-1217.53-17376.01) | 17450.97  (-2469.24-36505.28) | 40.92  (-6.21-86.87) | 40.35  (-5.93-85.13) | 0.40  (0.13-0.66) |
| El Salvador | 400.82  (-59.21-844.37) | 760.64  (-108.00-1591.31) | 14.18  (-2.10-29.79) | 11.12  (-1.58-23.15) | -0.90  (-1.07--0.73) |
| Equatorial Guinea | 14.77  (-2.33-31.47) | 28.12  (-3.75-64.78) | 9.12  (-1.42-19.69) | 7.86  (-1.10-17.71) | -0.70  (-0.85--0.56) |
| Eritrea | 54.81  (-6.84-121.49) | 118.90  (-14.87-262.68) | 6.43  (-0.80-13.68) | 6.34  (-0.82-13.83) | -0.16  (-0.33-0.02) |
| Estonia | 136.47  (-18.55-294.92) | 75.13  (-10.72-168.39) | 7.08  (-0.96-15.38) | 2.29  (-0.33-5.11) | -4.05  (-4.59--3.50) |
| Eswatini | 16.10  (-2.73-34.89) | 32.57  (-4.95-72.60) | 6.79  (-1.15-14.64) | 7.31  (-1.11-15.85) | 0.83  (0.20-1.46) |
| Ethiopia | 1298.12  (-183.52-2820.85) | 1912.03  (-251.47-4159.31) | 8.07  (-1.17-17.70) | 5.59  (-0.74-12.10) | -1.45  (-1.59--1.32) |
| Fiji | 19.95  (-2.62-44.41) | 33.49  (-4.23-74.73) | 7.01  (-0.91-15.69) | 5.67  (-0.73-12.39) | -0.96  (-1.16--0.76) |
| Finland | 251.93  (-35.07-556.57) | 228.66  (-31.56-515.84) | 3.51  (-0.49-7.78) | 1.43  (-0.20-3.24) | -2.92  (-3.06--2.77) |
| France | 3321.26  (-468.12-7182.72) | 2559.81  (-360.76-5678.27) | 3.77  (-0.53-8.11) | 1.30  (-0.18-2.83) | -3.63  (-3.86--3.40) |
| Gabon | 30.45  (-4.79-66.62) | 43.11  (-5.54-94.00) | 6.05  (-0.95-13.20) | 5.54  (-0.71-12.37) | -0.40  (-0.58--0.22) |
| Gambia | 24.61  (-3.54-55.60) | 92.27  (-12.93-193.63) | 8.76  (-1.25-19.57) | 11.78  (-1.64-24.75) | 0.83  (0.62-1.04) |
| Georgia | 790.13  (-113.54-1696.84) | 450.57  (-66.70-974.91) | 13.63  (-1.96-29.00) | 7.08  (-1.05-15.31) | -2.73  (-3.24--2.21) |
| Germany | 5332.70  (-743.88-11670.61) | 3797.84  (-519.50-8499.96) | 4.05  (-0.56-8.88) | 1.58  (-0.22-3.55) | -3.21  (-3.35--3.07) |
| Ghana | 348.00  (-47.98-774.25) | 629.12  (-90.35-1344.33) | 6.99  (-0.98-15.51) | 5.07  (-0.74-10.86) | -1.57  (-1.92--1.21) |
| Greece | 1035.49  (-144.68-2262.29) | 1157.82  (-166.30-2536.60) | 7.09  (-0.99-15.60) | 3.83  (-0.55-8.32) | -2.17  (-2.60--1.74) |
| Greenland | 1.27  (-0.17-2.77) | 1.11  (-0.15-2.48) | 5.04  (-0.70-11.18) | 2.06  (-0.28-4.64) | -3.00  (-3.11--2.88) |
| Grenada | 8.53  (-1.15-18.51) | 6.86  (-0.97-14.71) | 11.17  (-1.50-24.27) | 6.96  (-0.98-14.94) | -2.14  (-2.48--1.80) |
| Guam | 1.45  (-0.19-3.15) | 3.48  (-0.45-7.86) | 2.94  (-0.39-6.36) | 1.59  (-0.21-3.58) | -1.22  (-1.52--0.92) |
| Guatemala | 463.82  (-66.86-960.45) | 1153.09  (-170.38-2383.07) | 19.12  (-2.80-39.49) | 13.12  (-1.96-27.44) | -1.20  (-1.58--0.82) |
| Guinea | 213.58  (-34.28-445.04) | 481.54  (-67.37-986.82) | 7.40  (-1.19-15.49) | 10.48  (-1.48-21.50) | 1.35  (1.11-1.58) |
| Guinea-Bissau | 37.56  (-5.79-81.06) | 68.64  (-9.10-144.84) | 11.38  (-1.77-24.52) | 13.21  (-1.74-27.68) | 0.52  (0.34-0.69) |
| Guyana | 61.20  (-8.45-136.46) | 61.40  (-8.33-135.32) | 18.24  (-2.53-40.55) | 11.32  (-1.54-24.75) | -1.32  (-1.65--0.98) |
| Haiti | 811.80  (-123.17-1666.99) | 1449.39  (-219.15-3092.02) | 31.25  (-4.82-63.90) | 26.63  (-4.09-56.53) | -0.37  (-0.41--0.33) |
| Honduras | 232.95  (-32.79-497.57) | 1054.47  (-151.62-2188.16) | 13.97  (-1.98-30.13) | 21.56  (-3.15-44.76) | 1.60  (1.30-1.90) |
| Hungary | 1125.77  (-157.50-2426.00) | 1234.41  (-168.14-2688.41) | 8.24  (-1.15-17.85) | 5.73  (-0.78-12.46) | -1.24  (-1.45--1.02) |
| Iceland | 18.99  (-2.60-41.97) | 14.65  (-1.93-32.71) | 6.28  (-0.86-13.88) | 2.09  (-0.27-4.62) | -3.59  (-3.80--3.38) |
| India | 50980.12  (-7713.14-109306.48) | 148675.35  (-21123.95-310126.63) | 12.40  (-1.88-26.22) | 14.18  (-2.03-29.39) | 0.66  (0.48-0.84) |
| Indonesia | 5709.91  (-857.30-12194.84) | 16772.90  (-2505.09-36179.36) | 6.55  (-1.01-14.16) | 9.15  (-1.38-19.42) | 1.18  (0.98-1.37) |
| Iran (Islamic Republic of) | 6086.43  (-894.71-12854.54) | 11586.73  (-1717.25-24653.21) | 30.88  (-4.56-65.22) | 17.63  (-2.63-37.38) | -2.03  (-2.21--1.85) |
| Iraq | 1346.96  (-193.05-2898.14) | 3344.89  (-454.81-7473.64) | 18.18  (-2.60-38.94) | 19.04  (-2.61-42.74) | -0.37  (-0.55--0.19) |
| Ireland | 383.08  (-52.73-831.04) | 188.67  (-26.81-424.44) | 9.59  (-1.32-20.80) | 2.20  (-0.31-4.96) | -4.80  (-5.02--4.58) |
| Israel | 246.44  (-34.35-539.59) | 160.22  (-22.33-353.86) | 5.41  (-0.75-11.91) | 1.13  (-0.16-2.50) | -5.52  (-5.70--5.35) |
| Italy | 4588.87  (-668.65-9926.42) | 4201.75  (-592.72-9297.61) | 5.23  (-0.76-11.35) | 2.09  (-0.29-4.61) | -3.10  (-3.31--2.89) |
| Jamaica | 98.99  (-13.54-213.80) | 130.02  (-16.86-284.67) | 5.37  (-0.73-11.61) | 3.82  (-0.50-8.33) | -0.76  (-1.20--0.32) |
| Japan | 3032.32  (-425.82-6679.05) | 3619.42  (-509.92-8089.45) | 1.97  (-0.28-4.34) | 0.68  (-0.10-1.51) | -3.25  (-3.43--3.08) |
| Jordan | 143.19  (-18.65-311.63) | 346.17  (-48.24-753.71) | 13.02  (-1.73-28.52) | 6.48  (-0.91-13.97) | -2.77  (-3.13--2.42) |
| Kazakhstan | 890.31  (-124.16-1975.93) | 925.66  (-130.92-1994.31) | 8.17  (-1.14-18.26) | 7.03  (-0.99-15.11) | -1.60  (-2.24--0.95) |
| Kenya | 189.34  (-27.79-428.36) | 611.68  (-86.53-1427.08) | 2.92  (-0.43-6.62) | 3.80  (-0.54-8.95) | 1.06  (0.72-1.40) |
| Kiribati | 1.51  (-0.20-3.37) | 3.03  (-0.38-6.75) | 5.02  (-0.64-11.18) | 5.34  (-0.68-11.83) | 0.19  (0.11-0.28) |
| Kuwait | 57.17  (-8.06-124.46) | 132.89  (-18.66-286.18) | 11.25  (-1.61-24.10) | 5.76  (-0.82-12.50) | -2.11  (-2.61--1.61) |
| Kyrgyzstan | 247.57  (-34.63-546.41) | 444.36  (-61.95-991.70) | 9.64  (-1.35-21.35) | 12.57  (-1.77-27.94) | 1.19  (0.74-1.65) |
| Lao People's Democratic Republic | 280.21  (-40.15-621.00) | 489.54  (-70.95-1061.45) | 15.54  (-2.29-33.88) | 13.49  (-1.93-29.10) | -0.59  (-0.65--0.53) |
| Latvia | 245.46  (-34.11-539.08) | 190.37  (-25.99-414.28) | 7.10  (-0.99-15.63) | 4.13  (-0.56-8.95) | -2.08  (-2.48--1.69) |
| Lebanon | 208.47  (-28.45-478.56) | 304.69  (-40.81-665.04) | 11.22  (-1.54-25.78) | 4.58  (-0.61-9.98) | -2.97  (-3.17--2.76) |
| Lesotho | 28.21  (-4.34-61.34) | 62.42  (-8.33-143.16) | 3.82  (-0.60-8.30) | 7.00  (-0.97-15.84) | 2.81  (2.24-3.37) |
| Liberia | 72.67  (-9.86-157.02) | 151.62  (-21.62-326.56) | 7.51  (-1.02-16.16) | 9.69  (-1.38-20.68) | 0.78  (0.54-1.03) |
| Libya | 223.24  (-31.62-509.51) | 530.57  (-68.32-1187.83) | 13.09  (-1.87-29.59) | 12.61  (-1.62-28.23) | 0.37  (0.06-0.69) |
| Lithuania | 340.95  (-45.33-731.63) | 349.15  (-50.46-769.87) | 7.78  (-1.04-16.73) | 5.10  (-0.73-11.24) | -1.36  (-1.67--1.05) |
| Luxembourg | 27.79  (-3.76-61.77) | 18.95  (-2.55-43.00) | 5.22  (-0.71-11.59) | 1.55  (-0.21-3.50) | -4.05  (-4.25--3.86) |
| Madagascar | 253.93  (-35.70-558.49) | 533.03  (-63.54-1194.53) | 6.02  (-0.84-13.27) | 6.82  (-0.80-15.37) | 0.04  (-0.18-0.27) |
| Malawi | 174.00  (-26.04-369.48) | 391.30  (-54.81-811.26) | 5.77  (-0.87-11.82) | 6.76  (-0.95-13.97) | 0.28  (-0.10-0.65) |
| Malaysia | 657.71  (-96.30-1458.15) | 1772.67  (-251.87-3723.97) | 7.62  (-1.12-17.03) | 7.09  (-1.02-14.80) | -0.41  (-0.68--0.14) |
| Maldives | 6.68  (-0.99-14.55) | 12.49  (-1.59-26.85) | 9.14  (-1.40-19.94) | 4.57  (-0.58-9.83) | -2.66  (-2.80--2.51) |
| Mali | 206.26  (-31.70-432.63) | 505.77  (-73.22-1098.64) | 6.63  (-1.02-13.84) | 7.60  (-1.11-16.24) | 0.56  (0.49-0.64) |
| Malta | 75.38  (-11.54-155.43) | 70.57  (-10.52-148.80) | 18.63  (-2.84-38.33) | 6.32  (-0.94-13.29) | -3.42  (-3.70--3.14) |
| Marshall Islands | 0.93  (-0.13-2.04) | 1.76  (-0.25-3.87) | 7.27  (-1.00-16.18) | 7.21  (-1.08-16.00) | 0.13  (-0.03-0.29) |
| Mauritania | 57.21  (-7.77-128.15) | 108.55  (-14.19-236.58) | 6.67  (-0.91-15.02) | 6.24  (-0.82-13.59) | -0.53  (-0.67--0.39) |
| Mauritius | 47.12  (-6.11-103.63) | 57.53  (-7.74-126.07) | 7.36  (-0.96-16.12) | 3.36  (-0.45-7.37) | -3.03  (-3.35--2.71) |
| Mexico | 3166.74  (-446.47-6663.64) | 10413.75  (-1535.14-22595.66) | 9.39  (-1.33-19.83) | 9.35  (-1.38-20.31) | -0.14  (-0.34-0.06) |
| Micronesia (Federated States of) | 2.64  (-0.37-5.92) | 3.69  (-0.53-8.36) | 6.15  (-0.86-13.80) | 6.21  (-0.88-13.86) | 0.07  (-0.02-0.16) |
| Monaco | 2.30  (-0.33-5.16) | 1.84  (-0.27-4.12) | 2.93  (-0.42-6.54) | 1.51  (-0.22-3.36) | -2.31  (-2.45--2.18) |
| Mongolia | 156.85  (-21.74-337.07) | 186.97  (-23.72-403.53) | 18.42  (-2.56-39.88) | 12.49  (-1.59-27.05) | -1.74  (-2.07--1.40) |
| Montenegro | 25.57  (-3.57-56.83) | 55.48  (-7.42-123.73) | 4.37  (-0.61-9.71) | 6.58  (-0.88-14.60) | 1.44  (1.23-1.65) |
| Morocco | 2527.42  (-341.73-5468.55) | 4977.32  (-637.19-10711.65) | 20.03  (-2.73-43.10) | 17.60  (-2.28-37.56) | -0.44  (-0.48--0.39) |
| Mozambique | 162.98  (-26.09-343.31) | 404.54  (-54.22-845.49) | 3.63  (-0.57-7.68) | 4.90  (-0.68-10.20) | 1.39  (1.12-1.65) |
| Myanmar | 2210.52  (-292.62-4855.61) | 3706.57  (-507.53-8012.37) | 11.13  (-1.51-24.19) | 9.18  (-1.27-19.73) | -0.79  (-0.95--0.62) |
| Namibia | 31.99  (-4.79-69.23) | 67.94  (-9.12-149.45) | 6.04  (-0.90-13.17) | 6.22  (-0.83-13.60) | -0.00  (-0.37-0.36) |
| Nauru | 0.24  (-0.03-0.52) | 0.34  (-0.05-0.76) | 6.92  (-0.83-14.91) | 7.49  (-1.02-16.69) | 0.28  (-0.04-0.60) |
| Nepal | 1252.21  (-184.99-2714.09) | 3414.92  (-459.50-7051.63) | 15.68  (-2.34-33.75) | 17.54  (-2.37-36.07) | 0.63  (0.49-0.78) |
| Netherlands | 1037.06  (-148.27-2285.35) | 537.81  (-74.31-1205.65) | 5.09  (-0.73-11.22) | 1.33  (-0.18-2.99) | -4.86  (-5.09--4.63) |
| New Zealand | 350.91  (-49.25-756.80) | 291.87  (-42.38-630.33) | 9.06  (-1.27-19.54) | 3.15  (-0.46-6.81) | -3.65  (-3.78--3.52) |
| Nicaragua | 136.04  (-20.06-281.07) | 324.97  (-47.81-681.76) | 10.72  (-1.58-22.22) | 8.30  (-1.23-17.38) | -0.49  (-0.79--0.20) |
| Niger | 143.35  (-19.74-318.05) | 526.84  (-79.79-1126.14) | 6.96  (-0.99-15.33) | 8.81  (-1.35-18.41) | 0.81  (0.71-0.92) |
| Nigeria | 2015.02  (-270.31-4506.80) | 4154.31  (-524.85-9078.12) | 5.50  (-0.74-12.33) | 6.16  (-0.79-13.26) | 0.41  (0.22-0.59) |
| Niue | 0.10  (-0.01-0.23) | 0.08  (-0.01-0.17) | 4.28  (-0.57-9.43) | 3.85  (-0.52-8.51) | -0.44  (-0.56--0.32) |
| North Macedonia | 125.27  (-17.54-282.46) | 187.88  (-24.64-418.04) | 7.41  (-1.04-16.72) | 7.96  (-1.06-17.65) | -0.15  (-0.68-0.39) |
| Northern Mariana Islands | 0.19  (-0.03-0.42) | 0.73  (-0.09-1.62) | 1.62  (-0.21-3.53) | 1.86  (-0.24-4.17) | 0.67  (0.49-0.85) |
| Norway | 441.37  (-59.82-958.53) | 170.91  (-23.66-373.74) | 6.01  (-0.81-13.07) | 1.42  (-0.20-3.09) | -4.93  (-5.21--4.64) |
| Oman | 121.61  (-16.25-274.09) | 144.54  (-18.78-327.35) | 21.24  (-2.93-48.07) | 11.35  (-1.52-25.69) | -1.80  (-2.00--1.61) |
| Pakistan | 6711.59  (-1032.42-14585.67) | 17984.62  (-2743.22-38822.04) | 13.37  (-2.06-28.93) | 18.41  (-2.82-39.44) | 0.99  (0.78-1.20) |
| Palau | 0.26  (-0.04-0.58) | 0.50  (-0.06-1.07) | 3.29  (-0.45-7.30) | 3.03  (-0.40-6.56) | -0.08  (-0.18-0.03) |
| Palestine | 206.74  (-28.62-439.79) | 294.79  (-42.27-639.78) | 28.92  (-4.02-60.83) | 17.33  (-2.50-37.44) | -1.80  (-2.18--1.42) |
| Panama | 105.51  (-15.44-226.37) | 181.38  (-25.61-404.07) | 7.77  (-1.14-16.63) | 3.93  (-0.55-8.76) | -2.06  (-2.27--1.85) |
| Papua New Guinea | 51.44  (-7.45-120.62) | 157.19  (-19.66-360.95) | 3.75  (-0.54-8.87) | 4.36  (-0.56-10.14) | 0.56  (0.33-0.79) |
| Paraguay | 121.83  (-17.05-261.40) | 268.02  (-35.10-597.89) | 5.99  (-0.84-12.82) | 4.98  (-0.66-11.10) | -0.28  (-0.46--0.09) |
| Peru | 433.35  (-54.96-978.08) | 912.74  (-118.68-2051.31) | 3.98  (-0.51-8.95) | 2.73  (-0.36-6.11) | -1.63  (-2.06--1.20) |
| Philippines | 1294.30  (-178.82-2870.48) | 4181.98  (-564.76-9201.31) | 5.52  (-0.77-12.07) | 5.93  (-0.81-13.06) | 0.32  (0.23-0.41) |
| Poland | 4512.54  (-625.71-9710.54) | 3880.93  (-569.78-8589.80) | 10.93  (-1.51-23.47) | 4.93  (-0.72-10.91) | -2.86  (-2.96--2.75) |
| Portugal | 1052.97  (-150.65-2274.60) | 773.29  (-108.29-1690.33) | 8.26  (-1.19-17.86) | 2.55  (-0.35-5.61) | -4.30  (-4.57--4.03) |
| Puerto Rico | 202.88  (-27.46-450.60) | 168.40  (-23.02-369.72) | 6.04  (-0.82-13.38) | 1.90  (-0.26-4.19) | -4.06  (-4.25--3.86) |
| Qatar | 10.33  (-1.33-22.91) | 22.25  (-3.08-49.18) | 15.62  (-2.04-34.22) | 5.10  (-0.72-11.18) | -4.51  (-5.40--3.61) |
| Republic of Korea | 648.71  (-85.66-1447.70) | 1285.34  (-183.44-2939.58) | 3.15  (-0.43-7.02) | 1.43  (-0.20-3.26) | -3.10  (-3.45--2.75) |
| Republic of Moldova | 362.50  (-50.03-788.00) | 487.15  (-66.80-1031.30) | 10.59  (-1.47-23.26) | 8.04  (-1.10-17.03) | -1.61  (-2.03--1.19) |
| Romania | 2353.36  (-328.95-5146.72) | 2511.88  (-371.40-5587.15) | 10.33  (-1.43-22.73) | 6.11  (-0.90-13.62) | -2.21  (-2.44--1.97) |
| Russian Federation | 10680.17  (-1444.09-23306.82) | 14118.94  (-1853.58-31039.78) | 6.77  (-0.92-14.79) | 5.83  (-0.76-12.80) | -0.89  (-1.51--0.27) |
| Rwanda | 160.65  (-22.44-354.08) | 208.93  (-25.28-455.35) | 7.14  (-0.97-15.63) | 4.67  (-0.58-10.10) | -2.26  (-2.59--1.92) |
| Saint Kitts and Nevis | 4.33  (-0.59-9.48) | 2.48  (-0.34-5.52) | 11.91  (-1.63-26.07) | 4.74  (-0.65-10.56) | -2.91  (-3.12--2.69) |
| Saint Lucia | 9.58  (-1.39-20.65) | 8.86  (-1.27-19.96) | 13.20  (-1.92-28.38) | 3.90  (-0.56-8.79) | -4.56  (-4.94--4.18) |
| Saint Vincent and the Grenadines | 10.96  (-1.58-23.67) | 11.23  (-1.54-24.52) | 16.78  (-2.42-36.14) | 9.14  (-1.27-19.94) | -2.21  (-2.46--1.96) |
| Samoa | 3.23  (-0.44-7.28) | 5.84  (-0.76-12.77) | 4.67  (-0.64-10.62) | 4.88  (-0.64-10.68) | 0.16  (0.05-0.27) |
| San Marino | 0.84  (-0.11-1.82) | 0.71  (-0.08-1.57) | 2.24  (-0.29-4.87) | 0.67  (-0.08-1.48) | -3.33  (-3.70--2.95) |
| Sao Tome and Principe | 2.75  (-0.39-5.97) | 6.05  (-0.74-13.19) | 4.80  (-0.68-10.35) | 7.09  (-0.88-15.32) | 1.47  (1.22-1.72) |
| Saudi Arabia | 913.60  (-135.19-2117.37) | 1897.90  (-254.38-4056.18) | 18.07  (-2.69-41.72) | 13.31  (-1.91-28.29) | -1.09  (-1.40--0.78) |
| Senegal | 235.91  (-35.40-507.83) | 494.43  (-74.80-1091.80) | 8.89  (-1.34-19.25) | 8.21  (-1.27-17.91) | -0.51  (-0.68--0.34) |
| Serbia | 808.74  (-113.36-1773.81) | 1130.53  (-159.43-2441.01) | 9.64  (-1.35-21.25) | 6.54  (-0.92-14.09) | -1.91  (-2.22--1.60) |
| Seychelles | 2.05  (-0.27-4.61) | 2.48  (-0.34-5.50) | 3.67  (-0.49-8.24) | 2.45  (-0.33-5.44) | -1.23  (-1.38--1.08) |
| Sierra Leone | 160.14  (-23.65-353.45) | 323.22  (-47.02-685.06) | 8.89  (-1.31-19.37) | 10.70  (-1.55-22.55) | 0.78  (0.46-1.09) |
| Singapore | 184.85  (-25.66-394.72) | 226.87  (-31.75-490.17) | 9.37  (-1.30-20.03) | 2.73  (-0.38-5.92) | -4.24  (-4.56--3.92) |
| Slovakia | 522.44  (-73.88-1129.10) | 555.55  (-76.33-1232.12) | 9.11  (-1.29-19.69) | 5.84  (-0.80-12.96) | -1.39  (-1.53--1.25) |
| Slovenia | 78.49  (-10.71-172.59) | 72.29  (-9.36-161.71) | 3.24  (-0.44-7.11) | 1.34  (-0.17-3.01) | -3.42  (-3.64--3.19) |
| Solomon Islands | 15.22  (-2.08-31.89) | 34.66  (-4.69-76.97) | 16.02  (-2.16-34.02) | 13.93  (-1.87-31.37) | -0.47  (-0.54--0.41) |
| Somalia | 136.23  (-22.14-283.26) | 383.63  (-63.69-821.28) | 7.46  (-1.27-15.26) | 8.48  (-1.39-18.32) | 0.50  (0.30-0.70) |
| South Africa | 595.72  (-77.51-1346.41) | 1399.70  (-186.54-3038.19) | 3.17  (-0.42-7.15) | 3.66  (-0.49-7.99) | 0.33  (-0.20-0.86) |
| South Sudan | 131.16  (-20.12-279.98) | 188.55  (-26.50-402.50) | 5.95  (-0.94-12.66) | 6.59  (-0.94-13.72) | 0.21  (0.09-0.32) |
| Spain | 2870.18  (-414.96-6293.69) | 2570.62  (-362.84-5562.49) | 5.37  (-0.78-11.79) | 2.02  (-0.29-4.41) | -3.38  (-3.61--3.14) |
| Sri Lanka | 332.00  (-45.00-726.54) | 730.23  (-90.02-1708.18) | 3.69  (-0.50-8.10) | 2.96  (-0.37-6.93) | -0.31  (-0.64-0.01) |
| Sudan | 2973.18  (-428.01-6444.58) | 4485.51  (-594.73-9891.37) | 36.67  (-5.36-78.21) | 28.41  (-3.76-61.78) | -1.00  (-1.10--0.90) |
| Suriname | 29.41  (-3.99-64.38) | 37.61  (-5.54-85.71) | 12.50  (-1.71-27.33) | 6.31  (-0.94-14.44) | -2.06  (-2.28--1.84) |
| Sweden | 630.47  (-87.13-1387.24) | 328.56  (-45.42-728.08) | 3.80  (-0.53-8.39) | 1.20  (-0.16-2.65) | -3.70  (-3.90--3.51) |
| Switzerland | 610.61  (-82.15-1340.19) | 407.45  (-56.84-917.03) | 5.46  (-0.73-12.03) | 1.68  (-0.23-3.75) | -4.03  (-4.21--3.85) |
| Syrian Arab Republic | 1540.28  (-208.72-3257.65) | 3064.00  (-427.95-6413.17) | 34.13  (-4.64-71.93) | 32.49  (-4.67-67.66) | -0.47  (-0.63--0.31) |
| Taiwan (Province of China) | 514.64  (-72.70-1144.61) | 867.72  (-125.09-1919.85) | 4.20  (-0.60-9.38) | 1.94  (-0.28-4.28) | -2.46  (-2.62--2.29) |
| Tajikistan | 367.33  (-52.25-815.87) | 581.88  (-87.97-1267.31) | 15.32  (-2.17-33.89) | 15.06  (-2.32-32.67) | -0.21  (-0.64-0.22) |
| Thailand | 696.72  (-94.43-1525.77) | 1661.90  (-206.98-3829.02) | 2.36  (-0.32-5.22) | 1.52  (-0.19-3.51) | -2.06  (-2.41--1.72) |
| Timor-Leste | 20.25  (-2.95-45.21) | 90.63  (-12.20-195.79) | 9.08  (-1.35-20.42) | 13.06  (-1.74-28.14) | 1.51  (1.37-1.66) |
| Togo | 70.57  (-10.77-149.47) | 227.70  (-30.16-496.77) | 7.24  (-1.11-15.37) | 8.26  (-1.13-17.62) | 0.13  (-0.18-0.45) |
| Tokelau | 0.06  (-0.01-0.13) | 0.06  (-0.01-0.13) | 4.74  (-0.65-10.49) | 3.89  (-0.48-8.78) | -0.66  (-0.80--0.52) |
| Tonga | 1.92  (-0.24-4.19) | 2.58  (-0.32-5.73) | 4.15  (-0.53-9.09) | 3.41  (-0.42-7.57) | -0.58  (-0.75--0.40) |
| Trinidad and Tobago | 56.78  (-7.72-127.63) | 63.43  (-8.21-144.35) | 8.07  (-1.11-17.80) | 3.39  (-0.44-7.74) | -3.36  (-3.62--3.11) |
| Tunisia | 677.20  (-101.57-1432.22) | 1442.31  (-183.28-3167.42) | 17.04  (-2.59-35.81) | 12.49  (-1.60-27.39) | -1.33  (-1.55--1.11) |
| Turkey | 3338.05  (-488.66-7096.31) | 5687.14  (-796.96-12511.90) | 11.20  (-1.65-24.07) | 6.91  (-0.97-15.18) | -1.47  (-1.72--1.21) |
| Turkmenistan | 200.84  (-28.39-442.99) | 386.42  (-53.32-835.83) | 13.05  (-1.85-28.59) | 12.30  (-1.70-26.60) | -1.13  (-1.59--0.67) |
| Tuvalu | 0.45  (-0.06-0.99) | 0.65  (-0.09-1.40) | 8.50  (-1.21-18.72) | 7.61  (-1.09-16.53) | -0.31  (-0.44--0.17) |
| Uganda | 295.87  (-47.99-635.13) | 633.35  (-84.72-1335.66) | 5.68  (-0.94-12.19) | 5.58  (-0.78-11.83) | -0.64  (-1.10--0.19) |
| Ukraine | 4369.40  (-576.57-9573.84) | 6951.55  (-871.64-15810.19) | 6.85  (-0.90-15.03) | 8.67  (-1.09-19.74) | 0.16  (-0.49-0.81) |
| United Arab Emirates | 45.18  (-6.25-103.41) | 97.90  (-12.88-217.22) | 13.50  (-1.92-30.59) | 7.56  (-1.07-16.75) | -0.30  (-0.89-0.29) |
| United Kingdom | 6148.03  (-852.38-13255.40) | 2488.07  (-344.13-5478.45) | 6.54  (-0.90-14.10) | 1.64  (-0.23-3.62) | -4.84  (-5.03--4.65) |
| United Republic of Tanzania | 355.59  (-48.67-770.86) | 1084.60  (-122.05-2358.30) | 4.00  (-0.55-8.64) | 5.30  (-0.60-11.50) | 0.72  (0.50-0.94) |
| United States of America | 22373.89  (-3223.80-48716.08) | 15842.73  (-2225.82-34522.39) | 6.74  (-0.97-14.68) | 2.46  (-0.35-5.37) | -3.55  (-3.70--3.40) |
| United States Virgin Islands | 4.91  (-0.69-11.25) | 5.93  (-0.80-13.19) | 7.48  (-1.05-17.10) | 3.44  (-0.47-7.70) | -2.48  (-2.73--2.22) |
| Uruguay | 232.86  (-32.46-510.31) | 199.95  (-28.82-437.97) | 6.01  (-0.84-13.17) | 3.18  (-0.46-7.00) | -2.18  (-2.27--2.08) |
| Uzbekistan | 1078.85  (-142.75-2388.27) | 2443.33  (-331.92-5341.94) | 10.56  (-1.40-23.32) | 12.94  (-1.77-28.31) | 0.80  (0.34-1.26) |
| Vanuatu | 6.69  (-0.86-14.91) | 17.48  (-2.40-38.80) | 14.24  (-1.91-32.31) | 13.13  (-1.81-29.00) | -0.31  (-0.41--0.21) |
| Venezuela (Bolivarian Republic of) | 1075.37  (-154.46-2274.61) | 3258.26  (-457.32-7346.33) | 12.42  (-1.79-26.28) | 11.53  (-1.63-25.91) | -0.68  (-0.89--0.47) |
| Viet Nam | 1070.58  (-159.99-2470.84) | 3296.47  (-430.89-7390.92) | 2.99  (-0.45-6.91) | 3.98  (-0.53-8.82) | 1.34  (1.12-1.56) |
| Yemen | 1622.59  (-223.88-3309.00) | 3770.33  (-538.48-7889.18) | 39.80  (-5.54-80.21) | 34.55  (-5.11-71.01) | -0.67  (-0.75--0.60) |
| Zambia | 100.64  (-15.33-216.15) | 299.64  (-38.95-661.25) | 4.63  (-0.72-9.91) | 6.07  (-0.82-13.42) | 0.89  (0.66-1.12) |
| Zimbabwe | 182.80  (-27.54-410.34) | 441.56  (-58.00-939.77) | 5.90  (-0.88-13.19) | 8.72  (-1.18-18.79) | 1.76  (1.23-2.30) |

**Table S2.** The DALYs, ASDR and EAPC of Ischemic heart disease due to lead exposure among 204 countries and territories from 1990 to 2021

| **Country** | **DALYs case** | | **ASDR (per 100,000 population)** | | |
| --- | --- | --- | --- | --- | --- |
|  | **1990** | **2021** | **1990** | **2021** | **1990-2021** |
|  | **Both (95% UI)** | **Both (95% UI)** | **Both (95% UI)** | **Both (95% UI)** | **EAPC(95% CI)** |
| Afghanistan | 68239.80  (-9929.37-147167.65) | 74185.67  (-10266.11-161932.26) | 975.97  (-142.90-2086.01) | 758.43  (-108.65-1617.92) | -1.05  (-1.21--0.89) |
| Albania | 2801.30  (-384.02-6091.95) | 5498.31  (-754.13-12384.50) | 144.45  (-19.87-312.50) | 129.12  (-17.67-290.33) | -0.04  (-0.23-0.14) |
| Algeria | 43274.00  (-5671.03-94866.23) | 72170.59  (-9301.06-158075.81) | 413.92  (-55.36-899.30) | 248.79  (-32.69-542.71) | -1.83  (-1.92--1.73) |
| American Samoa | 10.36  (-1.36-23.14) | 23.65  (-3.00-51.79) | 47.67  (-6.26-105.32) | 50.93  (-6.45-111.67) | 0.29  (0.21-0.36) |
| Andorra | 21.52  (-2.94-50.87) | 32.57  (-4.12-72.21) | 39.25  (-5.33-92.49) | 19.82  (-2.50-43.93) | -2.06  (-2.29--1.83) |
| Angola | 6528.36  (-860.37-14378.61) | 16490.60  (-2249.18-35074.30) | 170.87  (-23.66-371.88) | 158.01  (-21.37-335.97) | -0.46  (-0.56--0.36) |
| Antigua and Barbuda | 68.64  (-9.22-147.60) | 63.25  (-9.40-136.92) | 128.44  (-17.20-275.39) | 63.33  (-9.40-136.79) | -2.69  (-2.91--2.48) |
| Argentina | 29867.93  (-4035.59-67109.25) | 23847.58  (-3321.29-53195.66) | 94.28  (-12.73-211.89) | 42.27  (-5.88-93.91) | -2.32  (-2.42--2.23) |
| Armenia | 5212.73  (-684.84-11452.47) | 6565.89  (-905.27-14706.68) | 205.48  (-27.10-452.27) | 151.55  (-20.89-340.40) | -1.64  (-1.92--1.37) |
| Australia | 36563.45  (-5132.51-80058.44) | 19205.33  (-2723.62-41742.61) | 188.89  (-26.49-413.60) | 39.17  (-5.52-85.30) | -5.27  (-5.37--5.17) |
| Austria | 10932.80  (-1561.95-23605.36) | 6948.63  (-992.93-15181.54) | 91.86  (-13.03-198.15) | 33.41  (-4.78-73.12) | -3.69  (-3.94--3.43) |
| Azerbaijan | 11351.10  (-1554.72-24676.34) | 17413.29  (-2284.93-37613.61) | 240.69  (-33.08-516.28) | 200.78  (-26.97-433.18) | -0.58  (-0.90--0.26) |
| Bahamas | 156.02  (-21.70-342.78) | 191.38  (-25.36-427.34) | 100.62  (-13.98-220.53) | 48.69  (-6.50-108.96) | -2.45  (-2.59--2.30) |
| Bahrain | 664.27  (-89.70-1456.53) | 954.83  (-129.06-2100.92) | 403.15  (-54.90-890.24) | 138.90  (-19.28-300.06) | -4.17  (-4.62--3.73) |
| Bangladesh | 154474.31  (-24673.63-335946.15) | 312748.42  (-45796.71-670629.89) | 315.43  (-51.10-680.31) | 233.04  (-34.26-501.41) | -0.78  (-0.92--0.64) |
| Barbados | 288.95  (-39.85-631.43) | 248.26  (-33.44-569.78) | 99.80  (-13.75-218.60) | 47.32  (-6.37-108.72) | -2.72  (-2.98--2.45) |
| Belarus | 20361.54  (-2708.03-45128.36) | 31828.11  (-4228.98-68406.73) | 162.06  (-21.53-358.58) | 196.51  (-26.06-423.18) | 0.13  (-0.42-0.68) |
| Belgium | 26148.96  (-3508.32-56229.30) | 11105.76  (-1587.12-24399.86) | 170.99  (-22.90-367.68) | 43.42  (-6.17-96.16) | -4.50  (-4.73--4.27) |
| Belize | 124.68  (-17.53-272.05) | 180.90  (-24.42-399.24) | 134.79  (-19.00-294.31) | 64.42  (-8.74-142.27) | -3.01  (-3.44--2.58) |
| Benin | 1878.64  (-290.30-4093.27) | 4721.92  (-672.16-10063.83) | 98.30  (-15.20-212.62) | 101.48  (-14.68-213.69) | 0.12  (-0.03-0.28) |
| Bermuda | 90.87  (-12.44-203.21) | 51.44  (-6.47-116.41) | 149.58  (-20.59-333.25) | 35.37  (-4.46-80.17) | -4.60  (-4.85--4.34) |
| Bhutan | 719.15  (-120.48-1604.87) | 1455.80  (-213.28-3026.22) | 288.56  (-49.14-636.94) | 248.83  (-36.66-516.74) | -0.43  (-0.52--0.35) |
| Bolivia (Plurinational State of) | 5568.08  (-727.23-13036.27) | 9506.64  (-1252.60-21086.51) | 181.55  (-23.80-420.33) | 113.24  (-14.98-252.08) | -1.51  (-1.68--1.34) |
| Bosnia and Herzegovina | 9081.85  (-1268.20-19463.50) | 8876.68  (-1277.88-19796.59) | 228.29  (-31.83-486.74) | 139.87  (-20.09-311.87) | -2.05  (-2.26--1.83) |
| Botswana | 950.45  (-134.97-2095.35) | 1529.51  (-183.40-3331.49) | 174.81  (-24.82-386.17) | 114.22  (-13.97-248.75) | -1.66  (-2.09--1.23) |
| Brazil | 163285.44  (-22666.18-350016.43) | 204193.45  (-29845.02-437452.92) | 183.94  (-25.68-394.42) | 81.28  (-11.90-174.31) | -2.53  (-2.62--2.45) |
| Brunei Darussalam | 156.42  (-21.99-339.81) | 198.71  (-28.75-451.14) | 147.15  (-20.70-319.65) | 63.24  (-9.25-140.91) | -2.44  (-2.62--2.27) |
| Bulgaria | 24813.77  (-3494.19-55112.66) | 18625.19  (-2551.81-40666.66) | 235.08  (-33.00-518.64) | 134.18  (-18.41-292.78) | -2.56  (-2.93--2.18) |
| Burkina Faso | 6512.46  (-1047.18-14324.53) | 16490.63  (-2216.52-34550.47) | 159.57  (-25.71-355.05) | 197.10  (-27.27-414.80) | 0.89  (0.69-1.08) |
| Burundi | 4171.07  (-545.16-9169.31) | 6343.07  (-782.95-13559.73) | 183.42  (-23.95-396.90) | 141.89  (-17.58-301.46) | -1.36  (-1.56--1.15) |
| Cabo Verde | 196.71  (-27.27-416.23) | 549.89  (-77.47-1215.72) | 86.08  (-11.86-182.21) | 129.81  (-18.34-283.71) | 0.72  (0.32-1.12) |
| Cambodia | 6538.24  (-946.41-14174.84) | 16714.44  (-2346.44-37183.91) | 142.97  (-20.67-305.65) | 144.48  (-20.40-322.54) | -0.08  (-0.15--0.01) |
| Cameroon | 4734.13  (-668.90-10709.37) | 17966.96  (-2431.80-41339.50) | 112.39  (-16.20-253.37) | 157.64  (-22.21-359.25) | 1.15  (0.45-1.86) |
| Canada | 25968.81  (-3515.61-57570.38) | 18085.70  (-2522.42-40231.81) | 80.53  (-10.91-178.68) | 23.69  (-3.30-52.31) | -4.16  (-4.29--4.02) |
| Central African Republic | 3087.43  (-489.60-6690.68) | 5458.84  (-834.36-11437.84) | 268.74  (-42.17-586.90) | 259.50  (-40.68-545.30) | -0.25  (-0.43--0.08) |
| Chad | 4590.11  (-638.29-9866.07) | 12275.06  (-1691.62-26081.78) | 168.45  (-23.50-361.17) | 231.53  (-32.54-485.20) | 0.91  (0.57-1.25) |
| Chile | 4204.37  (-566.51-9184.19) | 4609.69  (-639.53-9875.35) | 43.91  (-5.91-95.74) | 17.87  (-2.48-38.21) | -2.74  (-2.81--2.67) |
| China | 925397.01  (-128077.01-1997463.71) | 2590027.50  (-360203.04-5650619.85) | 120.30  (-17.02-258.00) | 133.33  (-18.62-291.17) | 0.71  (0.35-1.06) |
| Colombia | 36318.08  (-4962.34-77923.17) | 50993.08  (-7056.84-110080.70) | 217.97  (-29.90-465.87) | 90.99  (-12.58-196.84) | -3.08  (-3.21--2.95) |
| Comoros | 248.64  (-39.52-548.55) | 405.32  (-53.14-846.88) | 132.02  (-20.99-287.72) | 91.48  (-12.17-190.52) | -1.49  (-1.61--1.37) |
| Congo | 2016.11  (-295.78-4312.01) | 3470.46  (-452.20-7697.82) | 192.53  (-28.38-405.33) | 144.66  (-19.37-324.99) | -1.25  (-1.42--1.08) |
| Cook Islands | 6.37  (-0.83-14.06) | 7.50  (-0.98-16.84) | 53.77  (-7.03-120.13) | 29.40  (-3.82-66.07) | -1.90  (-2.00--1.81) |
| Costa Rica | 2874.46  (-412.14-6114.35) | 4093.26  (-569.31-8729.27) | 168.46  (-24.20-358.25) | 73.47  (-10.22-156.50) | -2.78  (-2.99--2.58) |
| Croatia | 9524.03  (-1351.07-21049.29) | 7534.87  (-1047.67-16903.54) | 168.24  (-23.82-372.25) | 79.51  (-10.99-177.61) | -2.49  (-2.62--2.37) |
| Cuba | 30354.45  (-4327.65-66019.44) | 31417.29  (-4528.48-65295.31) | 302.23  (-43.07-656.96) | 155.42  (-22.32-323.37) | -2.39  (-2.60--2.17) |
| Cyprus | 1524.80  (-236.38-3306.97) | 1379.90  (-200.17-3006.33) | 234.06  (-36.00-506.67) | 73.42  (-10.66-158.64) | -4.09  (-4.29--3.89) |
| Czechia | 21381.81  (-2981.49-46139.46) | 12918.09  (-1734.63-28786.93) | 157.11  (-21.87-339.29) | 57.11  (-7.68-127.64) | -3.20  (-3.29--3.11) |
| Côte d'Ivoire | 6369.90  (-823.38-13766.70) | 17319.54  (-2433.47-39007.39) | 168.77  (-22.86-363.20) | 172.92  (-24.91-380.23) | -0.14  (-0.49-0.20) |
| Democratic People's Republic of Korea | 18616.70  (-2469.44-41158.02) | 47896.33  (-6139.46-102931.92) | 121.43  (-16.80-262.64) | 151.53  (-19.53-326.00) | 0.72  (0.44-1.00) |
| Democratic Republic of the Congo | 22898.95  (-3186.75-48787.32) | 50878.10  (-7611.77-111095.17) | 153.50  (-21.45-325.98) | 152.29  (-22.51-329.03) | -0.13  (-0.18--0.08) |
| Denmark | 10040.60  (-1460.28-21955.67) | 2886.20  (-415.95-6322.27) | 124.27  (-18.07-270.93) | 22.95  (-3.28-50.10) | -5.83  (-5.99--5.68) |
| Djibouti | 148.85  (-18.58-336.17) | 728.05  (-85.09-1683.33) | 114.66  (-14.90-255.64) | 129.39  (-15.37-295.36) | 0.26  (0.00-0.52) |
| Dominica | 84.94  (-11.75-187.05) | 62.50  (-8.85-135.91) | 144.91  (-20.13-318.70) | 77.24  (-10.92-167.33) | -2.22  (-2.40--2.03) |
| Dominican Republic | 10686.49(-1622.88-23276.37) | 27563.68(-3591.76-59398.87) | 300.42  (-45.63-642.90) | 278.89  (-36.36-601.33) | 0.28  (0.09-0.47) |
| Ecuador | 4065.34(-537.90-9005.34) | 9612.33(-1297.27-21376.94) | 80.98  (-10.81-178.31) | 62.10  (-8.46-138.43) | -0.95  (-1.42--0.49) |
| Egypt | 208861.63  (-30682.51-447309.90) | 412960.32  (-56838.66-862933.17) | 808.13  (-120.81-1721.25) | 739.80  (-105.59-1550.05) | 0.06  (-0.19-0.32) |
| El Salvador | 8382.93  (-1222.59-17775.87) | 12899.4  8(-1802.89-27351.02) | 284.01  (-41.62-602.03) | 200.91  (-28.01-429.09) | -1.25  (-1.44--1.06) |
| Equatorial Guinea | 386.07  (-61.62-816.36) | 626.14  (-80.45-1458.03) | 200.97  (-31.77-427.38) | 142.74  (-19.15-328.18) | -1.39  (-1.57--1.21) |
| Eritrea | 1635.26  (-208.39-3635.13) | 3001.71  (-374.07-6683.80) | 143.59  (-17.86-310.79) | 121.69  (-15.48-267.61) | -0.67  (-0.85--0.49) |
| Estonia | 2592.11  (-348.55-5587.50) | 1117.43  (-158.75-2462.23) | 130.03  (-17.46-280.65) | 38.21  (-5.40-83.50) | -4.47  (-5.02--3.93) |
| Eswatini | 417.84  (-70.89-913.85) | 841.50  (-129.59-1894.12) | 147.65  (-25.06-319.60) | 154.79  (-23.67-341.07) | 0.68  (-0.05-1.41) |
| Ethiopia | 34031.00  (-4715.91-74784.43) | 41398.50  (-5379.21-90212.93) | 175.98  (-24.88-384.41) | 104.63  (-13.79-228.22) | -1.98  (-2.10--1.86) |
| Fiji | 538.12  (-71.22-1199.59) | 822.47  (-103.27-1846.11) | 149.24  (-19.63-332.43) | 112.18  (-14.26-250.40) | -1.09  (-1.24--0.94) |
| Finland | 4905.83  (-685.32-10906.14) | 3231.51  (-446.17-7240.23) | 69.53  (-9.72-154.86) | 22.91  (-3.15-51.25) | -3.61  (-3.72--3.49) |
| France | 56823.93  (-7935.15-121799.21) | 35633.76  (-5072.53-76350.24) | 67.93  (-9.45-145.52) | 21.58  (-3.05-46.02) | -3.88  (-4.09--3.66) |
| Gabon | 696.90  (-108.24-1524.45) | 944.66  (-122.11-2065.91) | 125.49  (-19.67-274.02) | 101.39  (-13.05-220.93) | -0.79  (-0.99--0.60) |
| Gambia | 596.03  (-86.47-1354.60) | 1987.07  (-278.15-4175.22) | 178.58  (-25.76-403.57) | 221.11  (-31.02-463.19) | 0.52  (0.28-0.76) |
| Georgia | 17402.37  (-2516.65-37407.55) | 8569.09  (-1278.32-18426.83) | 284.62  (-41.08-611.51) | 144.23  (-21.52-310.64) | -3.03  (-3.61--2.45) |
| Germany | 96774.34  (-13477.91-212555.84) | 55649.24  (-7561.61-123768.04) | 76.47  (-10.62-168.35) | 26.26  (-3.54-58.02) | -3.61  (-3.73--3.49) |
| Ghana | 8676.97  (-1188.26-19312.07) | 14267.62  (-2035.59-30499.58) | 144.05  (-19.93-320.73) | 94.59  (-13.54-201.91) | -1.85  (-2.21--1.49) |
| Greece | 20088.03  (-2802.93-43837.35) | 17382.60  (-2490.41-37776.22) | 135.44  (-18.86-296.52) | 69.83  (-9.90-151.46) | -2.29  (-2.65--1.94) |
| Greenland | 32.34  (-4.25-70.55) | 24.57  (-3.29-54.81) | 99.96  (-13.45-218.74) | 37.58  (-5.04-83.60) | -3.22  (-3.32--3.13) |
| Grenada | 171.69  (-23.11-375.02) | 142.07  (-19.82-307.19) | 245.70  (-33.00-536.66) | 129.31  (-18.15-278.66) | -2.47  (-2.73--2.21) |
| Guam | 34.38  (-4.43-74.41) | 78.74  (-10.19-174.30) | 52.05  (-6.85-112.57) | 37.20  (-4.81-82.46) | -0.47  (-0.69--0.26) |
| Guatemala | 10528.81  (-1477.65-22313.00) | 20863.90  (-3028.61-42749.70) | 341.41  (-49.02-708.72) | 209.50  (-30.69-431.50) | -1.62  (-2.07--1.17) |
| Guinea | 4711.35  (-752.31-9920.48) | 10352.35  (-1426.09-21735.64) | 147.36  (-23.62-309.41) | 198.35  (-27.68-410.39) | 1.19  (0.94-1.44) |
| Guinea-Bissau | 959.79  (-144.92-2085.62) | 1730.44  (-231.06-3710.22) | 247.01  (-37.94-532.31) | 260.65  (-34.68-549.62) | 0.20  (0.01-0.39) |
| Guyana | 1495.07  (-204.73-3332.00) | 1380.25  (-187.12-3047.24) | 395.36  (-54.49-879.49) | 222.38  (-30.24-491.39) | -1.60  (-1.90--1.31) |
| Haiti | 20363.82  (-3055.90-42386.67) | 34399.97  (-5121.67-74560.57) | 652.41  (-99.07-1346.75) | 515.52  (-78.02-1101.77) | -0.60  (-0.65--0.56) |
| Honduras | 5277.66  (-732.71-11174.58) | 20884.63  (-2881.88-43751.53) | 271.73  (-38.16-580.57) | 369.14  (-52.07-767.45) | 1.23  (0.98-1.47) |
| Hungary | 24717.08  (-3437.24-53204.09) | 20300.90  (-2759.26-43547.46) | 175.81  (-24.39-379.15) | 100.82  (-13.65-215.19) | -1.98  (-2.17--1.79) |
| Iceland | 331.8  1(-45.09-733.25) | 203.48  (-26.77-447.11) | 114.51  (-15.51-252.46) | 31.87  (-4.18-70.23) | -4.23  (-4.38--4.07) |
| India | 1429301.40  (-214538.98-3082324.13) | 3425902.70  (-481572.18-7212952.83) | 292.71  (-44.20-627.29) | 292.45  (-41.34-613.26) | 0.14  (0.01-0.27) |
| Indonesia | 162818.93  (-23990.74-348155.48) | 406801.70  (-60137.53-907491.39) | 154.75  (-23.18-330.23) | 178.21  (-26.58-386.32) | 0.56  (0.36-0.76) |
| Iran (Islamic Republic of) | 150373.97  (-21903.33-320329.51) | 221538.22  (-32540.67-475576.67) | 610.54  (-89.62-1296.33) | 305.68  (-45.15-652.83) | -2.44  (-2.62--2.26) |
| Iraq | 31586.87  (-4541.71-68864.93) | 72993.48  (-9636.35-161499.24) | 399.29  (-57.23-865.22) | 342.89  (-46.51-762.92) | -0.97  (-1.13--0.81) |
| Ireland | 7427.39  (-1018.61-16305.39) | 2903.19  (-411.18-6455.78) | 183.89  (-25.16-402.88) | 35.27  (-4.98-78.27) | -5.40  (-5.61--5.19) |
| Israel | 4627.12  (-643.80-10112.00) | 2373.59  (-332.19-5196.58) | 97.68  (-13.57-214.06) | 17.99  (-2.51-39.39) | -5.89  (-6.07--5.71) |
| Italy | 85274.65  (-12340.04-182667.56) | 54881.54  (-7712.53-119770.85) | 97.24  (-14.03-208.75) | 31.49  (-4.39-69.14) | -3.81  (-4.00--3.63) |
| Jamaica | 1791.71  (-242.38-3898.92) | 2230.21  (-293.80-4890.62) | 99.65  (-13.45-217.29) | 69.59  (-9.18-152.34) | -0.86  (-1.26--0.45) |
| Japan | 54321.82  (-7626.11-118956.03) | 48549.31  (-6835.20-107662.33) | 33.30  (-4.67-73.17) | 11.67  (-1.63-25.77) | -3.25  (-3.35--3.14) |
| Jordan | 3747.28  (-484.51-8108.76) | 7913.86  (-1101.21-17359.19) | 277.03  (-36.12-602.84) | 117.11  (-16.35-254.58) | -3.32  (-3.69--2.95) |
| Kazakhstan | 18910.15  (-2641.17-41749.91) | 17028.51  (-2399.42-36734.98) | 156.75  (-21.89-346.01) | 111.42  (-15.74-239.30) | -2.33  (-3.07--1.59) |
| Kenya | 4394.43  (-635.57-9885.88) | 13677.16  (-1930.86-31627.58) | 57.94  (-8.48-130.44) | 69.22  (-9.80-160.92) | 0.77  (0.36-1.18) |
| Kiribati | 41.38  (-5.58-92.16) | 83.67  (-10.42-189.43) | 112.06  (-14.86-249.44) | 114.35  (-14.53-254.96) | 0.06  (-0.03-0.15) |
| Kuwait | 1612.95  (-225.28-3585.70) | 3021.41  (-420.41-6529.33) | 242.98  (-34.52-525.70) | 106.56  (-14.95-226.78) | -2.56  (-3.08--2.04) |
| Kyrgyzstan | 5059.35  (-707.49-11168.75) | 8264.36  (-1124.48-18212.99) | 180.25  (-25.21-396.46) | 203.93  (-28.26-451.24) | 0.54  (0.04-1.04) |
| Lao People's Democratic Republic | 7777.30  (-1087.97-17389.18) | 11537.31  (-1673.26-25112.21) | 362.66  (-51.75-804.37) | 261.68  (-38.03-569.39) | -1.19  (-1.26--1.12) |
| Latvia | 4871.08  (-678.21-10527.76) | 3062.94  (-421.73-6603.00) | 138.90  (-19.30-300.56) | 74.06  (-10.21-159.90) | -2.58  (-3.05--2.11) |
| Lebanon | 4819.13  (-653.29-11089.91) | 4850.06  (-640.64-10505.26) | 227.60  (-30.97-525.37) | 76.82  (-10.10-166.74) | -3.53  (-3.76--3.29) |
| Lesotho | 650.12  (-97.29-1431.79) | 1548.57  (-199.18-3598.35) | 79.40  (-12.07-174.17) | 148.36  (-19.69-342.68) | 2.80  (2.19-3.40) |
| Liberia | 1638.36  (-223.56-3538.76) | 3498.17  (-483.99-7580.15) | 148.78  (-20.30-321.14) | 182.16  (-26.03-391.66) | 0.59  (0.35-0.84) |
| Libya | 5210.35  (-731.39-11762.53) | 11392.04  (-1483.99-25267.69) | 272.91  (-38.62-615.78) | 232.89  (-30.25-517.76) | -0.16  (-0.46-0.15) |
| Lithuania | 6414.20  (-854.34-13655.52) | 5229.14  (-753.66-11462.36) | 144.68  (-19.25-307.79) | 84.49  (-12.12-184.28) | -1.80  (-2.18--1.41) |
| Luxembourg | 518.32  (-69.63-1141.15) | 280.77  (-37.49-629.87) | 96.40  (-12.91-212.66) | 24.61  (-3.27-54.95) | -4.62  (-4.82--4.41) |
| Madagascar | 6361.41  (-901.65-13830.74) | 13753.15  (-1656.38-30963.64) | 128.27  (-18.17-281.08) | 133.20  (-15.92-298.07) | -0.22  (-0.44-0.00) |
| Malawi | 4409.09  (-658.14-9577.99) | 9534.75  (-1316.81-20079.62) | 120.27  (-17.99-254.09) | 136.23  (-19.07-283.29) | 0.11  (-0.32-0.54) |
| Malaysia | 16643.30  (-2425.03-37036.92) | 39965.76  (-5585.62-84428.81) | 175.05  (-25.67-386.83) | 143.65  (-20.25-302.30) | -0.73  (-0.92--0.53) |
| Maldives | 191.09  (-27.66-426.26) | 255.32  (-32.71-550.32) | 205.16  (-30.53-446.79) | 79.22  (-10.10-170.46) | -3.55  (-3.71--3.40) |
| Mali | 4997.20  (-761.32-10628.18) | 11568.67  (-1637.30-25313.68) | 133.95  (-20.50-282.42) | 144.98  (-20.92-314.25) | 0.36  (0.28-0.43) |
| Malta | 1475.79  (-225.21-3057.09) | 1096.13  (-162.27-2297.79) | 350.11  (-53.36-724.67) | 107.60  (-15.79-224.91) | -3.75  (-4.01--3.49) |
| Marshall Islands | 23.57  (-3.18-52.30) | 48.39  (-6.79-106.18) | 151.50  (-20.60-332.14) | 144.44  (-20.92-319.28) | 0.00  (-0.17-0.18) |
| Mauritania | 1298.33  (-174.79-2893.07) | 2189.36  (-284.82-4787.95) | 134.82  (-18.20-300.65) | 111.31  (-14.55-241.53) | -0.94  (-1.07--0.81) |
| Mauritius | 1209.57  (-154.84-2688.49) | 1189.72  (-158.84-2639.13) | 163.12  (-21.05-360.97) | 66.42  (-8.85-146.73) | -3.38  (-3.72--3.05) |
| Mexico | 66797.92  (-9315.50-141755.79) | 185184.25  (-27127.67-398258.29) | 169.08  (-23.78-357.76) | 154.78  (-22.74-332.82) | -0.48  (-0.65--0.31) |
| Micronesia (Federated States of) | 70.48  (-9.62-160.19) | 99.44  (-13.78-226.69) | 142.92  (-19.56-324.58) | 132.71  (-18.94-300.01) | -0.18  (-0.28--0.09) |
| Monaco | 38.77  (-5.58-85.67) | 26.81  (-3.83-58.85) | 55.55  (-7.96-122.95) | 25.21  (-3.51-55.63) | -2.72  (-2.87--2.57) |
| Mongolia | 3252.26  (-451.99-6948.20) | 3759.21  (-475.71-8094.44) | 336.99  (-46.90-720.48) | 201.27  (-25.66-434.54) | -2.12  (-2.50--1.75) |
| Montenegro | 549.29  (-76.32-1202.97) | 962.54  (-127.63-2110.49) | 88.75  (-12.33-194.74) | 104.79  (-13.90-230.38) | 0.56  (0.38-0.75) |
| Morocco | 58293.64  (-7778.24-127859.53) | 99804.21  (-12584.70-215518.72) | 414.57  (-55.69-904.33) | 311.92  (-39.70-677.12) | -0.98  (-1.04--0.92) |
| Mozambique | 4052.17  (-648.88-8423.91) | 10152.58  (-1347.38-21452.60) | 73.74  (-11.79-153.16) | 98.53  (-13.44-206.13) | 1.39  (1.08-1.70) |
| Myanmar | 59028.67  (-7639.83-131422.08) | 80322.39  (-10823.31-175305.13) | 251.07  (-33.08-553.03) | 173.87  (-23.61-376.66) | -1.34  (-1.54--1.15) |
| Namibia | 818.79  (-123.31-1788.10) | 1582.96  (-213.21-3509.93) | 129.67  (-19.48-279.87) | 122.32  (-16.48-270.99) | -0.35  (-0.76-0.06) |
| Nauru | 6.95  (-0.82-14.81) | 9.49  (-1.24-21.04) | 150.02  (-17.81-318.15) | 161.61  (-21.67-359.10) | 0.26  (-0.11-0.63) |
| Nepal | 33567.47  (-4952.89-74369.76) | 75195.84  (-10005.68-158040.63) | 351.76  (-51.99-766.95) | 338.97  (-45.30-706.03) | 0.14  (-0.01-0.28) |
| Netherlands | 19758.13  (-2802.00-43262.35) | 7856.75  (-1073.12-17490.42) | 99.52  (-14.05-218.59) | 20.72  (-2.82-46.05) | -5.60  (-5.85--5.35) |
| New Zealand | 7074.04  (-990.20-15176.63) | 4563.33  (-662.29-9858.73) | 182.53  (-25.50-392.23) | 52.33  (-7.56-113.46) | -4.31  (-4.42--4.19) |
| Nicaragua | 2757.97  (-408.23-5722.32) | 5721.31  (-832.14-12035.60) | 193.29  (-28.62-400.76) | 131.26  (-19.22-275.14) | -1.04  (-1.31--0.77) |
| Niger | 3525.33  (-493.16-7868.38) | 12031.13  (-1778.27-26488.95) | 138.82  (-19.35-307.19) | 164.93  (-24.90-353.47) | 0.60  (0.49-0.72) |
| Nigeria | 45721.41  (-6166.13-102617.94) | 89211.10  (-11096.59-196686.07) | 108.56  (-14.64-241.10) | 110.06  (-13.91-241.96) | 0.07  (-0.13-0.28) |
| Niue | 1.90  (-0.25-4.18) | 1.56  (-0.21-3.49) | 85.15  (-11.34-188.08) | 73.22  (-9.72-163.45) | -0.61  (-0.74--0.49) |
| North Macedonia | 2821.23  (-393.40-6225.82) | 3507.43  (-455.41-7903.82) | 153.24  (-21.46-341.09) | 123.77  (-16.29-274.69) | -1.05  (-1.48--0.62) |
| Northern Mariana Islands | 5.57  (-0.75-12.54) | 17.99  (-2.27-40.03) | 32.15  (-4.31-71.10) | 36.12  (-4.59-80.14) | 0.67  (0.52-0.82) |
| Norway | 7910.26  (-1063.48-17212.71) | 2482.81  (-344.16-5494.46) | 116.88  (-15.64-254.90) | 22.65  (-3.13-50.06) | -5.49  (-5.70--5.27) |
| Oman | 3061.38  (-396.01-6906.02) | 3263.11  (-417.39-7284.92) | 456.24  (-60.90-1024.35) | 197.09  (-25.81-444.32) | -2.54  (-2.82--2.26) |
| Pakistan | 165275.03  (-25245.73-361392.50) | 438772.33  (-66522.26-960125.34) | 293.09  (-44.96-640.61) | 370.02  (-56.44-799.58) | 0.68  (0.44-0.91) |
| Palau | 6.12  (-0.82-14.10) | 12.28  (-1.57-26.31) | 65.70  (-8.89-149.00) | 58.14  (-7.56-125.61) | -0.25  (-0.35--0.15) |
| Palestine | 4262.90  (-585.49-9213.23) | 5924.79  (-838.76-12844.82) | 529.33  (-72.89-1138.51) | 282.06  (-40.44-608.58) | -2.18  (-2.53--1.82) |
| Panama | 2070.98  (-301.91-4456.40) | 3170.74  (-440.50-7018.94) | 144.18  (-21.10-309.86) | 70.58  (-9.79-156.23) | -2.22  (-2.44--1.99) |
| Papua New Guinea | 1437.67  (-207.96-3429.78) | 4027.82  (-501.52-9452.73) | 80.59  (-11.69-188.49) | 85.40  (-10.68-195.89) | 0.25  (0.01-0.49) |
| Paraguay | 2595.12  (-353.72-5705.86) | 5274.51  (-683.58-11835.15) | 118.90  (-16.33-259.91) | 93.21  (-12.15-208.29) | -0.59  (-0.73--0.45) |
| Peru | 9321.04  (-1181.31-21395.32) | 17359.71  (-2276.91-38964.88) | 78.88  (-10.04-180.27) | 51.64  (-6.79-115.81) | -1.73  (-2.16--1.29) |
| Philippines | 36428.04  (-5089.86-81278.26) | 102907.50  (-13746.21-228539.32) | 117.43  (-16.36-260.91) | 125.04  (-16.79-275.09) | 0.16  (0.05-0.27) |
| Poland | 98600.74  (-13684.59-213646.59) | 62624.57  (-9182.23-136839.81) | 230.08  (-31.92-497.85) | 83.56  (-12.22-182.30) | -3.54  (-3.65--3.44) |
| Portugal | 20225.22  (-2876.02-44262.52) | 11812.96  (-1645.87-26075.40) | 151.07  (-21.52-329.50) | 45.03  (-6.19-100.39) | -4.40  (-4.67--4.12) |
| Puerto Rico | 3885.66  (-526.47-8572.89) | 2729.11  (-372.15-5978.12) | 110.26  (-14.93-242.88) | 36.27  (-4.94-79.28) | -3.98  (-4.20--3.76) |
| Qatar | 291.47  (-37.14-650.39) | 611.01  (-83.90-1388.66) | 288.18  (-37.63-639.83) | 83.10  (-11.68-183.35) | -4.73  (-5.55--3.91) |
| Republic of Korea | 14965.87  (-1936.59-33197.41) | 19357.42  (-2764.34-44022.63) | 56.19  (-7.38-125.34) | 20.97  (-2.98-47.60) | -3.71  (-4.05--3.37) |
| Republic of Moldova | 7343.09  (-1012.31-15878.32) | 8971.92  (-1235.40-19176.50) | 185.10  (-25.59-402.27) | 149.71  (-20.60-319.64) | -1.32  (-1.79--0.85) |
| Romania | 47851.01  (-6760.37-104448.04) | 40694.27  (-6009.47-89831.72) | 185.65  (-26.06-405.59) | 105.56  (-15.52-232.71) | -2.47  (-2.75--2.18) |
| Russian Federation | 230310.58  (-30903.56-502900.91) | 261686.68  (-33751.51-571204.93) | 134.09  (-18.02-293.20) | 109.33  (-14.09-238.82) | -1.24  (-1.95--0.52) |
| Rwanda | 4117.11  (-579.61-9029.98) | 4671.60  (-563.85-10243.22) | 151.22  (-20.97-331.51) | 84.92  (-10.35-185.11) | -2.86  (-3.22--2.49) |
| Saint Kitts and Nevis | 85.08  (-11.66-185.17) | 51.02  (-6.91-114.82) | 231.48  (-31.64-503.54) | 82.21  (-11.15-182.80) | -3.41  (-3.59--3.23) |
| Saint Lucia | 186.49  (-26.76-396.69) | 154.78  (-21.85-345.75) | 230.69  (-33.23-493.97) | 65.73  (-9.32-146.67) | -4.52  (-4.82--4.21) |
| Saint Vincent and the Grenadines | 217.02  (-31.16-469.47) | 195.64  (-26.65-431.96) | 313.39  (-45.01-677.58) | 146.11  (-20.00-321.70) | -2.68  (-2.89--2.47) |
| Samoa | 73.38  (-9.90-166.13) | 129.00  (-16.60-284.98) | 91.49  (-12.41-206.03) | 93.68  (-12.14-205.70) | 0.12  (0.01-0.22) |
| San Marino | 13.95  (-1.82-29.71) | 9.95  (-1.24-21.97) | 38.87  (-5.08-82.80) | 11.28  (-1.41-25.26) | -3.50  (-3.81--3.18) |
| Sao Tome and Principe | 58.94  (-8.34-127.85) | 131.20  (-16.06-289.75) | 94.50  (-13.41-204.99) | 129.79  (-15.94-284.55) | 1.11  (0.82-1.41) |
| Saudi Arabia | 23988.29  (-3508.32-55621.48) | 55115.13  (-7237.30-119105.62) | 393.90  (-58.38-908.44) | 270.56  (-37.33-579.44) | -1.17  (-1.51--0.82) |
| Senegal | 5296.64  (-788.19-11453.17) | 10140.42  (-1471.83-22885.64) | 174.28  (-26.09-375.31) | 146.13  (-21.82-325.93) | -0.79  (-0.97--0.62) |
| Serbia | 16437.69  (-2309.12-36181.83) | 18049.48  (-2523.95-38709.84) | 166.63  (-23.36-367.36) | 106.67  (-14.87-228.69) | -2.01  (-2.32--1.69) |
| Seychelles | 47.16  (-6.25-104.02) | 52.81(-7.10-116.49) | 84.12  (-11.18-185.46) | 46.75  (-6.33-103.11) | -1.87  (-2.02--1.73) |
| Sierra Leone | 3601.77  (-524.36-8040.57) | 7251.50  (-1060.67-15524.39) | 181.25  (-26.58-400.37) | 205.34  (-30.04-434.84) | 0.62  (0.30-0.94) |
| Singapore | 4401.48  (-610.90-9429.44) | 4120.97  (-573.16-8815.93) | 199.41  (-27.76-425.38) | 48.32  (-6.72-103.61) | -4.77  (-5.06--4.49) |
| Slovakia | 10755.30  (-1530.17-23173.18) | 9066.16  (-1230.40-19969.70) | 182.67  (-25.97-393.24) | 94.72  (-12.84-208.63) | -2.15  (-2.32--1.98) |
| Slovenia | 1572.94  (-213.22-3462.96) | 1117.84  (-146.96-2485.65) | 64.09  (-8.69-140.85) | 23.13  (-3.04-51.20) | -3.84  (-4.05--3.62) |
| Solomon Islands | 386.95  (-52.30-839.67) | 854.22  (-114.77-1900.93) | 313.74  (-42.85-658.99) | 267.69  (-36.35-592.50) | -0.51  (-0.60--0.41) |
| Somalia | 3864.65  (-607.60-8201.33) | 10640.87  (-1749.23-22607.41) | 164.03  (-27.06-343.94) | 180.24  (-29.75-383.87) | 0.33  (0.13-0.53) |
| South Africa | 15369.46  (-1977.16-34636.84) | 31163.08  (-4121.91-67369.87) | 71.61  (-9.29-161.90) | 70.49  (-9.40-152.68) | -0.17  (-0.70-0.36) |
| South Sudan | 3134.79  (-476.44-6698.29) | 4586.79  (-639.38-9898.07) | 125.96  (-19.13-269.30) | 130.01  (-18.38-277.14) | -0.07  (-0.16-0.02) |
| Spain | 53542.79  (-7712.84-116594.06) | 37701.88  (-5351.93-82986.36) | 100.16  (-14.42-219.06) | 34.90  (-4.94-76.39) | -3.67  (-3.90--3.44) |
| Sri Lanka | 8213.83  (-1104.17-18024.35) | 15557.30  (-1914.33-36168.39) | 76.77  (-10.40-167.83) | 58.65  (-7.24-136.51) | -0.62  (-0.98--0.26) |
| Sudan | 75303.20  (-10756.92-162215.19) | 103243.08  (-13706.68-231203.55) | 805.89  (-116.11-1741.82) | 549.93  (-73.04-1217.62) | -1.40  (-1.51--1.29) |
| Suriname | 669.20  (-89.27-1468.01) | 793.68  (-112.99-1789.73) | 264.01  (-35.49-576.25) | 125.80  (-18.07-283.82) | -2.31  (-2.53--2.09) |
| Sweden | 10649.00  (-1475.93-23594.94) | 4661.72  (-652.04-10402.82) | 69.07  (-9.58-152.62) | 19.05  (-2.65-42.06) | -4.08  (-4.24--3.91) |
| Switzerland | 10419.94  (-1402.11-23031.27) | 5350.60  (-745.43-11933.68) | 98.59  (-13.24-218.21) | 25.05  (-3.47-55.54) | -4.63  (-4.77--4.49) |
| Syrian Arab Republic | 37280.12  (-5018.30-79109.60) | 65084.71  (-8873.00-139221.84) | 706.48  (-95.32-1494.71) | 553.91  (-77.34-1160.33) | -1.13  (-1.28--0.98) |
| Taiwan (Province of China) | 11490.17  (-1606.35-25096.78) | 15449.28  (-2219.09-33945.25) | 78.61  (-11.13-173.02) | 35.78  (-5.13-78.53) | -2.46  (-2.60--2.31) |
| Tajikistan | 7507.35  (-1073.54-16787.14) | 11776.75  (-1732.87-25866.08) | 289.04  (-41.19-647.04) | 251.01  (-37.93-548.25) | -0.64  (-1.06--0.22) |
| Thailand | 17244.92  (-2299.93-37855.35) | 33450.68  (-4182.90-75524.68) | 48.76  (-6.61-106.65) | 30.90  (-3.87-69.64) | -2.10  (-2.46--1.74) |
| Timor-Leste | 573.94  (-82.01-1301.48) | 1971.87  (-263.17-4369.92) | 194.31  (-28.36-432.27) | 245.72  (-32.71-539.46) | 1.08  (0.92-1.24) |
| Togo | 1712.71  (-260.86-3578.05) | 5459.53  (-708.76-12018.64) | 146.55  (-22.39-308.30) | 158.03  (-21.09-343.28) | -0.05  (-0.39-0.29) |
| Tokelau | 1.18  (-0.16-2.71) | 1.07  (-0.13-2.38) | 90.73  (-12.54-205.75) | 72.31  (-8.82-161.05) | -0.76  (-0.89--0.62) |
| Tonga | 43.78  (-5.54-97.23) | 51.79  (-6.39-115.59) | 82.79  (-10.56-182.30) | 65.52  (-8.08-145.94) | -0.66  (-0.83--0.50) |
| Trinidad and Tobago | 1249.93  (-168.85-2805.63) | 1264.86  (-162.77-2828.99) | 157.18  (-21.36-352.62) | 65.76  (-8.47-147.27) | -3.46  (-3.75--3.17) |
| Tunisia | 15185.59  (-2261.40-32077.75) | 26431.70  (-3348.09-58589.59) | 323.55  (-48.62-683.36) | 210.74  (-26.65-465.93) | -1.71  (-1.93--1.50) |
| Turkey | 78049.11  (-11354.55-167107.59) | 100502.18  (-13868.93-222513.71) | 229.27  (-33.53-486.08) | 113.46  (-15.75-250.87) | -2.35  (-2.54--2.16) |
| Turkmenistan | 4318.52  (-607.38-9566.07) | 7605.57  (-1037.34-16679.72) | 244.94  (-34.56-542.67) | 214.81  (-29.47-465.57) | -1.34  (-1.83--0.85) |
| Tuvalu | 11.37  (-1.53-25.34) | 14.89  (-2.10-32.51) | 176.51  (-24.39-390.45) | 150.18  (-21.25-326.39) | -0.46  (-0.58--0.34) |
| Uganda | 7135.58  (-1147.37-15336.26) | 14860.01  (-1926.24-32214.89) | 116.23  (-18.84-250.08) | 108.47  (-14.48-230.31) | -0.89  (-1.38--0.40) |
| Ukraine | 80983.82  (-10726.16-178294.73) | 113319.75  (-14088.62-263067.30) | 118.37  (-15.65-260.69) | 142.32  (-17.68-331.61) | -0.01  (-0.68-0.66) |
| United Arab Emirates | 1340.34  (-185.28-3059.49) | 2788.44  (-366.61-6181.31) | 283.89  (-39.60-643.89) | 122.07  (-17.01-268.31) | -1.52  (-2.02--1.01) |
| United Kingdom | 115162.29  (-15931.54-246407.46) | 38373.19  (-5312.99-83628.82) | 128.29  (-17.71-274.34) | 27.99  (-3.86-60.78) | -5.29  (-5.48--5.11) |
| United Republic of Tanzania | 8690.90  (-1186.60-18971.95) | 24598.90  (-2767.26-54247.72) | 83.02  (-11.38-181.43) | 102.79  (-11.66-224.82) | 0.50  (0.27-0.73) |
| United States of America | 399198.83  (-57046.29-863247.77) | 255532.85  (-35555.79-550739.90) | 125.01  (-17.79-270.54) | 42.23  (-5.86-90.76) | -3.80  (-3.94--3.65) |
| United States Virgin Islands | 110.37  (-15.19-254.86) | 100.99  (-13.60-227.51) | 139.69  (-19.42-320.61) | 56.62  (-7.58-126.24) | -2.90  (-3.14--2.66) |
| Uruguay | 4874.44  (-676.87-10706.68) | 3439.35  (-492.57-7565.98) | 127.26  (-17.62-279.47) | 61.28  (-8.72-133.64) | -2.50  (-2.59--2.42) |
| Uzbekistan | 21117.93  (-2782.66-47111.36) | 48506.12  (-6523.46-106263.52) | 193.08  (-25.47-430.58) | 218.68  (-29.68-477.17) | 0.45  (-0.05-0.95) |
| Vanuatu | 181.83  (-22.92-397.63) | 461.40  (-63.08-1031.25) | 297.33  (-38.67-662.13) | 269.92  (-37.11-599.93) | -0.38  (-0.48--0.29) |
| Venezuela (Bolivarian Republic of) | 25313.05  (-3616.24-53429.98) | 65991.68  (-9149.66-148274.74) | 261.19  (-37.53-552.03) | 222.20  (-30.93-498.01) | -1.05  (-1.25--0.85) |
| Viet Nam | 23880.83  (-3543.96-54714.40) | 67787.73  (-8757.11-149857.67) | 60.95  (-9.09-140.22) | 72.66  (-9.48-162.56) | 1.07  (0.82-1.31) |
| Yemen | 42649.35  (-5934.71-85818.03) | 88175.91  (-12205.75-185610.49) | 869.79  (-120.23-1774.59) | 665.77  (-95.10-1395.80) | -1.10  (-1.18--1.02) |
| Zambia | 2406.47  (-353.71-5179.69) | 6888.43  (-881.37-15275.72) | 92.37  (-13.97-197.84) | 113.43  (-14.84-250.38) | 0.58  (0.32-0.84) |
| Zimbabwe | 4239.38  (-638.87-9338.75) | 10931.95  (-1426.80-23221.26) | 114.17  (-17.15-252.11) | 172.30  (-22.84-367.29) | 1.72  (1.12-2.32) |

**Table S3.** Three countries and territories with the top and bottom burden of Ischemic heart disease due to lead exposure in 1990 and 2021

| **Period** | **Measure** | **Death case**  **(Number)** | **DALYs case**  **(Number)** | **ASMR**  **per 100,000 population** | **ASDR**  **per 100,000 population** |
| --- | --- | --- | --- | --- | --- |
| **1990** | **Top three countries** | India  (50980.12) | India  (1429301.4) | Afghanistan  (43.29) | Afghanistan  (975.97) |
|  |  | China  (37934.65) | China  (925397.01) | Egypt  (40.92) | Yemen  (869.79) |
|  |  | United States of America  (22373.89) | United States of America  (399198.83) | Yemen  (39.80) | Egypt  (808.13) |
|  | **Bottom three countries** | Northern Mariana Islands  (0.19) | Northern Mariana Islands  (5.57) | Andorra  (2.11) | Republic of San Marino  (38.87) |
|  |  | Niue  (0,.10) | Niue  (1.90) | Japan  (1.97) | Japan  (33.30) |
|  |  | Tokelau  (0.06) | Tokelau  (1.18) | Northern Mariana Islands  (1.62) | Northern Mariana Islands  (32.15) |
| **2021** | **Top three countries** | China  (151067.57) | India  (3425902.7) | Egyp  t(40.35) | Afghanistan  (758.43) |
|  |  | India  (148675.35) | China  (2590027.50) | Afghanistan  (36.91) | Egypt  (739.80) |
|  |  | Pakistan  (17984.62) | Pakistan  (438772.33) | Yemen  (34.55) | Yemen  (6665.77) |
|  | **Bottom three countries** | Nauru  (0.34) | Cook Islands  (7.50) | Chile  (0.90) | Chile  (17.87) |
|  |  | Niue  (0.08) | Niue  (1.56) | Japan  (0.68) | Japan  (11.67) |
|  |  | Tokelau  (0.06) | Tokelau  (1.07) | San Marino  (0.67) | Republic of San Marino  (11.28) |

**Table S4.** Decomposition analysis of trends in deaths at six regional levels from 1990 to 2021.

| **Location** | **Sex** | **Cause** | **Overll difference** | **Aging** | **Population** | **Epidemiological change** |
| --- | --- | --- | --- | --- | --- | --- |
| High-middle SDI | Male | Ischemic heart disease | 33467.71 | 21106.06 (63.06%) | 21303.54 (63.65%) | -8941.88 (-26.72%) |
| High-middle SDI | Female | Ischemic heart disease | 26708.17 | 15638.06 (58.55%) | 16088.48 (60.24%) | -5018.37 (-18.79%) |
| High-middle SDI | Both | Ischemic heart disease | 60175.88 | 34846.87 (57.91%) | 37228.68 (61.87%) | -11899.66 (-19.77%) |
| High SDI | Male | Ischemic heart disease | -5786.98 | 17370.45 (-300.16%) | 13655.31 (-235.97%) | -36812.74 (636.13%) |
| High SDI | Female | Ischemic heart disease | -4965.86 | 11119.57 (-223.92%) | 8159.79 (-164.32%) | -24245.21 (488.24%) |
| High SDI | Both | Ischemic heart disease | -10752.83 | 25859.41 (-240.49%) | 21305.67 (-198.14%) | -57917.91 (538.63%) |
| Low SDI | Male | Ischemic heart disease | 17054.23 | -1249.98 (-7.33%) | 18086.31 (106.05%) | 217.9 (1.28%) |
| Low SDI | Female | Ischemic heart disease | 9922.97 | 22.48 (0.23%) | 11235.9 (113.23%) | -1335.42 (-13.46%) |
| Low SDI | Both | Ischemic heart disease | 26977.2 | -1010.17 (-3.74%) | 29436.38 (109.12%) | -1449.02 (-5.37%) |
| Global | Male | Ischemic heart disease | 193720.92 | 79724.2 (41.15%) | 146093.2 (75.41%) | -32096.48 (-16.57%) |
| Global | Female | Ischemic heart disease | 118967.17 | 47407.62 (39.85%) | 97281.9 (81.77%) | -25722.34 (-21.62%) |
| Global | Both | Ischemic heart disease | 312688.09 | 121348.27 (38.81%) | 243713.95 (77.94%) | -52374.13 (-16.75%) |
| Middle SDI | Male | Ischemic heart disease | 85084.58 | 33433.19 (39.29%) | 49323.61 (57.97%) | 2327.78 (2.74%) |
| Middle SDI | Female | Ischemic heart disease | 49280.12 | 22129.19 (44.9%) | 32298.34 (65.54%) | -5147.42 (-10.45%) |
| Middle SDI | Both | Ischemic heart disease | 134364.69 | 55199.14 (41.08%) | 82298.79 (61.25%) | -3133.23 (-2.33%) |
| Low-middle SDI | Male | Ischemic heart disease | 63845.21 | 9160.49 (14.35%) | 48872.82 (76.55%) | 5811.9 (9.1%) |
| Low-middle SDI | Female | Ischemic heart disease | 37957.13 | 9606.17 (25.31%) | 31885.85 (84%) | -3534.89 (-9.31%) |
| Low-middle SDI | Both | Ischemic heart disease | 101802.34 | 19725.27 (19.38%) | 81420.43 (79.98%) | 656.64 (0.65%) |

**Table S5.** Decomposition analysis of trends in DALYs at six regional levels from 1990 to 2021.

| **Location** | **Sex** | **Cause** | **Overll difference** | **Aging** | **Population** | **Epidemiological change** |
| --- | --- | --- | --- | --- | --- | --- |
| Global | Male | Ischemic heart disease | 3511307.5 | 1320039.38 (37.59%) | 3377443.86 (96.19%) | -1186175.74 (-33.78%) |
| Global | Female | Ischemic heart disease | 1858900.01 | 674711.63 (36.3%) | 1874254.19 (100.83%) | -690065.82 (-37.12%) |
| Global | Both | Ischemic heart disease | 5370207.51 | 1913374.93 (35.63%) | 5264080.09 (98.02%) | -1807247.51 (-33.65%) |
| High-middle SDI | Male | Ischemic heart disease | 452531.58 | 331747.42 (73.31%) | 454733.21 (100.49%) | -333949.06 (-73.8%) |
| High-middle SDI | Female | Ischemic heart disease | 314369.94 | 195304.05 (62.13%) | 264301.22 (84.07%) | -145235.33 (-46.2%) |
| High-middle SDI | Both | Ischemic heart disease | 766901.52 | 497334.33 (64.85%) | 713911.78 (93.09%) | -444344.59 (-57.94%) |
| High SDI | Male | Ischemic heart disease | -219496.96 | 247601.23 (-112.8%) | 265915.72 (-121.15%) | -733013.92 (333.95%) |
| High SDI | Female | Ischemic heart disease | -116733.89 | 122731.45 (-105.14%) | 118094.74 (-101.17%) | -357560.08 (306.3%) |
| High SDI | Both | Ischemic heart disease | -336230.86 | 333508.81 (-99.19%) | 372029.83 (-110.65%) | -1041769.5 (309.84%) |
| Low-middle SDI | Male | Ischemic heart disease | 1377130.2 | 164597.07 (11.95%) | 1249108.02 (90.7%) | -36574.89 (-2.66%) |
| Low-middle SDI | Female | Ischemic heart disease | 711603.91 | 160970.68 (22.62%) | 732372.45 (102.92%) | -181739.23 (-25.54%) |
| Low-middle SDI | Both | Ischemic heart disease | 2088734.11 | 344714.18 (16.5%) | 2002067.56 (95.85%) | -258047.62 (-12.35%) |
| Middle SDI | Male | Ischemic heart disease | 1524190.27 | 581961.6 (38.18%) | 1142138.94 (74.93%) | -199910.26 (-13.12%) |
| Middle SDI | Female | Ischemic heart disease | 752812.59 | 339264.65 (45.07%) | 641405.19 (85.2%) | -227857.25 (-30.27%) |
| Middle SDI | Both | Ischemic heart disease | 2277002.87 | 916836.51 (40.27%) | 1803325.67 (79.2%) | -443159.32 (-19.46%) |
| Low SDI | Male | Ischemic heart disease | 376449.24 | -38578.74 (-10.25%) | 461165.25 (122.5%) | -46137.27 (-12.26%) |
| Low SDI | Female | Ischemic heart disease | 196105.08 | -6009.32 (-3.06%) | 265075.58 (135.17%) | -62961.18 (-32.11%) |
| Low SDI | Both | Ischemic heart disease | 572554.32 | -39322.62 (-6.87%) | 729592.21 (127.43%) | -117715.28 (-20.56%) |

**Table S6**. Frontier deaths, and effective difference by country or territory.

| **Location** | **Risk** | **SDI** | **Rate of Deaths** | **Frontier Deaths** | **Effective difference** |
| --- | --- | --- | --- | --- | --- |
| Afghanistan | Lead exposure | 0.34 | 36.91(-5.56 to 78.22) | 3.11 | 33.8 |
| Albania | Lead exposure | 0.71 | 8.33(-1.15 to 18.8) | 1.42 | 6.91 |
| Algeria | Lead exposure | 0.66 | 16.8(-2.27 to 36.38) | 1.55 | 15.25 |
| American Samoa | Lead exposure | 0.72 | 2.57(-0.32 to 5.7) | 1.21 | 1.36 |
| Andorra | Lead exposure | 0.87 | 1.2(-0.15 to 2.69) | 0.74 | 0.46 |
| Angola | Lead exposure | 0.45 | 8.27(-1.13 to 17.88) | 2.93 | 5.34 |
| Antigua and Barbuda | Lead exposure | 0.75 | 3.72(-0.55 to 8.03) | 1.15 | 2.57 |
| Argentina | Lead exposure | 0.72 | 2.25(-0.31 to 5.07) | 1.19 | 1.06 |
| Armenia | Lead exposure | 0.70 | 9.05(-1.25 to 20.62) | 1.45 | 7.61 |
| Australia | Lead exposure | 0.84 | 2.5(-0.36 to 5.45) | 0.91 | 1.59 |
| Austria | Lead exposure | 0.85 | 2.21(-0.31 to 4.83) | 0.9 | 1.31 |
| Azerbaijan | Lead exposure | 0.69 | 12.03(-1.66 to 26) | 1.38 | 10.65 |
| Bahamas | Lead exposure | 0.81 | 2.56(-0.34 to 5.75) | 0.91 | 1.65 |
| Bahrain | Lead exposure | 0.75 | 8.67(-1.21 to 18.56) | 1.08 | 7.59 |
| Bangladesh | Lead exposure | 0.49 | 11.72(-1.74 to 25.21) | 2.94 | 8.78 |
| Barbados | Lead exposure | 0.75 | 2.82(-0.39 to 6.42) | 1.11 | 1.71 |
| Belarus | Lead exposure | 0.78 | 10.94(-1.46 to 23.55) | 0.91 | 10.03 |
| Belgium | Lead exposure | 0.85 | 2.56(-0.36 to 5.58) | 0.89 | 1.67 |
| Belize | Lead exposure | 0.61 | 3.52(-0.47 to 7.76) | 2.33 | 1.19 |
| Benin | Lead exposure | 0.37 | 5.49(-0.82 to 11.51) | 2.97 | 2.52 |
| Bermuda | Lead exposure | 0.82 | 2.11(-0.26 to 4.75) | 0.95 | 1.17 |
| Bhutan | Lead exposure | 0.47 | 13.28(-2.03 to 27.4) | 2.95 | 10.33 |
| Bolivia (Plurinational State of) | Lead exposure | 0.60 | 6.4(-0.85 to 14.37) | 2.29 | 4.11 |
| Bosnia and Herzegovina | Lead exposure | 0.72 | 8.27(-1.18 to 18.4) | 1.19 | 7.08 |
| Botswana | Lead exposure | 0.64 | 5.95(-0.75 to 12.81) | 1.91 | 4.04 |
| Brazil | Lead exposure | 0.65 | 3.95(-0.58 to 8.5) | 1.69 | 2.26 |
| Brunei Darussalam | Lead exposure | 0.81 | 3.59(-0.53 to 7.82) | 0.91 | 2.68 |
| Bulgaria | Lead exposure | 0.77 | 7.61(-1.03 to 16.6) | 0.99 | 6.62 |
| Burkina Faso | Lead exposure | 0.29 | 10.61(-1.53 to 22.11) | 3.7 | 6.91 |
| Burundi | Lead exposure | 0.29 | 7.31(-0.91 to 15.41) | 3.71 | 3.6 |
| Cabo Verde | Lead exposure | 0.53 | 7.47(-1.07 to 16.35) | 2.38 | 5.08 |
| Cambodia | Lead exposure | 0.47 | 7.58(-1.06 to 17.08) | 2.96 | 4.62 |
| Cameroon | Lead exposure | 0.48 | 8.3(-1.21 to 18.62) | 2.94 | 5.36 |
| Canada | Lead exposure | 0.87 | 1.42(-0.2 to 3.2) | 0.75 | 0.67 |
| Central African Republic | Lead exposure | 0.31 | 12.47(-1.98 to 26.64) | 3.68 | 8.79 |
| Chad | Lead exposure | 0.24 | 11.91(-1.7 to 24.59) | 3.78 | 8.13 |
| Chile | Lead exposure | 0.77 | 0.9(-0.12 to 1.95) | 0.9 | 0 |
| China | Lead exposure | 0.72 | 8.55(-1.21 to 18.41) | 1.2 | 7.36 |
| Colombia | Lead exposure | 0.66 | 5.44(-0.77 to 11.66) | 1.64 | 3.8 |
| Comoros | Lead exposure | 0.48 | 5.01(-0.68 to 10.56) | 2.95 | 2.06 |
| Congo | Lead exposure | 0.58 | 7.67(-1.03 to 17.02) | 2.38 | 5.29 |
| Cook Islands | Lead exposure | 0.78 | 1.56(-0.21 to 3.48) | 0.92 | 0.64 |
| Costa Rica | Lead exposure | 0.70 | 3.92(-0.55 to 8.56) | 1.37 | 2.54 |
| Coted'Ivoire | Lead exposure | 0.43 | 9.18(-1.33 to 20.1) | 2.93 | 6.26 |
| Croatia | Lead exposure | 0.80 | 5.23(-0.73 to 11.66) | 0.94 | 4.29 |
| Cuba | Lead exposure | 0.67 | 8.65(-1.25 to 18.1) | 1.49 | 7.16 |
| Cyprus | Lead exposure | 0.84 | 4.94(-0.73 to 10.68) | 0.92 | 4.02 |
| Czechia | Lead exposure | 0.83 | 3.6(-0.48 to 8) | 0.91 | 2.69 |
| Democratic People's Republic of Korea | Lead exposure | 0.57 | 8.03(-1.07 to 17.61) | 2.39 | 5.63 |
| Democratic Republic of the Congo | Lead exposure | 0.38 | 7.77(-1.14 to 16.94) | 2.97 | 4.8 |
| Denmark | Lead exposure | 0.90 | 1.39(-0.2 to 3.1) | 0.71 | 0.68 |
| Djibouti | Lead exposure | 0.49 | 6.82(-0.82 to 15.11) | 3.01 | 3.81 |
| Dominica | Lead exposure | 0.75 | 4.51(-0.64 to 9.7) | 1.11 | 3.4 |
| Dominican Republic | Lead exposure | 0.62 | 14.55(-1.89 to 31.11) | 2.05 | 12.5 |
| Ecuador | Lead exposure | 0.66 | 3.82(-0.53 to 8.43) | 1.55 | 2.27 |
| Egypt | Lead exposure | 0.61 | 40.35(-5.93 to 85.13) | 2.27 | 38.08 |
| El Salvador | Lead exposure | 0.56 | 11.12(-1.58 to 23.15) | 2.37 | 8.75 |
| Equatorial Guinea | Lead exposure | 0.66 | 7.86(-1.1 to 17.71) | 1.69 | 6.17 |
| Eritrea | Lead exposure | 0.40 | 6.34(-0.82 to 13.83) | 2.97 | 3.37 |
| Estonia | Lead exposure | 0.84 | 2.29(-0.33 to 5.11) | 0.91 | 1.39 |
| Eswatini | Lead exposure | 0.59 | 7.31(-1.11 to 15.85) | 2.38 | 4.93 |
| Ethiopia | Lead exposure | 0.36 | 5.59(-0.74 to 12.1) | 3 | 2.59 |
| Fiji | Lead exposure | 0.68 | 5.67(-0.73 to 12.39) | 1.5 | 4.17 |
| Finland | Lead exposure | 0.86 | 1.43(-0.2 to 3.24) | 0.87 | 0.56 |
| France | Lead exposure | 0.84 | 1.3(-0.18 to 2.83) | 0.91 | 0.39 |
| Gabon | Lead exposure | 0.63 | 5.54(-0.71 to 12.37) | 1.87 | 3.67 |
| Gambia | Lead exposure | 0.41 | 11.78(-1.64 to 24.75) | 2.95 | 8.83 |
| Georgia | Lead exposure | 0.73 | 7.08(-1.05 to 15.31) | 1.35 | 5.74 |
| Germany | Lead exposure | 0.90 | 1.58(-0.22 to 3.55) | 0.7 | 0.88 |
| Ghana | Lead exposure | 0.56 | 5.07(-0.74 to 10.86) | 2.39 | 2.68 |
| Greece | Lead exposure | 0.79 | 3.83(-0.55 to 8.32) | 0.94 | 2.89 |
| Greenland | Lead exposure | 0.83 | 2.06(-0.28 to 4.64) | 0.98 | 1.07 |
| Grenada | Lead exposure | 0.67 | 6.96(-0.98 to 14.94) | 1.55 | 5.42 |
| Guam | Lead exposure | 0.80 | 1.59(-0.21 to 3.58) | 0.91 | 0.68 |
| Guatemala | Lead exposure | 0.54 | 13.12(-1.96 to 27.44) | 2.37 | 10.75 |
| Guinea | Lead exposure | 0.34 | 10.48(-1.48 to 21.5) | 3.04 | 7.44 |
| Guinea-Bissau | Lead exposure | 0.35 | 13.21(-1.74 to 27.68) | 3.15 | 10.07 |
| Guyana | Lead exposure | 0.65 | 11.32(-1.54 to 24.75) | 1.7 | 9.62 |
| Haiti | Lead exposure | 0.45 | 26.63(-4.09 to 56.53) | 2.96 | 23.68 |
| Honduras | Lead exposure | 0.51 | 21.56(-3.15 to 44.76) | 2.43 | 19.13 |
| Hungary | Lead exposure | 0.79 | 5.73(-0.78 to 12.46) | 0.93 | 4.81 |
| Iceland | Lead exposure | 0.88 | 2.09(-0.27 to 4.62) | 0.74 | 1.35 |
| India | Lead exposure | 0.58 | 14.18(-2.03 to 29.39) | 2.37 | 11.81 |
| Indonesia | Lead exposure | 0.66 | 9.15(-1.38 to 19.42) | 1.63 | 7.52 |
| Iran (Islamic Republic of) | Lead exposure | 0.70 | 17.63(-2.63 to 37.38) | 1.45 | 16.18 |
| Iraq | Lead exposure | 0.66 | 19.04(-2.61 to 42.74) | 1.54 | 17.5 |
| Ireland | Lead exposure | 0.87 | 2.2(-0.31 to 4.96) | 0.74 | 1.46 |
| Israel | Lead exposure | 0.81 | 1.13(-0.16 to 2.5) | 0.94 | 0.19 |
| Italy | Lead exposure | 0.81 | 2.09(-0.29 to 4.61) | 0.94 | 1.15 |
| Jamaica | Lead exposure | 0.68 | 3.82(-0.5 to 8.33) | 1.48 | 2.34 |
| Japan | Lead exposure | 0.87 | 0.68(-0.1 to 1.51) | 0.68 | 0 |
| Jordan | Lead exposure | 0.73 | 6.48(-0.91 to 13.97) | 1.35 | 5.13 |
| Kazakhstan | Lead exposure | 0.73 | 7.03(-0.99 to 15.11) | 1.33 | 5.69 |
| Kenya | Lead exposure | 0.52 | 3.8(-0.54 to 8.95) | 2.38 | 1.43 |
| Kiribati | Lead exposure | 0.53 | 5.34(-0.68 to 11.83) | 2.39 | 2.95 |
| Kuwait | Lead exposure | 0.85 | 5.76(-0.82 to 12.5) | 0.94 | 4.81 |
| Kyrgyzstan | Lead exposure | 0.60 | 12.57(-1.77 to 27.94) | 2.3 | 10.27 |
| Lao People's Democratic Republic | Lead exposure | 0.49 | 13.49(-1.93 to 29.1) | 2.96 | 10.52 |
| Latvia | Lead exposure | 0.83 | 4.13(-0.56 to 8.95) | 1.08 | 3.05 |
| Lebanon | Lead exposure | 0.74 | 4.58(-0.61 to 9.98) | 1.15 | 3.43 |
| Lesotho | Lead exposure | 0.51 | 7(-0.97 to 15.84) | 2.4 | 4.61 |
| Liberia | Lead exposure | 0.35 | 9.69(-1.38 to 20.68) | 3.16 | 6.53 |
| Libya | Lead exposure | 0.73 | 12.61(-1.62 to 28.23) | 1.26 | 11.35 |
| Lithuania | Lead exposure | 0.86 | 5.1(-0.73 to 11.24) | 0.87 | 4.23 |
| Luxembourg | Lead exposure | 0.88 | 1.55(-0.21 to 3.5) | 0.72 | 0.83 |
| Madagascar | Lead exposure | 0.40 | 6.82(-0.8 to 15.37) | 2.94 | 3.88 |
| Malawi | Lead exposure | 0.38 | 6.76(-0.95 to 13.97) | 3.14 | 3.62 |
| Malaysia | Lead exposure | 0.74 | 7.09(-1.02 to 14.8) | 1.15 | 5.94 |
| Maldives | Lead exposure | 0.65 | 4.57(-0.58 to 9.83) | 1.76 | 2.81 |
| Mali | Lead exposure | 0.27 | 7.6(-1.11 to 16.24) | 3.76 | 3.84 |
| Malta | Lead exposure | 0.80 | 6.32(-0.94 to 13.29) | 0.94 | 5.38 |
| Marshall Islands | Lead exposure | 0.57 | 7.21(-1.08 to 16) | 2.37 | 4.84 |
| Mauritania | Lead exposure | 0.50 | 6.24(-0.82 to 13.59) | 2.93 | 3.31 |
| Mauritius | Lead exposure | 0.72 | 3.36(-0.45 to 7.37) | 1.25 | 2.11 |
| Mexico | Lead exposure | 0.66 | 9.35(-1.38 to 20.31) | 1.55 | 7.8 |
| Micronesia (Federated States of) | Lead exposure | 0.59 | 6.21(-0.88 to 13.86) | 2.39 | 3.81 |
| Monaco | Lead exposure | 0.91 | 1.51(-0.22 to 3.36) | 0.68 | 0.83 |
| Mongolia | Lead exposure | 0.62 | 12.49(-1.59 to 27.05) | 2.05 | 10.45 |
| Montenegro | Lead exposure | 0.80 | 6.58(-0.88 to 14.6) | 0.92 | 5.67 |
| Morocco | Lead exposure | 0.56 | 17.6(-2.28 to 37.56) | 2.37 | 15.23 |
| Mozambique | Lead exposure | 0.33 | 4.9(-0.68 to 10.2) | 3.68 | 1.22 |
| Myanmar | Lead exposure | 0.53 | 9.18(-1.27 to 19.73) | 2.38 | 6.8 |
| Namibia | Lead exposure | 0.62 | 6.22(-0.83 to 13.6) | 2.08 | 4.14 |
| Nauru | Lead exposure | 0.63 | 7.49(-1.02 to 16.69) | 2.02 | 5.47 |
| Nepal | Lead exposure | 0.43 | 17.54(-2.37 to 36.07) | 2.95 | 14.6 |
| Netherlands | Lead exposure | 0.89 | 1.33(-0.18 to 2.99) | 0.68 | 0.65 |
| New Zealand | Lead exposure | 0.85 | 3.15(-0.46 to 6.81) | 0.92 | 2.23 |
| Nicaragua | Lead exposure | 0.52 | 8.3(-1.23 to 17.38) | 2.39 | 5.91 |
| Niger | Lead exposure | 0.17 | 8.81(-1.35 to 18.41) | 6.63 | 2.18 |
| Nigeria | Lead exposure | 0.50 | 6.16(-0.79 to 13.26) | 2.94 | 3.22 |
| Niue | Lead exposure | 0.73 | 3.85(-0.52 to 8.51) | 1.28 | 2.57 |
| North Macedonia | Lead exposure | 0.75 | 7.96(-1.06 to 17.65) | 1.12 | 6.85 |
| Northern Mariana Islands | Lead exposure | 0.77 | 1.86(-0.24 to 4.17) | 0.91 | 0.95 |
| Norway | Lead exposure | 0.92 | 1.42(-0.2 to 3.09) | 0.69 | 0.72 |
| Oman | Lead exposure | 0.77 | 11.35(-1.52 to 25.69) | 0.92 | 10.43 |
| Pakistan | Lead exposure | 0.50 | 18.41(-2.82 to 39.44) | 2.94 | 15.47 |
| Palau | Lead exposure | 0.75 | 3.03(-0.4 to 6.56) | 1.04 | 1.99 |
| Palestine | Lead exposure | 0.63 | 17.33(-2.5 to 37.44) | 1.99 | 15.33 |
| Panama | Lead exposure | 0.71 | 3.93(-0.55 to 8.76) | 1.37 | 2.56 |
| Papua New Guinea | Lead exposure | 0.42 | 4.36(-0.56 to 10.14) | 2.94 | 1.42 |
| Paraguay | Lead exposure | 0.64 | 4.98(-0.66 to 11.1) | 2 | 2.99 |
| Peru | Lead exposure | 0.66 | 2.73(-0.36 to 6.11) | 1.56 | 1.17 |
| Philippines | Lead exposure | 0.65 | 5.93(-0.81 to 13.06) | 1.67 | 4.26 |
| Poland | Lead exposure | 0.81 | 4.93(-0.72 to 10.91) | 0.94 | 4 |
| Portugal | Lead exposure | 0.74 | 2.55(-0.35 to 5.61) | 1.14 | 1.41 |
| Puerto Rico | Lead exposure | 0.83 | 1.9(-0.26 to 4.19) | 0.91 | 0.99 |
| Qatar | Lead exposure | 0.85 | 5.1(-0.72 to 11.18) | 0.91 | 4.19 |
| Republic of Korea | Lead exposure | 0.89 | 1.43(-0.2 to 3.26) | 0.72 | 0.71 |
| Republic of Moldova | Lead exposure | 0.73 | 8.04(-1.1 to 17.03) | 1.16 | 6.88 |
| Romania | Lead exposure | 0.77 | 6.11(-0.9 to 13.62) | 1.02 | 5.09 |
| Russian Federation | Lead exposure | 0.81 | 5.83(-0.76 to 12.8) | 0.93 | 4.9 |
| Rwanda | Lead exposure | 0.44 | 4.67(-0.58 to 10.1) | 3 | 1.67 |
| Saint Kitts and Nevis | Lead exposure | 0.75 | 4.74(-0.65 to 10.56) | 1.05 | 3.69 |
| Saint Lucia | Lead exposure | 0.67 | 3.9(-0.56 to 8.79) | 1.49 | 2.41 |
| Saint Vincent and the Grenadines | Lead exposure | 0.64 | 9.14(-1.27 to 19.94) | 1.9 | 7.24 |
| Samoa | Lead exposure | 0.59 | 4.88(-0.64 to 10.68) | 2.33 | 2.55 |
| San Marino | Lead exposure | 0.89 | 0.67(-0.08 to 1.48) | 0.67 | 0 |
| Sao Tome and Principe | Lead exposure | 0.51 | 7.09(-0.88 to 15.32) | 2.99 | 4.1 |
| Saudi Arabia | Lead exposure | 0.82 | 13.31(-1.91 to 28.29) | 0.98 | 12.33 |
| Senegal | Lead exposure | 0.41 | 8.21(-1.27 to 17.91) | 2.99 | 5.22 |
| Serbia | Lead exposure | 0.79 | 6.54(-0.92 to 14.09) | 0.91 | 5.62 |
| Seychelles | Lead exposure | 0.73 | 2.45(-0.33 to 5.44) | 1.18 | 1.28 |
| Sierra Leone | Lead exposure | 0.36 | 10.7(-1.55 to 22.55) | 2.97 | 7.73 |
| Singapore | Lead exposure | 0.86 | 2.73(-0.38 to 5.92) | 0.85 | 1.89 |
| Slovakia | Lead exposure | 0.81 | 5.84(-0.8 to 12.96) | 0.92 | 4.92 |
| Slovenia | Lead exposure | 0.84 | 1.34(-0.17 to 3.01) | 0.92 | 0.42 |
| Solomon Islands | Lead exposure | 0.43 | 13.93(-1.87 to 31.37) | 2.94 | 10.99 |
| Somalia | Lead exposure | 0.08 | 8.48(-1.39 to 18.32) | 7.46 | 1.02 |
| South Africa | Lead exposure | 0.68 | 3.66(-0.49 to 7.99) | 1.51 | 2.15 |
| South Sudan | Lead exposure | 0.28 | 6.59(-0.94 to 13.72) | 3.7 | 2.89 |
| Spain | Lead exposure | 0.77 | 2.02(-0.29 to 4.41) | 1.01 | 1.02 |
| Sri Lanka | Lead exposure | 0.70 | 2.96(-0.37 to 6.93) | 1.38 | 1.59 |
| Sudan | Lead exposure | 0.54 | 28.41(-3.76 to 61.78) | 2.37 | 26.03 |
| Suriname | Lead exposure | 0.63 | 6.31(-0.94 to 14.44) | 2 | 4.31 |
| Sweden | Lead exposure | 0.89 | 1.2(-0.16 to 2.65) | 0.71 | 0.49 |
| Switzerland | Lead exposure | 0.93 | 1.68(-0.23 to 3.75) | 0.72 | 0.96 |
| Syrian Arab Republic | Lead exposure | 0.62 | 32.49(-4.67 to 67.66) | 2.05 | 30.44 |
| Taiwan (Province of China) | Lead exposure | 0.87 | 1.94(-0.28 to 4.28) | 0.71 | 1.23 |
| Tajikistan | Lead exposure | 0.54 | 15.06(-2.32 to 32.67) | 2.38 | 12.68 |
| Thailand | Lead exposure | 0.68 | 1.52(-0.19 to 3.51) | 1.48 | 0.05 |
| Timor-Leste | Lead exposure | 0.44 | 13.06(-1.74 to 28.14) | 2.96 | 10.09 |
| Togo | Lead exposure | 0.41 | 8.26(-1.13 to 17.62) | 3.03 | 5.23 |
| Tokelau | Lead exposure | 0.69 | 3.89(-0.48 to 8.78) | 1.45 | 2.44 |
| Tonga | Lead exposure | 0.63 | 3.41(-0.42 to 7.57) | 2.03 | 1.38 |
| Trinidad and Tobago | Lead exposure | 0.77 | 3.39(-0.44 to 7.74) | 1.01 | 2.39 |
| Tunisia | Lead exposure | 0.68 | 12.49(-1.6 to 27.39) | 1.48 | 11.01 |
| Turkey | Lead exposure | 0.71 | 6.91(-0.97 to 15.18) | 1.37 | 5.53 |
| Turkmenistan | Lead exposure | 0.68 | 12.3(-1.7 to 26.6) | 1.48 | 10.82 |
| Tuvalu | Lead exposure | 0.58 | 7.61(-1.09 to 16.53) | 2.37 | 5.24 |
| Uganda | Lead exposure | 0.42 | 5.58(-0.78 to 11.83) | 2.93 | 2.65 |
| Ukraine | Lead exposure | 0.76 | 8.67(-1.09 to 19.74) | 1.07 | 7.6 |
| United Arab Emirates | Lead exposure | 0.85 | 7.56(-1.07 to 16.75) | 0.91 | 6.65 |
| United Kingdom | Lead exposure | 0.86 | 1.64(-0.23 to 3.62) | 0.81 | 0.83 |
| United Republic of Tanzania | Lead exposure | 0.45 | 5.3(-0.6 to 11.5) | 3.01 | 2.29 |
| United States Virgin Islands | Lead exposure | 0.82 | 3.44(-0.47 to 7.7) | 0.91 | 2.54 |
| United States of America | Lead exposure | 0.86 | 2.46(-0.35 to 5.37) | 0.8 | 1.67 |
| Uruguay | Lead exposure | 0.72 | 3.18(-0.46 to 7) | 1.2 | 1.98 |
| Uzbekistan | Lead exposure | 0.66 | 12.94(-1.77 to 28.31) | 1.55 | 11.4 |
| Vanuatu | Lead exposure | 0.47 | 13.13(-1.81 to 29) | 2.94 | 10.2 |
| Venezuela (Bolivarian Republic of) | Lead exposure | 0.60 | 11.53(-1.63 to 25.91) | 2.34 | 9.2 |
| Viet Nam | Lead exposure | 0.63 | 3.98(-0.53 to 8.82) | 2.01 | 1.96 |
| Yemen | Lead exposure | 0.45 | 34.55(-5.11 to 71.01) | 2.96 | 31.58 |
| Zambia | Lead exposure | 0.51 | 6.07(-0.82 to 13.42) | 3.02 | 3.05 |
| Zimbabwe | Lead exposure | 0.47 | 8.72(-1.18 to 18.79) | 2.96 | 5.76 |

**Table S7**. Frontier DALYs, and effective difference by country or territory.

| **Location** | **Risk** | **SDI** | **Rate of DALYs** | **Frontier DALYs** | **Effective difference** |
| --- | --- | --- | --- | --- | --- |
| Afghanistan | Lead exposure | 0.34 | 758.43(-108.65 to 1617.92) | 66.32 | 692.11 |
| Albania | Lead exposure | 0.71 | 129.12(-17.67 to 290.33) | 25.66 | 103.46 |
| Algeria | Lead exposure | 0.66 | 248.79(-32.69 to 542.71) | 33.1 | 215.69 |
| American Samoa | Lead exposure | 0.72 | 50.93(-6.45 to 111.67) | 24.85 | 26.08 |
| Andorra | Lead exposure | 0.87 | 19.82(-2.5 to 43.93) | 11.92 | 7.9 |
| Angola | Lead exposure | 0.45 | 158.01(-21.37 to 335.97) | 58.67 | 99.34 |
| Antigua and Barbuda | Lead exposure | 0.75 | 63.33(-9.4 to 136.79) | 22.29 | 41.04 |
| Argentina | Lead exposure | 0.72 | 42.27(-5.88 to 93.91) | 23.55 | 18.72 |
| Armenia | Lead exposure | 0.70 | 151.55(-20.89 to 340.4) | 25.76 | 125.78 |
| Australia | Lead exposure | 0.84 | 39.17(-5.52 to 85.3) | 17.47 | 21.7 |
| Austria | Lead exposure | 0.85 | 33.41(-4.78 to 73.12) | 15.34 | 18.08 |
| Azerbaijan | Lead exposure | 0.69 | 200.78(-26.97 to 433.18) | 26.12 | 174.66 |
| Bahamas | Lead exposure | 0.81 | 48.69(-6.5 to 108.96) | 18.02 | 30.67 |
| Bahrain | Lead exposure | 0.75 | 138.9(-19.28 to 300.06) | 22.21 | 116.68 |
| Bangladesh | Lead exposure | 0.49 | 233.04(-34.26 to 501.41) | 59.94 | 173.1 |
| Barbados | Lead exposure | 0.75 | 47.32(-6.37 to 108.72) | 22.24 | 25.08 |
| Belarus | Lead exposure | 0.78 | 196.51(-26.06 to 423.18) | 17.91 | 178.6 |
| Belgium | Lead exposure | 0.85 | 43.42(-6.17 to 96.16) | 15.94 | 27.48 |
| Belize | Lead exposure | 0.61 | 64.42(-8.74 to 142.27) | 44.39 | 20.03 |
| Benin | Lead exposure | 0.37 | 101.48(-14.68 to 213.69) | 58.77 | 42.71 |
| Bermuda | Lead exposure | 0.82 | 35.37(-4.46 to 80.17) | 17.92 | 17.45 |
| Bhutan | Lead exposure | 0.47 | 248.83(-36.66 to 516.74) | 58.35 | 190.48 |
| Bolivia (Plurinational State of) | Lead exposure | 0.60 | 113.24(-14.98 to 252.08) | 45.75 | 67.48 |
| Bosnia and Herzegovina | Lead exposure | 0.72 | 139.87(-20.09 to 311.87) | 23.56 | 116.31 |
| Botswana | Lead exposure | 0.64 | 114.22(-13.97 to 248.75) | 34.53 | 79.7 |
| Brazil | Lead exposure | 0.65 | 81.28(-11.9 to 174.31) | 33.97 | 47.31 |
| Brunei Darussalam | Lead exposure | 0.81 | 63.24(-9.25 to 140.91) | 17.87 | 45.37 |
| Bulgaria | Lead exposure | 0.77 | 134.18(-18.41 to 292.78) | 19.88 | 114.3 |
| Burkina Faso | Lead exposure | 0.29 | 197.1(-27.27 to 414.8) | 74.66 | 122.43 |
| Burundi | Lead exposure | 0.29 | 141.89(-17.58 to 301.46) | 74.8 | 67.09 |
| Cabo Verde | Lead exposure | 0.53 | 129.81(-18.34 to 283.71) | 51.36 | 78.46 |
| Cambodia | Lead exposure | 0.47 | 144.48(-20.4 to 322.54) | 58.72 | 85.76 |
| Cameroon | Lead exposure | 0.48 | 157.64(-22.21 to 359.25) | 58.85 | 98.79 |
| Canada | Lead exposure | 0.87 | 23.69(-3.3 to 52.31) | 11.96 | 11.73 |
| Central African Republic | Lead exposure | 0.31 | 259.5(-40.68 to 545.3) | 74.73 | 184.77 |
| Chad | Lead exposure | 0.24 | 231.53(-32.54 to 485.2) | 74.72 | 156.8 |
| Chile | Lead exposure | 0.77 | 17.87(-2.48 to 38.21) | 17.85 | 0.02 |
| China | Lead exposure | 0.72 | 133.33(-18.62 to 291.17) | 24.09 | 109.24 |
| Colombia | Lead exposure | 0.66 | 90.99(-12.58 to 196.84) | 32.69 | 58.31 |
| Comoros | Lead exposure | 0.48 | 91.48(-12.17 to 190.52) | 59.92 | 31.56 |
| Congo | Lead exposure | 0.58 | 144.66(-19.37 to 324.99) | 49.69 | 94.97 |
| Cook Islands | Lead exposure | 0.78 | 29.4(-3.82 to 66.07) | 18.02 | 11.38 |
| Costa Rica | Lead exposure | 0.70 | 73.47(-10.22 to 156.5) | 25.73 | 47.74 |
| Coted'Ivoire | Lead exposure | 0.43 | 172.92(-24.91 to 380.23) | 59.95 | 112.97 |
| Croatia | Lead exposure | 0.80 | 79.51(-10.99 to 177.61) | 17.87 | 61.64 |
| Cuba | Lead exposure | 0.67 | 155.42(-22.32 to 323.37) | 32.07 | 123.35 |
| Cyprus | Lead exposure | 0.84 | 73.42(-10.66 to 158.64) | 17.88 | 55.54 |
| Czechia | Lead exposure | 0.83 | 57.11(-7.68 to 127.64) | 17.85 | 39.26 |
| Democratic People's Republic of Korea | Lead exposure | 0.57 | 151.53(-19.53 to 326) | 50.5 | 101.03 |
| Democratic Republic of the Congo | Lead exposure | 0.38 | 152.29(-22.51 to 329.03) | 58.85 | 93.44 |
| Denmark | Lead exposure | 0.90 | 22.95(-3.28 to 50.1) | 11.4 | 11.55 |
| Djibouti | Lead exposure | 0.49 | 129.39(-15.37 to 295.36) | 60.51 | 68.88 |
| Dominica | Lead exposure | 0.75 | 77.24(-10.92 to 167.33) | 22.17 | 55.07 |
| Dominican Republic | Lead exposure | 0.62 | 278.89(-36.36 to 601.33) | 39.88 | 239.01 |
| Ecuador | Lead exposure | 0.66 | 62.1(-8.46 to 138.43) | 32.24 | 29.86 |
| Egypt | Lead exposure | 0.61 | 739.8(-105.59 to 1550.05) | 41.58 | 698.23 |
| El Salvador | Lead exposure | 0.56 | 200.91(-28.01 to 429.09) | 50.81 | 150.09 |
| Equatorial Guinea | Lead exposure | 0.66 | 142.74(-19.15 to 328.18) | 32.72 | 110.03 |
| Eritrea | Lead exposure | 0.40 | 121.69(-15.48 to 267.61) | 58.92 | 62.77 |
| Estonia | Lead exposure | 0.84 | 38.21(-5.4 to 83.5) | 17.38 | 20.83 |
| Eswatini | Lead exposure | 0.59 | 154.79(-23.67 to 341.07) | 50.14 | 104.64 |
| Ethiopia | Lead exposure | 0.36 | 104.63(-13.79 to 228.22) | 59.89 | 44.74 |
| Fiji | Lead exposure | 0.68 | 112.18(-14.26 to 250.4) | 30.81 | 81.36 |
| Finland | Lead exposure | 0.86 | 22.91(-3.15 to 51.25) | 14.99 | 7.93 |
| France | Lead exposure | 0.84 | 21.58(-3.05 to 46.02) | 17.89 | 3.7 |
| Gabon | Lead exposure | 0.63 | 101.39(-13.05 to 220.93) | 35.82 | 65.56 |
| Gambia | Lead exposure | 0.41 | 221.11(-31.02 to 463.19) | 58.81 | 162.31 |
| Georgia | Lead exposure | 0.73 | 144.23(-21.52 to 310.64) | 25.13 | 119.1 |
| Germany | Lead exposure | 0.90 | 26.26(-3.54 to 58.02) | 11.37 | 14.88 |
| Ghana | Lead exposure | 0.56 | 94.59(-13.54 to 201.91) | 51.16 | 43.43 |
| Greece | Lead exposure | 0.79 | 69.83(-9.9 to 151.46) | 18.19 | 51.63 |
| Greenland | Lead exposure | 0.83 | 37.58(-5.04 to 83.6) | 17.91 | 19.67 |
| Grenada | Lead exposure | 0.67 | 129.31(-18.15 to 278.66) | 32.03 | 97.28 |
| Guam | Lead exposure | 0.80 | 37.2(-4.81 to 82.46) | 18.2 | 19 |
| Guatemala | Lead exposure | 0.54 | 209.5(-30.69 to 431.5) | 50.86 | 158.64 |
| Guinea | Lead exposure | 0.34 | 198.35(-27.68 to 410.39) | 66.33 | 132.02 |
| Guinea-Bissau | Lead exposure | 0.35 | 260.65(-34.68 to 549.62) | 60.51 | 200.14 |
| Guyana | Lead exposure | 0.65 | 222.38(-30.24 to 491.39) | 34.06 | 188.32 |
| Haiti | Lead exposure | 0.45 | 515.52(-78.02 to 1101.77) | 58.9 | 456.61 |
| Honduras | Lead exposure | 0.51 | 369.14(-52.07 to 767.45) | 59.28 | 309.86 |
| Hungary | Lead exposure | 0.79 | 100.82(-13.65 to 215.19) | 18.54 | 82.27 |
| Iceland | Lead exposure | 0.88 | 31.87(-4.18 to 70.23) | 11.8 | 20.08 |
| India | Lead exposure | 0.58 | 292.45(-41.34 to 613.26) | 50.79 | 241.66 |
| Indonesia | Lead exposure | 0.66 | 178.21(-26.58 to 386.32) | 32.92 | 145.29 |
| Iran (Islamic Republic of) | Lead exposure | 0.70 | 305.68(-45.15 to 652.83) | 27.54 | 278.14 |
| Iraq | Lead exposure | 0.66 | 342.89(-46.51 to 762.92) | 32.38 | 310.52 |
| Ireland | Lead exposure | 0.87 | 35.27(-4.98 to 78.27) | 11.87 | 23.4 |
| Israel | Lead exposure | 0.81 | 17.99(-2.51 to 39.39) | 17.87 | 0.11 |
| Italy | Lead exposure | 0.81 | 31.49(-4.39 to 69.14) | 18.26 | 13.23 |
| Jamaica | Lead exposure | 0.68 | 69.59(-9.18 to 152.34) | 29.32 | 40.27 |
| Japan | Lead exposure | 0.87 | 11.67(-1.63 to 25.77) | 11.67 | 0 |
| Jordan | Lead exposure | 0.73 | 117.11(-16.35 to 254.58) | 23.44 | 93.67 |
| Kazakhstan | Lead exposure | 0.73 | 111.42(-15.74 to 239.3) | 25.2 | 86.23 |
| Kenya | Lead exposure | 0.52 | 69.22(-9.8 to 160.92) | 51.21 | 18.01 |
| Kiribati | Lead exposure | 0.53 | 114.35(-14.53 to 254.96) | 51.19 | 63.17 |
| Kuwait | Lead exposure | 0.85 | 106.56(-14.95 to 226.78) | 17.31 | 89.25 |
| Kyrgyzstan | Lead exposure | 0.60 | 203.93(-28.26 to 451.24) | 44.86 | 159.06 |
| Lao People's Democratic Republic | Lead exposure | 0.49 | 261.68(-38.03 to 569.39) | 59.76 | 201.93 |
| Latvia | Lead exposure | 0.83 | 74.06(-10.21 to 159.9) | 17.87 | 56.2 |
| Lebanon | Lead exposure | 0.74 | 76.82(-10.1 to 166.74) | 23.06 | 53.75 |
| Lesotho | Lead exposure | 0.51 | 148.36(-19.69 to 342.68) | 58.75 | 89.62 |
| Liberia | Lead exposure | 0.35 | 182.16(-26.03 to 391.66) | 58.66 | 123.5 |
| Libya | Lead exposure | 0.73 | 232.89(-30.25 to 517.76) | 23.55 | 209.34 |
| Lithuania | Lead exposure | 0.86 | 84.49(-12.12 to 184.28) | 15.97 | 68.52 |
| Luxembourg | Lead exposure | 0.88 | 24.61(-3.27 to 54.95) | 11.95 | 12.66 |
| Madagascar | Lead exposure | 0.40 | 133.2(-15.92 to 298.07) | 59.69 | 73.5 |
| Malawi | Lead exposure | 0.38 | 136.23(-19.07 to 283.29) | 58.67 | 77.56 |
| Malaysia | Lead exposure | 0.74 | 143.65(-20.25 to 302.3) | 22.95 | 120.7 |
| Maldives | Lead exposure | 0.65 | 79.22(-10.1 to 170.46) | 34.13 | 45.1 |
| Mali | Lead exposure | 0.27 | 144.98(-20.92 to 314.25) | 74.76 | 70.23 |
| Malta | Lead exposure | 0.80 | 107.6(-15.79 to 224.91) | 18.09 | 89.51 |
| Marshall Islands | Lead exposure | 0.57 | 144.44(-20.92 to 319.28) | 50.71 | 93.73 |
| Mauritania | Lead exposure | 0.50 | 111.31(-14.55 to 241.53) | 59.62 | 51.69 |
| Mauritius | Lead exposure | 0.72 | 66.42(-8.85 to 146.73) | 24.92 | 41.5 |
| Mexico | Lead exposure | 0.66 | 154.78(-22.74 to 332.82) | 32.6 | 122.18 |
| Micronesia (Federated States of) | Lead exposure | 0.59 | 132.71(-18.94 to 300.01) | 45.31 | 87.41 |
| Monaco | Lead exposure | 0.91 | 25.21(-3.51 to 55.63) | 11.45 | 13.76 |
| Mongolia | Lead exposure | 0.62 | 201.27(-25.66 to 434.54) | 39.33 | 161.93 |
| Montenegro | Lead exposure | 0.80 | 104.79(-13.9 to 230.38) | 18.07 | 86.72 |
| Morocco | Lead exposure | 0.56 | 311.92(-39.7 to 677.12) | 50.71 | 261.21 |
| Mozambique | Lead exposure | 0.33 | 98.53(-13.44 to 206.13) | 74.72 | 23.81 |
| Myanmar | Lead exposure | 0.53 | 173.87(-23.61 to 376.66) | 51.48 | 122.38 |
| Namibia | Lead exposure | 0.62 | 122.32(-16.48 to 270.99) | 39.77 | 82.55 |
| Nauru | Lead exposure | 0.63 | 161.61(-21.67 to 359.1) | 38.95 | 122.65 |
| Nepal | Lead exposure | 0.43 | 338.97(-45.3 to 706.03) | 58.7 | 280.27 |
| Netherlands | Lead exposure | 0.89 | 20.72(-2.82 to 46.05) | 11.39 | 9.33 |
| New Zealand | Lead exposure | 0.85 | 52.33(-7.56 to 113.46) | 16.84 | 35.49 |
| Nicaragua | Lead exposure | 0.52 | 131.26(-19.22 to 275.14) | 52.85 | 78.41 |
| Niger | Lead exposure | 0.17 | 164.93(-24.9 to 353.47) | 133.58 | 31.35 |
| Nigeria | Lead exposure | 0.50 | 110.06(-13.91 to 241.96) | 60.63 | 49.44 |
| Niue | Lead exposure | 0.73 | 73.22(-9.72 to 163.45) | 24.81 | 48.41 |
| North Macedonia | Lead exposure | 0.75 | 123.77(-16.29 to 274.69) | 22.1 | 101.67 |
| Northern Mariana Islands | Lead exposure | 0.77 | 36.12(-4.59 to 80.14) | 19.09 | 17.03 |
| Norway | Lead exposure | 0.92 | 22.65(-3.13 to 50.06) | 11.48 | 11.17 |
| Oman | Lead exposure | 0.77 | 197.09(-25.81 to 444.32) | 18.18 | 178.91 |
| Pakistan | Lead exposure | 0.50 | 370.02(-56.44 to 799.58) | 58.84 | 311.18 |
| Palau | Lead exposure | 0.75 | 58.14(-7.56 to 125.61) | 22.11 | 36.03 |
| Palestine | Lead exposure | 0.63 | 282.06(-40.44 to 608.58) | 36.26 | 245.8 |
| Panama | Lead exposure | 0.71 | 70.58(-9.79 to 156.23) | 24.96 | 45.62 |
| Papua New Guinea | Lead exposure | 0.42 | 85.4(-10.68 to 195.89) | 58.51 | 26.89 |
| Paraguay | Lead exposure | 0.64 | 93.21(-12.15 to 208.29) | 36.35 | 56.86 |
| Peru | Lead exposure | 0.66 | 51.64(-6.79 to 115.81) | 32.44 | 19.2 |
| Philippines | Lead exposure | 0.65 | 125.04(-16.79 to 275.09) | 33.76 | 91.28 |
| Poland | Lead exposure | 0.81 | 83.56(-12.22 to 182.3) | 17.93 | 65.63 |
| Portugal | Lead exposure | 0.74 | 45.03(-6.19 to 100.39) | 22.44 | 22.59 |
| Puerto Rico | Lead exposure | 0.83 | 36.27(-4.94 to 79.28) | 17.85 | 18.42 |
| Qatar | Lead exposure | 0.85 | 83.1(-11.68 to 183.35) | 16.88 | 66.23 |
| Republic of Korea | Lead exposure | 0.89 | 20.97(-2.98 to 47.6) | 11.85 | 9.11 |
| Republic of Moldova | Lead exposure | 0.73 | 149.71(-20.6 to 319.64) | 23.49 | 126.21 |
| Romania | Lead exposure | 0.77 | 105.56(-15.52 to 232.71) | 19.93 | 85.64 |
| Russian Federation | Lead exposure | 0.81 | 109.33(-14.09 to 238.82) | 18.47 | 90.87 |
| Rwanda | Lead exposure | 0.44 | 84.92(-10.35 to 185.11) | 59.77 | 25.15 |
| Saint Kitts and Nevis | Lead exposure | 0.75 | 82.21(-11.15 to 182.8) | 21.83 | 60.38 |
| Saint Lucia | Lead exposure | 0.67 | 65.73(-9.32 to 146.67) | 32.09 | 33.65 |
| Saint Vincent and the Grenadines | Lead exposure | 0.64 | 146.11(-20 to 321.7) | 36.19 | 109.92 |
| Samoa | Lead exposure | 0.59 | 93.68(-12.14 to 205.7) | 45.09 | 48.59 |
| San Marino | Lead exposure | 0.89 | 11.28(-1.41 to 25.26) | 11.28 | 0 |
| Sao Tome and Principe | Lead exposure | 0.51 | 129.79(-15.94 to 284.55) | 59.67 | 70.13 |
| Saudi Arabia | Lead exposure | 0.82 | 270.56(-37.33 to 579.44) | 17.95 | 252.61 |
| Senegal | Lead exposure | 0.41 | 146.13(-21.82 to 325.93) | 59.31 | 86.82 |
| Serbia | Lead exposure | 0.79 | 106.67(-14.87 to 228.69) | 18.02 | 88.65 |
| Seychelles | Lead exposure | 0.73 | 46.75(-6.33 to 103.11) | 23.74 | 23.01 |
| Sierra Leone | Lead exposure | 0.36 | 205.34(-30.04 to 434.84) | 59.04 | 146.3 |
| Singapore | Lead exposure | 0.86 | 48.32(-6.72 to 103.61) | 14.91 | 33.4 |
| Slovakia | Lead exposure | 0.81 | 94.72(-12.84 to 208.63) | 17.89 | 76.83 |
| Slovenia | Lead exposure | 0.84 | 23.13(-3.04 to 51.2) | 17.56 | 5.57 |
| Solomon Islands | Lead exposure | 0.43 | 267.69(-36.35 to 592.5) | 59.7 | 207.99 |
| Somalia | Lead exposure | 0.08 | 180.24(-29.75 to 383.87) | 164.47 | 15.77 |
| South Africa | Lead exposure | 0.68 | 70.49(-9.4 to 152.68) | 30.74 | 39.75 |
| South Sudan | Lead exposure | 0.28 | 130.01(-18.38 to 277.14) | 74.68 | 55.33 |
| Spain | Lead exposure | 0.77 | 34.9(-4.94 to 76.39) | 18.86 | 16.04 |
| Sri Lanka | Lead exposure | 0.70 | 58.65(-7.24 to 136.51) | 26.32 | 32.33 |
| Sudan | Lead exposure | 0.54 | 549.93(-73.04 to 1217.62) | 50.59 | 499.34 |
| Suriname | Lead exposure | 0.63 | 125.8(-18.07 to 283.82) | 36.58 | 89.22 |
| Sweden | Lead exposure | 0.89 | 19.05(-2.65 to 42.06) | 11.84 | 7.21 |
| Switzerland | Lead exposure | 0.93 | 25.05(-3.47 to 55.54) | 12.25 | 12.79 |
| Syrian Arab Republic | Lead exposure | 0.62 | 553.91(-77.34 to 1160.33) | 39.06 | 514.85 |
| Taiwan (Province of China) | Lead exposure | 0.87 | 35.78(-5.13 to 78.53) | 11.97 | 23.8 |
| Tajikistan | Lead exposure | 0.54 | 251.01(-37.93 to 548.25) | 51.18 | 199.83 |
| Thailand | Lead exposure | 0.68 | 30.9(-3.87 to 69.64) | 30.74 | 0.16 |
| Timor-Leste | Lead exposure | 0.44 | 245.72(-32.71 to 539.46) | 59.73 | 185.99 |
| Togo | Lead exposure | 0.41 | 158.03(-21.09 to 343.28) | 58.92 | 99.1 |
| Tokelau | Lead exposure | 0.69 | 72.31(-8.82 to 161.05) | 28.84 | 43.47 |
| Tonga | Lead exposure | 0.63 | 65.52(-8.08 to 145.94) | 37.46 | 28.06 |
| Trinidad and Tobago | Lead exposure | 0.77 | 65.76(-8.47 to 147.27) | 20.81 | 44.95 |
| Tunisia | Lead exposure | 0.68 | 210.74(-26.65 to 465.93) | 30.84 | 179.9 |
| Turkey | Lead exposure | 0.71 | 113.46(-15.75 to 250.87) | 25.41 | 88.05 |
| Turkmenistan | Lead exposure | 0.68 | 214.81(-29.47 to 465.57) | 30.84 | 183.97 |
| Tuvalu | Lead exposure | 0.58 | 150.18(-21.25 to 326.39) | 50.71 | 99.47 |
| Uganda | Lead exposure | 0.42 | 108.47(-14.48 to 230.31) | 58.71 | 49.76 |
| Ukraine | Lead exposure | 0.76 | 142.32(-17.68 to 331.61) | 19.86 | 122.46 |
| United Arab Emirates | Lead exposure | 0.85 | 122.07(-17.01 to 268.31) | 16.73 | 105.34 |
| United Kingdom | Lead exposure | 0.86 | 27.99(-3.86 to 60.78) | 15.23 | 12.76 |
| United Republic of Tanzania | Lead exposure | 0.45 | 102.79(-11.66 to 224.82) | 58.54 | 44.25 |
| United States Virgin Islands | Lead exposure | 0.82 | 56.62(-7.58 to 126.24) | 17.91 | 38.71 |
| United States of America | Lead exposure | 0.86 | 42.23(-5.86 to 90.76) | 14.9 | 27.32 |
| Uruguay | Lead exposure | 0.72 | 61.28(-8.72 to 133.64) | 24.87 | 36.42 |
| Uzbekistan | Lead exposure | 0.66 | 218.68(-29.68 to 477.17) | 32.46 | 186.22 |
| Vanuatu | Lead exposure | 0.47 | 269.92(-37.11 to 599.93) | 60.15 | 209.77 |
| Venezuela (Bolivarian Republic of) | Lead exposure | 0.60 | 222.2(-30.93 to 498.01) | 44.87 | 177.34 |
| Viet Nam | Lead exposure | 0.63 | 72.66(-9.48 to 162.56) | 39.08 | 33.58 |
| Yemen | Lead exposure | 0.45 | 665.77(-95.1 to 1395.8) | 58.85 | 606.92 |
| Zambia | Lead exposure | 0.51 | 113.43(-14.84 to 250.38) | 58.74 | 54.7 |
| Zimbabwe | Lead exposure | 0.47 | 172.3(-22.84 to 367.29) | 59.79 | 112.51 |
